# Safety and immunogenicity of an HIV vaccine trial with DNA prime and replicating vaccinia boost

**DOI:** 10.1038/s41392-025-02259-y

**Published:** 2025-07-02

**Authors:** Ying Liu, Wei Lv, Pu Shan, Dan Li, Ying-Qi Wu, You-Chun Wang, Yuan-Yuan Li, Qiang Liu, Jian-Sheng Wang, Yan-Ling Hao, Yong Liu, Wei-Jin Huang, Li Ren, Shu-Hui Wang, Tai-Sheng Li, Jing Xu, Yi-Ming Shao

**Affiliations:** 1 National Key Laboratory of Intelligent Tracking and Forecasting for Infectious Diseases, National Center for AIDS/STD Control and Prevention, Chinese Center for Disease Control and Prevention, Beijing, China; 2 Department of Infectious Diseases, Peking Union Medical College Hospital, Peking Union Medical College, Chinese Academy of Medical Sciences, Beijing, China; 3 State Key Laboratory for Complex, Severe and Rare Diseases, Peking Union Medical College Hospital, Beijing, China; 4 National Vaccine & Serum Institute, Beijing, China; 5 National Institutes for Food and Drug Control, Beijing, China; 6 Division of Health Statistics, Chinese Center for Disease Control and Prevention, Beijing, China

**Keywords:** Vaccines, Vaccines

## Abstract

Developing a safe and effective vaccine remains a global priority for ending the human immunodeficiency virus (HIV) pandemic. All HIV vaccine trials with protein, DNA, non-replication vector or their combinations failed in the past. We constructed the HIV-1 CN54 *env*, *gag*, and *pol* genes into both DNA and replicating vaccinia virus Tiantan vectors. In phase Ia, 12 healthy adults were given high (*n* = 6) or low (*n* = 6) doses of recombinant vaccinia virus Tiantan vaccine (rTV), to test its safety dose. In phase Ib, 36 healthy adults were assigned to the DNA (*n* = 6), DNA-L/rTV (*n* = 12), DNA-H/rTV (*n* = 12), and placebo (*n* = 6) groups. The DNA vaccine was injected intramuscularly at weeks 0, 4, and 8 and rTV with a bifurcated needle at week 12. All vaccines tested were safe and well-tolerated; most of the adverse events (AEs) were mild to moderate. The most commonly observed AEs were redness and papule at rTV vaccination sites and axillary enlarged lymph nodes at the same rTV vaccination arm. Smaller cutaneous lesions and shorter healing time were observed in smallpox vaccine experienced subjects. The DNA prime-rTV boost regimen induced anti-gp120 IgG and polyfunctional CD4^+^ T cells. No significant differences of anti-HIV IgG and T cell responses were found between the two prime-boost groups with high and low DNA doses. Moreover, smallpox vaccine naïve subjects elicited higher T cell responses and anti-gp120 antibodies. The result of this trial supports further development of HIV vaccine with DNA and replicating vaccinia vector for advanced clinical trials.

## Introduction

The global HIV/AIDS pandemic remains one of the most significant public health challenges for us. Despite significant advancements in antiretroviral therapy (ART) and pre-exposure prophylaxis, ~1.3 million people become infected with the human immunodeficiency virus (HIV) every year.^1^ This epidemiological paradox underscores the critical need for an effective prophylactic vaccine – the only sustainable solution for epidemic control.^2^ Unlike many pathogens successfully controlled through vaccination, HIV presents great challenges for vaccine design, including extraordinary genetic diversity, rapid mutation rates, genomic integration, viral reservoirs, and sophisticated immune evasion mechanisms. Over three decades of HIV vaccine research have yielded important insights but limited clinical success. More than 300 HIV vaccine trials have been conducted, including 11 efficacy trials, with most failing to demonstrate protective immunity.^3^ Of 300+ clinical trials conducted since 1987, only RV144 demonstrated modest 31% efficacy, using a prime-boost regimen of replication-incompetent recombinant canarypox-ALVAC-HIV (vCP1521) and alum-adjuvanted AIDSVAX subtypes B/E HIV envelope gp120.^4^ Shortly after RV144, the Pox-Protein Public Private Partnership (P5) developed an RV144-analogous efficacy trial in South Africa (HVTN 702) to test and prospectively define correlates of protection, incorporating the regionally predominant subtype C strain and MF59 adjuvant.^5,6^ However, the HVTN 702 trial did not confirm the results from RV144 and was halted for lack of protection. The estimated HIV hazard ratio was 1.02 for the first 24 months of follow-up.^6^ These contradictory outcomes emphasized the need to better understand regional immunological landscapes and subtype-specific antigenic variations. To elicit stronger and broader immune responses, recent advances in vaccine design have focused on inducing cross-reactive immune responses. Polyvalent mosaic antigens were designed and delivered by another replication-incompetent recombinant adenovirus serotype 26 (Ad26) vector, which induced robust cellular immune responses in non-human primates, and reduced per-exposure infection risk by 94%.^7,8^ However, in the phase IIb/III clinical trials, HVTN 705 and 706, the investigational regimen consisting of Ad26.Mos4.HIV and clade C gp140 protein failed to offer protection from HIV infection.^9^ All these attempts in HIV vaccine research referred to the fact that vaccine-induced immunity and durability needed to be further improved.

Compared to replication-incompetent vectors that provide transient antigen exposure, replication-competent viral vectors are promising alternatives for HIV antigen delivery because they have the ability to replicate in humans for a short time to prolong antigen exposure and activate adaptive immunity.^10^ Adenoviruses (Ad), vaccinia virus, cytomegalovirus, vesicular stomatitis virus, Sendai virus (SeV), and other replication-competent viral vectors have all been studied in preclinical macaque models or clinical trials, and each offers distinct advantages for the development of vaccine.^10^ For example, a replication-competent adenovirus-vectored influenza vaccine (Ad4-H5-Vtn) elicited durable systemic and mucosal immunity.^11^ Notably, recipients exhibited prolonged memory B cell response accompanied by increasing in H5-specific antibody somatic hypermutation, suggesting affinity maturation over time.^12^ A prime-boost strategy that includes a replicating vector could result in potent, long-lasting immunity.^13^ A replication-competent SeV-vectored HIV vaccine induced potent T-cell and antibody responses in prime-boost regimens: responses persisted for ≥8 months, and featured functional CD8^+^ T-cell responses with greater breadth, magnitude, and frequency.^14^ Replication-competent NYVAC-KC vectors have been tested safely in macaques and were found to be more potent inducers of cellular and humoral immunity when compared to vectors based on nonreplicating NYVAC.^10^ These study reveals that transient antigen exposure from replication-incompetent vectors fails to sustain germinal center reactions essential for antibody maturation and powerful T-cell responses. This impasse has redirected attention toward replication-competent vectors that mimic natural infection kinetics. By maintaining antigen expression over weeks rather than days, these platforms enable prolonged dendritic cell-T cell interactions in lymphoid tissues, driving robust memory formation and affinity maturation.

As a smallpox vaccine, vaccinia virus Tiantan was previously employed in China to eradicate smallpox before the 1980s, establishing safety profile with over 100 million doses administered. Its large genome (~190 kb) could accommodate multiple HIV genes (env/gag/pol), while its replication ability enables prolonged immune stimulation. Replication-competent poxvirus stimulates both localized innate immunity through epidermal scarification (recruiting Langerhans cells and dermal DCs) and systemic adaptive responses via prolonged antigen presentation.^15^ Notably, antibodies specific for the vaccinia virus are still detectable after at least 45 years from immunization.^16^ Preclinical studies demonstrated that a DNA prime-recombinant vaccinia virus Tiantan (rTV) boost regimen, encoding HIV gag, pol, and env, effectively prevented Chinese rhesus macaques from SHIVCN97001 infection or resulted in lower viremia than controls.^17^ An often-overlooked factor in vaccinia-based strategies is the impact of prior smallpox vaccination. Adults born before 1980 in China bear vaccinia-specific immunity from childhood immunization, which could theoretically influence HIV-specific responses. This complex immunoregulatory variables requiring clinical evaluation.

This phase I, first-in-human clinical trial investigated a replication-competent vaccinia Tiantan-based HIV candidate vaccine administered in combined with a DNA vaccine component. This randomized, placebo-controlled study addresses: (1) safety profile of replicating Tiantan vector in vaccinia-naive vs smallpox vaccinated subjects; (2) capacity to induce durable HIV-specific cellular and humoral responses through DNA prime/rTV boost regimens, and (3) immunomodulatory effects of pre-existing anti-vaccinia immunity.

## Results

### Study subjects and trial design

**Fig. 1:**
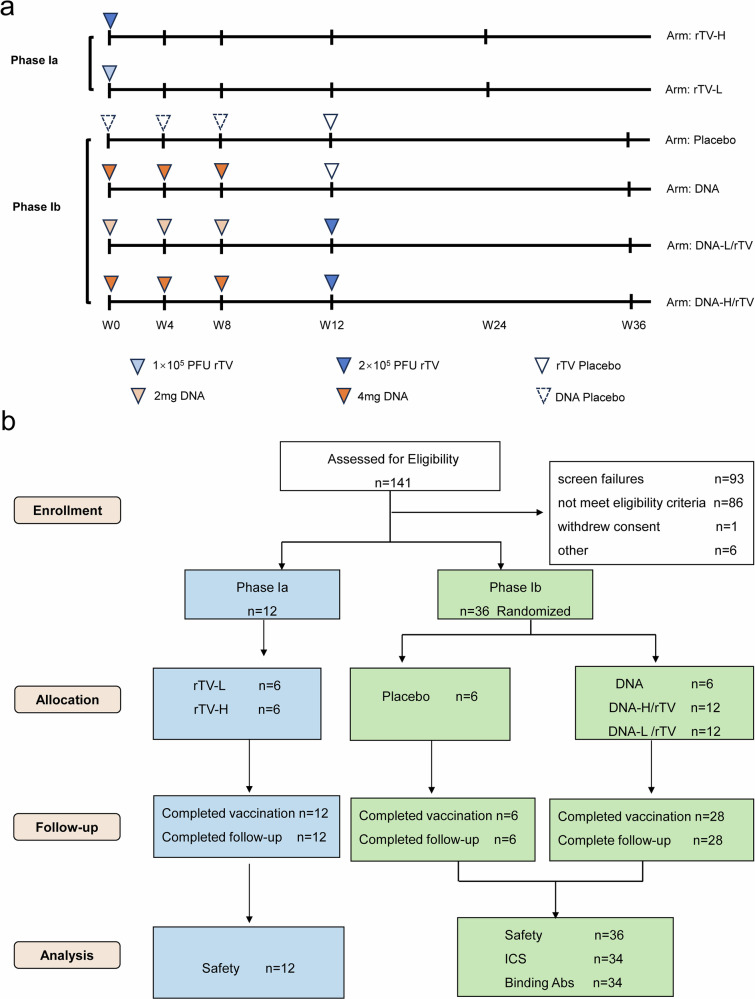
Study design and CONSORT diagram. **a** Study design. **b** CONSORT diagram. ICS: intracellular staining

A total of 141 volunteers were recruited and screened for eligibility, among which 48 subjects were sequentially enrolled and randomly assigned: 12 in phase Ia received rTV, and 36 in phase Ib, including 24 receiving DNA/rTV, 6 receiving DNA, and 6 receiving placebos (Fig. 1a). All study subjects were Chinese, with 46 (96%), 1 (2%) and 1 (2%) being Han, Mongolian, and Manchu ethnicities, respectively. There were 11 women (23%), and the median age of the subjects was 26.3 (19.7–44.7) years. Among the subjects, 30 were vaccinia-naive (born after 1980, no vaccination scar), and 18 were non-naïve (Table 1). Randomization and follow-up of subjects is summarized in Fig. 1b. All but two of the 48 subjects received all vaccinations and completed follow-up; these two subjects, who both requested to early withdraw from the study, one (DNA-H/rTV group) received one dose of DNA injection, and another (DNA-L/rTV group) did not receive the third dose of DNA and rTV vaccination.

**Table 1:** Demographic Characteristics of the Participants

Groups	Ia		Ib				Total (*n* = 48)
	rTV-L	rTV-H	DNA-L/ rTV	DNA-H/ rTV	DNA	Placebo	
No. of participants	6	6	12	12	6	6	48
Gender—no. (%)							
Male	6 (100%)	5 (83%)	8 (67%)	10 (83%)	4 (67%)	4 (67%)	37 (77%)
Female	0 (.)	1 (17%)	4 (33%)	2 (17%)	2 (33%)	2 (33%)	11 (23%)
Ethnic—no. (%)							
Han	6 (100%)	5 (83%)	12 (100%)	12 (100%)	5 (83%)	6 (100%)	46 (96%)
Mongolian	0 (.)	1 (17%)	0 (.)	0 (.)	0 (.)	0 (.)	1 (2%)
Manchu	0 (.)	0 (.)	0 (.)	0 (.)	1 (16.7%)	0 (.)	1 (2%)
Age—yr							
Median (range)	25.2 (20.0–29.0)	28.7 (23.6–39.2)	24.8 (19.7–44.7)	26.6 (22.4–31.4)	28.5 (23.1–36.6)	25.9 (21.0–40.6)	26.3 (19.7–44.7)
Smallpox vaccination status - no. (%)							
Naïve	4 (67%)	3 (50%)	7 (58%)	8 (67%)	3 (50%)	5 (83%)	30 (63%)
Non-Naive	2 (33%)	3 (50%)	5 (42%)	4 (33%)	3 (50%)	1 (17%)	18 (38%)

### Safety

Overall, the HIV DNA and rTV vaccines were well tolerated. All (12/12) subjects receiving rTV and 88% (21/24) of subjects receiving DNA and rTV reported adverse events (AEs) during the duration of the trial. The incidence of AE in both DNA group and placebo group was 67% (Table 2). The majority of AEs were mild to moderate (Grade 1-2), with spontaneous resolution within 30 days without medical intervention. No subjects reported any serious adverse events (SAEs), nor withdrew from the study due to vaccination or AEs. There were five subjects reporting five cases of Grade 3 AEs. One subject in rTV-H group developed fever (39.5 °C) at 2 months after rTV vaccination due to upper respiratory infection. One case of upper respiratory infection occurred in subject (DNA-H/rTV group) at 25 days after rTV vaccination. The subject reported experiencing influenza-like symptoms at 22 days post-vaccination, with symptoms resolving within 24 h following symptomatic treatment. Two subjects in DNA group developed laboratory tests abnormal. One was proteinuria at 6 months after rTV placebo vaccination and another was elevated creatine kinase (CK) at 3 months after rTV placebo vaccination. One subject in DNA-L/rTV group developed proteinuria at 7 days after rTV vaccination. The elevated CK level and proteinuria were asymptomatic, resolved within 1 month, and the subjects continued in the trial without further issue. All these Grade 3 AEs were considered not related to rTV vaccine or placebo by the investigators. AEs related to vaccine or placebo were reported by 75% (9/12) of the subjects who received rTV alone, by 58% (14/24) of the subjects who received DNA and rTV, by 33% (2/6) of the subjects who received DNA alone, as compared with 17% (1/6) of those who received placebo.

**Table 2:** Frequency and severity of adverse events

	Ia		Ib			
	rTV-L (*n* = 6)	rTV-H (*n* = 6)	DNA-L/rTV (*n* = 12)	DNA-H/rTV (*n* = 12)	DNA (*n* = 6)	Placebo (*n* = 6)
Any AE	6 (100%)	6 (100%)	10 (83%)	11 (92%)	4 (67%)	4 (67%)
Related^a^	4 (67%)	5 (83%)	7 (58%)	7 (58%)	2 (33%)	1 (17%)
Grade 3	0 (.)	1 (17%)	1 (8%)	1 (8%)	2 (33%)	0 (.)
Any SAE	0 (.)	0 (.)	0 (.)	0 (.)	0 (.)	0 (.)
Any AE leading to withdrawal	0 (.)	0 (.)	0 (.)	0 (.)	0 (.)	0 (.)

All data are *n* (%)

*AE* adverse event, *SAE* serious adverse event

^a^The adverse event definitely, probably, or possibly related to study vaccine or vaccination considered by the investigators

AEs within 28 days after each vaccination were shown in Table 3. The most common AEs were reactogenicity signs or symptoms. Fourteen subjects who received DNA and rTV reported mild-to-moderate local adverse reactions, primarily redness (7 cases) and/or papule (9 cases) at the inoculation site in 3–12 days after rTV vaccination. No local adverse reactions were observed in subjects who received rTV alone, DNA alone, or placebo. The most frequently reported systemic reactogenicity events were lymph node enlargement with or without tenderness, with 12 subjects reporting in 3–11 days after rTV vaccination and 2 subjects reporting on day 6 or 28 after DNA vaccination. Other systemic reactogenicity symptoms included low-grade fever (<38 °C, two cases in rTV-L group), headache (two cases in DNA group) and arthralgia (one case in placebo group). All of the reactogenicity symptoms were mild and transient, and generally resolved in about seven days without treatment. Upper respiratory infection was a common unexpected AE in this study, and the frequency/severity did not differ between groups. A total of 13 subjects reported upper respiratory infection within 28 days after each of the vaccination, with 1, 6, and 6 cases occurred after placebo, DNA and rTV vaccinations, respectively. Moreover, the infections were clustered between October and January, a season of high prevalence for upper respiratory tract infections. All cases resolved spontaneously or after taking proper medicine and were assessed by clinical investigators as unrelated to the vaccine. In addition, we observed a few abnormal laboratory tests related to vaccines, deemed by the investigator. Three subjects in Ia developed elevated CK level in 1–4 weeks following rTV vaccination (Table 3). The elevated CK appeared on day 7 (01006, 347 U/L, Grade 1), day 14 (02410, 266 U/L, Grade 1), and day 28 (02511, 289 U/L, Grade 1). Even though none of the three subjects reported any discomfort symptoms, we still tested their CK isoenzyme associated with the myocardium (CK-MB) and electrocardiogram (ECG). The results were all normal with CK-MB 0% (normal range: 0–4%) and ECG showed no abnormalities. Subjects 01006 and 02511 reported engaging in exercise within 48 h prior to CK measurement. The CK levels of the three subjects returned to the normal range during follow-up monitoring test. The clinical investigator concluded that the observed CK elevations were consistent with exercise-induced skeletal muscle microtrauma rather than vaccine-related cardiotoxicity. While troponin levels were not assayed during phase Ia, comprehensive cardiac monitoring was implemented in phase Ib, including troponin measurements at pre-vaccination baseline and post-vaccination timepoints (days 3, 7, 14, 28, and 56). Serial assessments demonstrated no clinically significant deviations in either CK-MB or troponin among all subjects in phase Ib. Three subjects developed antinuclear antibody (ANA) positive (1:160) following DNA vaccination. One subject in rTV-H group developed a transient decrease in white blood cell (WBC) count on day 28 following rTV vaccination. These laboratory tests abnormal were asymptomatic and self-limiting, resolved in 1–4 weeks.

**Table 3:** Adverse events within 28 days after each vaccination

	Ia		Ib			
	rTV-L (*n* = 6)	rTV-H (*n* = 6)	DNA-L/ rTV (*n* = 12)	DNA-H/ rTV (*n* = 12)	DNA (*n* = 6)	Placebo (*n* = 6)
Reactogenicity						
*Local adverse reactions*						
Redness	0 (.)	0 (.)	1 (8%)	6 (50%)	0 (.)	0 (.)
Papule	0 (.)	0 (.)	6 (50%)	3 (25%)	0 (.)	0 (.)
*Systemic Reactogenicity events*						
Lymph node enlargement, with or without tenderness	4 (67%)	4 (67%)	4 (33%)	1(8%)	0 (.)	1(17%)
Fever^a^	1 (17%)	1 (17%)	0 (.)	0 (.)	0 (.)	0 (.)
Headache	0 (.)	0 (.)	0 (.)	0 (.)	1 (17%)	0 (.)
Arthralgia	0 (.)	0 (.)	0 (.)	0 (.)	0 (.)	1 (17%)
Unexpected AEs						
Upper respiratory infection	1 (17%)	0 (.)	4 (33%)	6 (50%)	1 (17%)	1 (17%)
Dizzy	0 (.)	1 (17%)	0 (.)	1(8%)	0 (.)	0 (.)
Rash	0 (.)	0 (.)	0 (.)	1(8%)	0 (.)	0 (.)
Acute tonsilitis	1 (17%)	0 (.)	0 (.)	1(8%)	0 (.)	0 (.)
Mouth ulcer	1 (17%)	0 (.)	0 (.)	1(8%)	0 (.)	0 (.)
Injury	1 (17%)	0 (.)	0 (.)	1(8%)	0 (.)	0 (.)
Cough	0 (.)	0 (.)	0 (.)	1(8%)	0 (.)	0 (.)
Pharyngitis	0 (.)	0 (.)	0 (.)	0 (.)	0 (.)	1 (17%)
Pericoronitis	0 (.)	0 (.)	1 (8%)	0 (.)	0 (.)	0 (.)
Tympanitis	0 (.)	0 (.)	0 (.)	1 (8%)	0 (.)	0 (.)
Chronic gastritis	0 (.)	0 (.)	0 (.)	0 (.)	1 (17%)	0 (.)
Stomachache	0 (.)	0 (.)	0 (.)	0 (.)	0 (.)	1 (17%)
Toothache	0 (.)	0 (.)	0 (.)	0 (.)	1 (17%)	0 (.)
CK elevation	2 (33%)	1(17%)	0 (.)	0 (.)	0 (.)	0 (.)
Leukopenia	0 (.)	0 (.)	0 (.)	1 (8%)	0 (.)	0 (.)
ANA (1:160)	0 (.)	0 (.)	1 (8%)	1 (8%)	1 (17%)	0 (.)

All data are *n* (%)

*AE* adverse event, *CK* creatine phosphokinase, *WBC* white blood cell, *ANA* antinuclear antibody

^a^The temperature is below 38 °C

After rTV vaccination, we observed the expected cutaneous responses at the site of inoculation. The skin lesions evolved gradually, with the appearance of redness and papules at the site of vaccination after 3–7 days. The papule became vesicular lesion, then pustule, and reaches its maximum size in 7–14 days (Table 4). The pustule then dried and formed a scab, which usually separated within 21–28 days. Both vaccinia-naïve and non-naïve subjects have the same stages of healing for a significant cutaneous reaction. However, the cutaneous lesions were smaller and healed faster in non-naive subjects. No local satellite lesions near the site of inoculation were observed among subjects receiving rTV.

**Table 4:** Local signs among participants after rTV vaccination

Local Signs	Vaccinia-Naïve (*n* = 21)				Non-Naive (*n* = 13)			
	Time After Vaccination, d							
	3	7	14	28	3	7	14	28
Redness								
Mean, mm	5.6	21.7	14.8	30.0	10.9	16.9	10.2	0
Range, mm	3–15	6–53	3–30	30	3–19	7–40	7–15	0
NO. of participants (%)	10(48%)	14(67%)	9(43%)	1(5%)	4(31%)	9(69%)	3(23%)	0(0)
Papula								
Mean, mm	7.3	8.5	8.3	8.5	8.2	8.1	9.0	0
Range, mm	4–12	4–20	5–12	7–10	4–11	4–20	5–14	0
NO. of participants (%)	6(29%)	17(81%)	17(81%)	2(10%)	7(54%)	10(80%)	4(31%)	0(0)
Vesicular lesion								
Mean, mm	0	5.2	10.0	0	0	4.0	0	0
Range, mm	0	4–7	10	0	0	2–5	0	0
NO. of participants (%)	0(0)	5(24%)	1(5%)	0(0)	0(0)	3(23%)	0(0)	0(0)
Pustule								
Mean, mm	0	5.2	7.5	0	0	5.4	7.5	0
Range, mm	0	2–8	5–10	0	0	2–19	6–9	0
NO. of participants (%)	0(0)	11(52%)	6(29%)	0(0)	0(0)	6(46%)	2(15%)	0(0)
Scab								
NO. of participants (%)	0(0)	2(10%)	14(67%)	11(52%)	0(0)	2(15%)	9(69%)	2(15%)
Scab separation								
NO. of participants (%)	0(0)	0(0)	4(19%)	13(62%)	0(0)	0(0)	4(31%)	9(69%)

### Immune responses

**Fig. 2:**
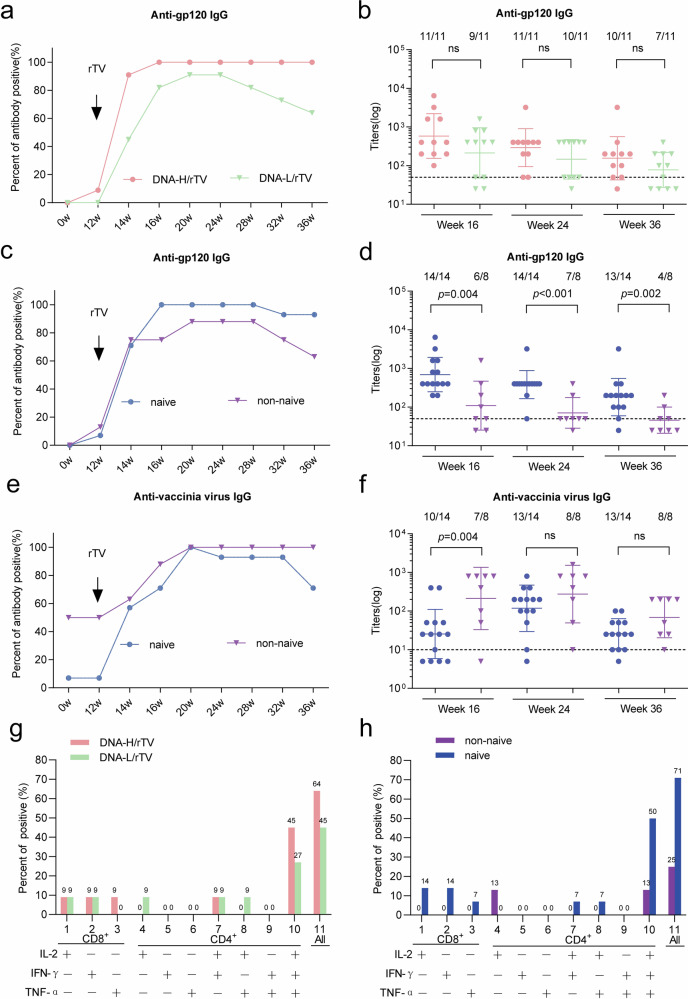
Humoral and cellular immune responses to HIV and vaccinia virus. **a** Response rates of anti-HIV gp120 IgG in DNA-L/rTV and DNA-H/rTV groups. **b** of Geometric mean titers (GMTs) of anti-HIV gp120 IgG in DNA-L/rTV and DNA-H/rTV groups, with error bars representing geometric standard deviations (GSD). **c** Response rates of anti-HIV gp120 IgG in vaccinia naïve and non-naïve subjects. **d** GMTs of anti-HIV gp120 IgG in vaccinia naïve and non-naïve subjects, with GSD error bars. **e** Response rates of anti-vaccinia virus IgG in vaccinia naïve and non-naïve subjects. **f** GMTs of anti-vaccinia virus IgG in vaccinia naïve and non-naïve subjects, with GSD error bars. **g** HIV-specific T cell responses rates of DNA-L/rTV and DNA-H/rTV groups. **h** HIV-specific T cell responses rates of vaccinia naïve and non-naïve subjects. ns: not significant (*P* > 0.05)

The DNA prime-rTV boost regimen induced anti-gp120 IgG across groups. Antibodies were evident at week 12, peaked at week 14 and 16 after rTV vaccination, and continued to the end of trial follow-up. Seroconversion occurred in 100% (11/11) of subjects in the DNA-H/rTV group by week 14 and 91% (10/11) of the DNA-L/rTV group by week 20. The geometric mean titers (GMT) of anti-gp120 IgG at week 16 were 618 in the DNA-H/rTV group and 222 in the DNA-L/rTV group; no significant differences were found between the two groups (Fig. 2a, b). However, higher response rates and magnitudes to gp120 antigens were observed in vaccinia-naïve subjects; all 14 vaccinia-naïve and 7 of 8 non-naïve subjects had positive responses with corresponding GMTs of 398 in the naïve group and 76 in the non-naïve group (*p* = 0.002, at week 24) (Fig. 2c, d). Quantitative correlation analyses demonstrated no significant association between baseline anti-vector antibody titers and peak anti-Env responses (Supplementary Fig. 1). No vaccine-induced HIV-specific antibody was observed in rTV-only groups (Supplementary Table 1). Neutralizing antibodies against 4 pseudoviruses were measured in TZM-bl cells 4 weeks after the final vaccination. No autologous or heterologous neutralization was detected.

IgG antibodies against vaccinia were detected in part of non-naïve subjects with titers of <100 at baseline. Vaccinia-specific IgG increased greatly after rTV vaccination both in vaccinia-naïve and non-naïve subjects. Seroconversion occurred in 100% (22/22) of subjects at week 20, and GMTs were consistently higher in the non-naïve group (ranging from 476 to 69) than in vaccinia-naïve group (ranging from 190 to 25), with a significant difference at week 16 (*p* = 0.004) (Fig. 2e, f).

Cellular immunogenicity to HIV antigens was assessed by intracellular cytokine staining assay (ICS). The total IFN-γ-, IL-2-, or TNF-α-secreting T-cell response rates for DNA-L/rTV and DNA-H/rTV groups were 45% and 64%, respectively (Fig. 2g). No differences were observed in the response rates between the two groups. CD8^+^ T-cell response rates were low, ranging from 0% to 9% (1/11). Vaccine-induced cellular responses were dominated by CD4^+^ T cell responses. Polyfunctional CD4^+^ T cells secreting two or three functional markers (IFN-γ, IL-2, or TNF-α) were detected in subjects receiving rTV boost. The positive rates of CD4^+^ T cells secreting IFN-γ, IL-2, and TNF-α were 27% for DNA-L/rTV and 45% for DNA-H/rTV group. HIV-specific CD4 + T-cell responses exhibited sustained durability, with no statistically significant decline in either response rates or magnitude observed between 2-week and 24-week post-final immunization (Supplementary Fig. 2). We observed that group DNA-H/rTV regimen had slightly higher TEM cell frequencies in the CD4 + T cell compartments at 2 weeks post final vaccination, while there was no difference in the frequency of CD4+ and CD8+ memory cells in any of the groups (Supplementary Fig. 3). Significantly higher HIV-specific T-cell response rates were observed in vaccinia-naïve subjects compared to non-naïve (total: 71% *vs*. 25%, *p* = 0.074) (Fig. 2h).

## Discussion

This study was designed to assess both the safety profile and immunogenic potential of a novel HIV vaccine regimen combining recombinant DNA vaccine priming with replication-competent vaccinia virus vaccine boosting, while concurrently examining the potential modulatory effects of preexisting anti-vaccinia immunity on the magnitude and quality of vaccine-elicited immune responses.

Overall, the HIV DNA and rTV vaccines, whether administered alone or in combination, were safe and well tolerated. Most of AEs were mild and transient. lymph node enlargement occurred in the armpit of the vaccinated side were common in subjects who received rTV vaccine, typically occurring within 2 weeks after rTV vaccination and resolving within 1–4 weeks. Above 90% of lymph node enlargement were reported by the vaccinia-naive subjects. The occurrence time, severity, and duration of lymph node enlargement after rTV inoculation were similar to smallpox vaccination.^18^ In a vaccinia-naive person who is not immunosuppressed, primary smallpox vaccination might cause swelling and tenderness of regional lymph nodes, beginning 3–10 days after vaccination and persisting for 2–4 weeks. Fever is one of the most common side effects after smallpox vaccination.^19^ In a smallpox vaccination trial involving 680 first-time vaccinees, 10% of subjects experienced fever, defined as a temperature above 37.7 °C within 14 days after vaccination.^20^ However, in our phase I trial, three subjects receiving rTV reported fever, with just one case probably related to rTV vaccination (<38 °C). rTV inactivates thymine nucleoside kinase (TK) by inserting the HIV antigen genes in the TK region, which may be the cause of its reduced local and systemic side effects.

We observed transient CK elevations in a small subset of rTV vaccine recipients. These elevations resolved spontaneously without medical intervention. Notably, CK-MB and troponin levels remained within normal limits across all assessments, and ECGs showed no clinically significant abnormalities. Although troponin monitoring was not implemented in phase Ia, subsequent phase Ib trials included serial troponin measurements at pre-vaccination and post-vaccination timepoints (days 3, 7, 14, 28, and 56). Consistent with the phase Ia findings, subjects in phase Ib demonstrated normal cardiac biomarkers (CK-MB and troponin) throughout the study period. This collective evidence effectively excludes myocardial injury as a potential cause of the observed CK elevations. A study assessed vaccinia-associated myocarditis in 540,824 military personnel vaccinated with a New York City Board of Health strain of vaccinia. Of these, 67 developed myopericarditis at 10.4 ± 3.6 days after vaccination; the ST-segment elevation was observed in 57%, and peak CK-MB was detected within 8 h of presentation.^21^ So, the possibility of elevated CK level caused by cardiomyopathy was excluded. In addition, ANA positive (1:160) was observed among a few subjects after DNA injection. No additional clinical manifestations associated with autoimmune disorder were identified in subjects who developed ANA positivity. Due to the small sample size of phase I trial, the relationship is not currently conclusive between rTV vaccination and elevated CK level and between DNA injection and ANA positive, which needed to be further determined in subsequent clinical trials.

After rTV vaccination, we documented the characteristic progression of cutaneous reactions at the inoculation site. This kinetic profile aligns with classical Type IV hypersensitivity mechanisms, demonstrating immunological engagement rather than incidental tissue damage. Most delayed-type hypersensitivity reactions are T-cell mediated. Activation of CD4+ T cells results in cytokine mediated inflammation which is typically confined to a local area. In reactions where CD8+ T cells are involved, the release of perforin and granzyme can lead to bystander cell injury and death by apoptosis. Delayed-type AE is one of the key vaccine safety monitoring. Their evaluation relies on long-term and systematic data collection and analysis. The AEs of rTV vaccine will be further evaluated through expanded cohort in subsequent trials.

Our study evaluated immune responses induced by the DNA prime-rTV boost regimen. Compared to vaccination with DNA, rTV boosting enhanced HIV-specific IgG antibody and T cell responses. DNA prime and replication-competent vaccinia boost regimen elicited durable polyfunctional CD4^+^ T cell and low rates of CD8^+^ T cell responses. CD4+ T helper cells play a critical role in facilitating antibody affinity maturation and are essential for priming CD8+ cytotoxic T cell responses. The DNA/rAd5 vaccine regimen (HVTN 505) did not reduce either the rate of HIV-1 infection or the viral-load set point although robust CD8+ T cell responses were elicited.^22^ The immune response characteristics of DNA-rTV are similar to those induced in RV144 trial, including high binding antibodies, multifunctional CD4+ response, and absence of neutralizing antibodies.^23^ While lacking the V1V2-specific IgG3 associated with partial efficacy observed in RV144 trial, DNA-rTV induces dominant polyfunctional CD4+ T-cell responses with sustained durability. In general, as a replicating viral vector, rTV offers unique advantages over non-replicating vectors: (1) prolonged antigen presentation through in vivo replication enhances germinal center activity, potentially driving broader antibody maturation; (2) innate immune activation via pathogen-associated molecular patterns (e.g., TLR2/6 ligands)^24^ may synergize with DNA priming to amplify adaptive responses; (3) capacity to induce more balanced humoral and cellular immune responses. Given these attributes advancing DNA-rTV to Phase II will enable critical evaluation of its potential efficacy while exploring replicating vectors as a tool for HIV vaccine.

Although it is often believed that pre-existing immunity to orthopoxvirus interferes with the immunogenicity of vaccinia-based vaccines, evidence for the influence of pre-existing immunity on recombinant vaccinia vaccination is ambiguous. Some studies showed that pre-existing anti-orthopoxvirus immunity had a negative effect on the induction of antigen-specific humoral and/or cellular immune responses by rMVA-based vaccines in mice and macaques.^25,26^ However, other studies showed that pre-existing anti-vector immunity did not interfere with rMVA-based vaccination.^27,28^ In addition, the results obtained in humans are also conflicting: multiple doses of rMVA vaccine enhance orthopoxvirus-specific immunity and have been shown to negatively affect the antigen-specific humoral and cellular immune responses.^29,30^ In this study, pre-existing anti-vaccinia IgG were detected in half of non-naïve subjects at baseline; higher HIV IgG titers (week 24) and polyfunctional CD4^+^ T cell responses (week 16) were observed in naïve subjects. Vaccinia-specific immunity prior to vaccination seems to have a little influence on the induced humoral and cellular responses. Therefore, the question of whether pre-existing vector immunity interferes with the immunogenicity of recombinant vaccines still requires further research.

This study has some limitations. First, it included a relatively small sample size (*n* = 48), missing some potentially valuable information. Second, the pseudoviruses used in the evaluation were not perfectly matched to the sequence of Env gene expressed in the vaccine. The pseudoviruses CH120 (Tier 3) and CH181.12 (Tier 2) are both unmatched and having high neutralization resistance. The Tier 1 pseudoviruses (SVPC5, SAPC12), while sensitive to neutralization, are not sequence-matched to our vaccine strain HIV-1 CN54. The trade-off between subtype matching and assay sensitivity represents a recognized limitation in our study. Furthermore, longer-term safety and immunogenicity might need to give a better understanding of the safety and longevity of the replication-competent recombinant vaccinia vaccine. Nevertheless, our trial provided information on the reactogenicity and immunogenicity of the candidate vaccine based on replicating vaccinia Tiantan.

In conclusion, the heterologous prime-boost regimen combining DNA with replication-competent Tiantan vaccinia vectors demonstrated favorable safety and tolerability profiles while eliciting robust humoral and cellular immunity against HIV antigens. These findings collectively substantiate advancing this vaccine strategy to Phase II clinical evaluation, where comprehensive assessments should focus not only on further evaluating safety and immunogenicity but also on exploring potential correlations between immune parameters and clinical protective endpoints. The observed antigen-specific immune activation patterns, particularly the balanced humoral/ cellular profile and durable memory responses, underscore the translational potential of this DNA and replicating poxvirus vectors based prime-boost approach in HIV vaccine development.

## Materials and methods

### Study design

This was a randomized, double-blind, placebo-controlled phase I clinical trial. The trial was registered at Chinese Clinical Trial Registry (Registration NO.: ChiCTR-PRC-10001287), and conducted at Peking Union Medical College Hospital in China. The protocol was approved by the ethics committees of Peking Union Medical College Hospital and National Center for AIDS/STD Control and Prevention, China CDC. All procedures performed in this study involving human subjects were in accordance with the ethical standards of the institutional and/or national research committee and with the 1964 Helsinki declaration and its later amendments or comparable ethical standards. This study was explained to all subjects in detail by the site researchers, and all subjects signed written informed consent documents.

### Subjects and randomization

HIV-negative healthy adults (18–55 years old) at low risk of HIV-1 infection, with or without a history of smallpox vaccination, were enrolled. Potential subjects met eligibility criteria based on medical history, physical exam, and laboratory tests. Exclusion criteria included pregnancy, nursing, inherited or acquired immunodeficiency disease, eczema, atopic dermatitis, or any diseases causing skin damage, and close contact with pregnant women, immunosuppressed persons, persons with eczema, or infants less than 12 months of age,^20^ and other medical conditions determined by clinical researchers to be unsuitable for enrollment. Female subjects were asked to practice effective birth control and avoid pregnancy until 6 months after the last vaccination.

This clinical trial included two stages, phase Ia (*n* = 12) and Ib (*n* = 36). For the safety considerations, randomization was not used in phase Ia. At first, six subjects received a single dose of rTV. After confirming the safety of the vaccination, other six subjects in the rTV-H group received simultaneous unilateral administrations of two dose rTV in the deltoid region, with vaccination sites separated by ≥2 cm. In phase Ib, 36 subjects were randomized to receive DNA (4 mg) alone (*n* = 6), DNA (4 mg) /rTV (*n* = 12), DNA (2 mg) /rTV (*n* = 12), or placebo (*n* = 6). Randomization was done by the Division of Health Statistics, China CDC, using software SAS 9.1.2. Due to the obvious cutaneous reactions after rTV vaccination, the blind was invalid for site researchers 3 days after rTV vaccination. Though laboratory personnel responsible for endpoint assays were blinded as to treatment assignments during the conduct of the trial.

### Interventions

The DNA vaccine consisted of two plasmids, pGP140 and pGPNEF, expressing gp140 and Gag-Pol-Nef proteins derived from HIV-1 strain CRF07_BC CN54, respectively. Gag-Pol-Nef was a fusion protein and had been modified to remove undesirable domains for safety consideration.^31^ In both plasmids, the codon usage of HIV genes was humanized optimized to enhance its immunogenicity. The DNA vaccine was formulated with 20 mM phosphate buffer containing 0.9% sodium chloride, pH 7.2. DNA placebo was sodium chloride, 0.9%. rTV vaccine was a replication-competent recombinant virus based on vaccinia virus Tiantan, expressing HIV-1 CN54 gp140, gag, and part of pol proteins. rTV was grown in chicken embryo fibroblasts and formulated with protectant (80% glycerol containing 6.6% proteose peptone and 2% starch). DNA and rTV placebos were 0.9% sodium chloride and protectant, respectively.

Phase Ia aimed to assess the safety of rTV vaccination. rTV was administered to the deltoid area via scarification by 15 punctures with a bifurcated needle. Six subjects in rTV-L group were given a single dose of rTV vaccine, while in rTV-H group, the subjects received two doses of rTV vaccine unilaterally, with a distance of more than 2 cm between vaccination sites. After vaccination, the sites were covered with a semipermeable dressing to avoid autoinoculation or exposure of personal contacts to vaccinia virus, as described previously.^32^

In phase Ib, eligible subjects received DNA vaccine alone, DNA and rTV vaccines, or placebos according to assignment. Two DNA prime-rTV boost groups of subjects were injected intramuscularly with 2 mg (DNA-L/rTV group) or 4 mg (DNA-H/rTV group) of DNA vaccine in the deltoid muscle of the bilateral upper arms at weeks 0, 4, and 8. At week 12, both groups received two doses of rTV unilaterally administered via bifurcated needle in the deltoid region, with vaccination sites spaced ≥2 cm apart. DNA group of subjects were injected with 4 mg DNA vaccine at weeks 0, 4, and 8, and received rTV placebo at week 12 with a bifurcated needle. Placebo group of subjects received DNA placebo at weeks 0, 4, and 8 and rTV placebo at week 12. Subjects were followed up for 6 months after the final vaccination for clinical evaluation and laboratory testing.

### Objectives

The primary objectives of the phase I trial were to assess the safety and tolerability of rTV alone, DNA alone, and DNA prime-rTV boost vaccination regimen. The secondary objective was the assessment of the immunogenicity of DNA in combination with rTV.

### Safety evaluation

Safety assessments were performed on all subjects. Given that local reactions following rTV vaccination typically persist for ~4 weeks, this trial extended the observation period for reactogenicity to 28 days post vaccination, including local reactions at the administration site (pain, itch, redness, swelling) and systemic events (fever, chills, lymph nodes, headache, malaise and/or fatigue, myalgia, arthralgia, nausea, vomiting, and rash) rTV vaccine-related skin lesions (papule, vesicle, ulcer, and pustule) also needed to be observed. rTV vaccination success was measured by the development of a “take” at the site of inoculation, defined as the presence of a major cutaneous reaction (a vesicular or pustular lesion) 7–14 days postvaccination.^33^ All AEs and SAEs during the trial were recorded and graded by the investigators. Criteria for grading clinical and laboratory events were based on the Guidance for Adverse Reaction Grading Scale for Preventive Vaccine Clinical Trial (National Medical Products Administration, P.R. China) and other international guidance. AEs were assessed for their relationship to study vaccines by the investigators. After the completion of the acute observation of phase Ia, an independent data and safety monitor board reviewed the AEs to ensure subjects safety and agreed to apply for phase Ib trial.

### Immunogenicity analysis

Vaccine-induced binding antibodies were analyzed using HIV-1 clade CRF07_BC-specific enzyme-linked immunosorbent assay (ELISA). The pre-existing and vaccine-induced anti-vaccinia IgG were assessed by ELISA with whole inactivated vaccinia viruses. The 96-well plates were coated with 1 μg/ml CN54 gp120 protein or 10^7^ PFU/ml vaccinia virus Tiantan. HIV-1 neutralizing antibody titers were measured as the reduction in luciferase gene expression after a single viral infection in TZM-bl cells, as described previously.^17^ Serum samples were three-fold serially diluted starting from 1:20 and tested against four HIV-1 Env-pseudotyped viruses, including CH120, CH181.12, SVPC5, and SAPC12. The 50% of inhibitory dilution (ID_50_) neutralization titers were calculated for each sample.

ICS was performed on cryopreserved PBMCs at weeks 0, 14, 20, and 36 by flow cytometry to determine HIV-specific CD4^+^ and CD8^+^ T cell responses. Pre-thawed PBMCs were cultured overnight and then stimulated with peptide pools (2 μg of each single peptide) in 100 μl of complete media (RPMI 1640 plus 10% fetal bovine serum). All peptides (Synthesized by CL. BIO-Science Co., LTD) used in this study were 15mers with 11 overlapping and encompassed the HIV-1 Gag, Env, Pol, and Nef regions from CN54. For functional analyses, the following fluorochrome-conjugated antibodies (BD Bioscience) were used: CD3-Pacific Orange, CD4-PE-Texas Red, CD8-Pacific Blue, IL-2-PE, IFN-γ-Alexa 700, TNF-α-FITC, CCR7-PE-Cy7, CD45RA-APC, CD27-APC-Cy7, and Vivid-Live-dead blue. The frequency and magnitude of CD4^+^ and CD8^+^ T-cells producing IFN-γ, IL-2, or TNF-α in response to HIV-1 peptide pools were reported. The polyfunctional T cell response was evaluated by the percent of CD4^+^ and CD8^+^ T-cells producing two or more cytokines.

### Statistical analysis

The primary outcomes of the study related to safety, and the secondary outcomes related to HIV-specific antibody responses, T cell responses, and vaccinia virus-specific antibodies. The T cell responses were analyzed as positive or negative and reported as the number and proportion of subjects responding to HIV-1 proteins. An ICS response was considered positive if the percentages of the cytokine-secreting cells in the stimulated samples were three times more than the value of the unstimulated controls and higher than 0.02% after background subtracted. Each subject was classified as a responder if there was at least one positive response against any of the HIV-1 peptide pools at any time in the ICS assay. For the ELISA assay, Wilcoxon Signed-Rank Test was used to determine whether there is or is not a significant difference in the titer of antibodies between DNA-H/rTV and DNA-L/rTV groups, vaccinia-naïve and non-naïve groups. Spearman correlation analyses compared baseline anti-vector antibody titers with peak anti-Env binding antibody responses.

## Supplementary information

Supplementary materials

Supplementary Table 1

Protocol 1

Protocol 2

IRB approvals

## Data Availability

All data generated or analyzed during this study are included in this article. Any request for raw data sharing should be sent to yshao@bjmu.edu.cn, and will be reviewed and licensed on the basis of scientific merit by the sponsor, investigators and all collaborators.
