# Supplementary material for: Safety and immunogenicity of an HIV vaccine trial with DNA prime and replicating vaccinia boost
Source: Signal Transduct Target Ther. 2025 Jul 2;10:208. doi: 10.1038/s41392-025-02259-y (PMC12217030; doi:10.1038/s41392-025-02259-y)
Supplement: Supplementary file 1 — Supplementary materials [file 41392_2025_2259_MOESM1_ESM.docx]

Supplementary Materials for

**Safety and immunogenicity of a phase I HIV vaccine trial with DNA vaccine prime and replication-competent recombinant vaccinia virus boost**

Ying Liu^1§^, Wei Lv^3§^, Pu Shan^2^, Dan Li^1^, Ying-Qi Wu^1^, You-Chun Wang^4^, Yuan-Yuan Li^2^, Qiang Liu^4^, Jian-Sheng Wang^5^, Yan-Ling Hao^1^, Yong Liu^1^, Wei-Jin Huang^4^, Li Ren^1^, Shu-Hui Wang^1^, Tai-Sheng Li^3*^, Jing Xu^2*^, Yi-Ming Shao^1*^

Correspondences to: yshao@bjmu.edu.cn

**This PDF file includes:**

**Supplementary figures. S1 to S3**

**Other Supplementary Materials for this manuscript include the following:**

**Ia study protocol**

**Ib study protocol**

**IRB approval**

**Table S1. Immune response data of rTV-only and DNA-only groups**

**Supplementary figure 1.**


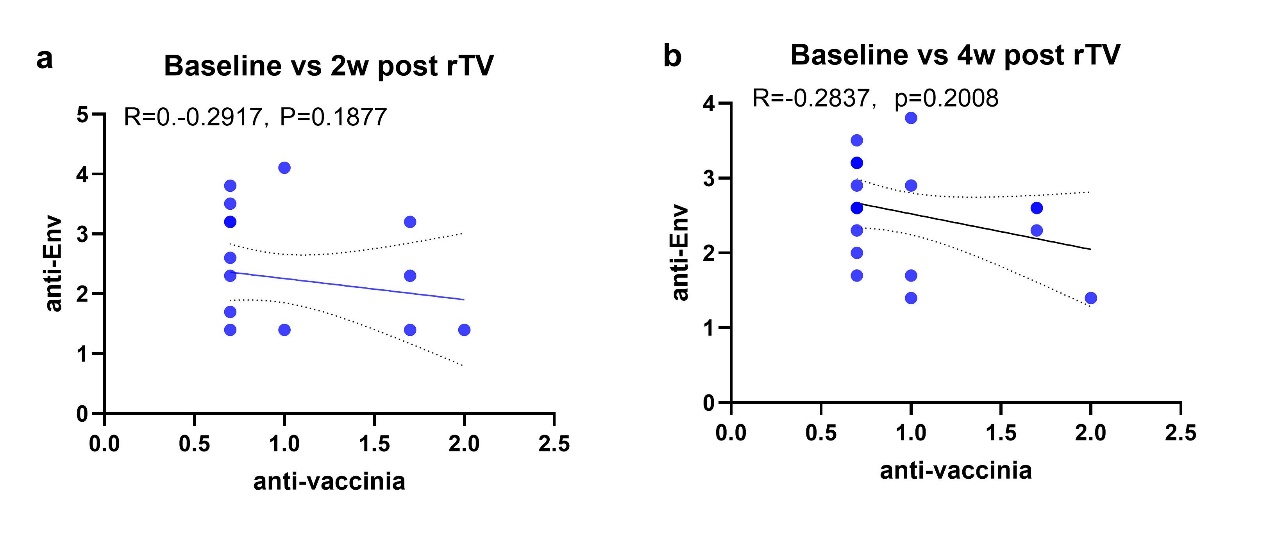


**Supplementary figure 1. Spearman correlation analyses comparing anti-vaccinia antibodies with anti-Env binding antibodies.** a. OD values of antibody against vaccinia virus at baseline vs titers of Env-specific antibody at week 2 post rTV; b. OD values of antibody against vaccinia virus at baseline vs titers of Env-specific antibody at week 4 post rTV.

**Supplementary figure 2.**

**
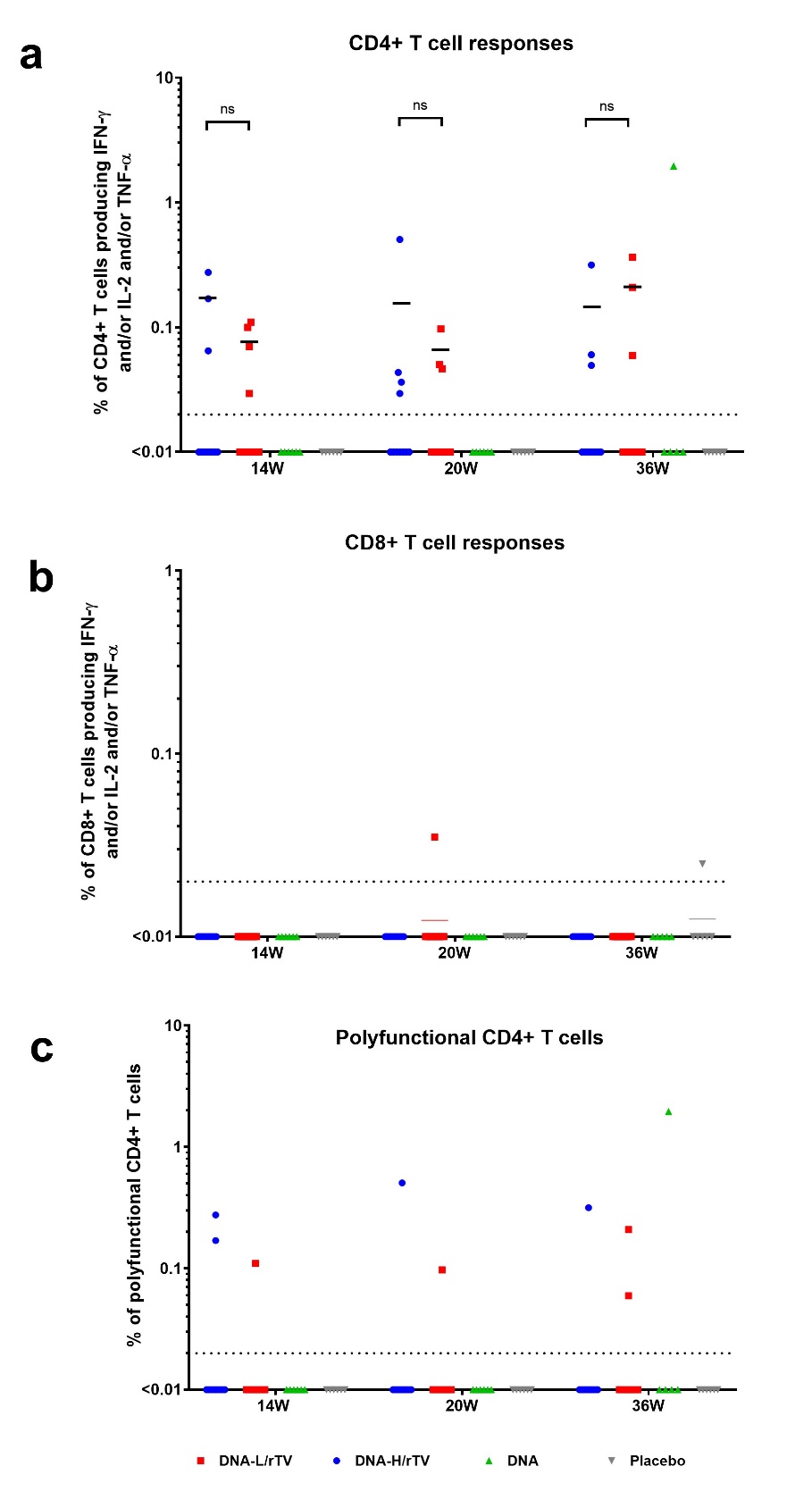
**

**Supplementary figure 2. The Magnitude of HIV-specific IFN-γ/IL-2/TNF T cell responses.** The percentages of total CD4+ T cells (a) and CD8+ T cells (b), and polyfunctional CD4+ T cells (c) expressing IFN-γ, IL-2 or IFN-γ in response to Env and Gag peptide pools are shown. Results are collected at study week 14, 20 and 36, which are from 2, 8, and 24 weeks after the final vaccination. The line indicates the mean of the frequency in positive responders only.

**Supplementary figure 3.**


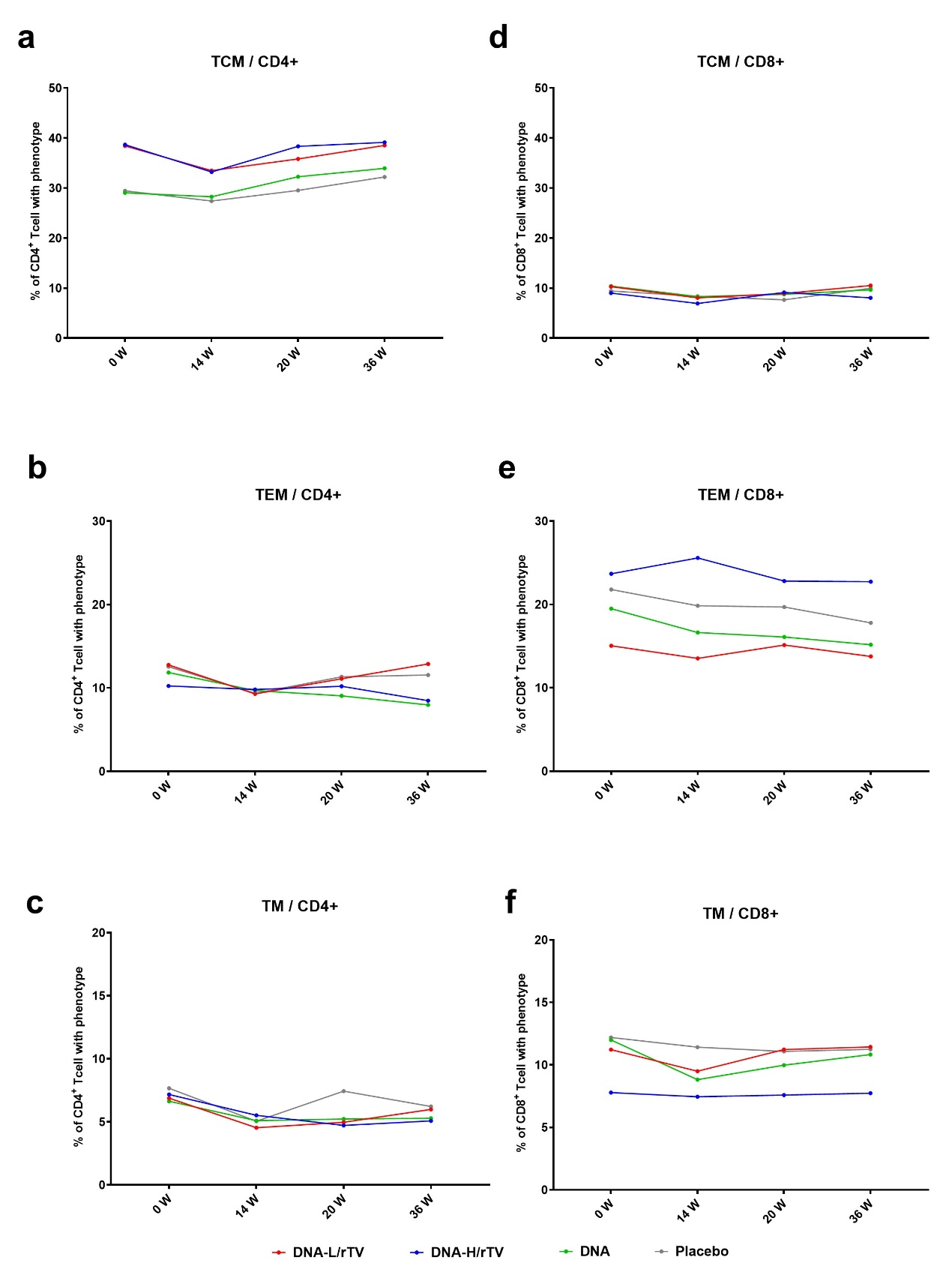


**Supplementary figure 3. Phenotype of vaccine-induced memory T cell responses.** Frequency (%) of total CD4+ (a-c) and CD8+ (d-f) memory T cell subsets in Placebo, and vaccine groups at Pre-vaccination, two weeks (week 14), 8 weeks (week 20) and 24 weeks (week 36) post last vaccination. The superimposed dot plots summarize the % frequency of total vaccine-induced CD8+ memory T cell subsets.
