# Supplementary material for: Safety and immunogenicity of an HIV vaccine trial with DNA prime and replicating vaccinia boost
Source: Signal Transduct Target Ther. 2025 Jul 2;10:208. doi: 10.1038/s41392-025-02259-y (PMC12217030; doi:10.1038/s41392-025-02259-y)
Supplement: Supplementary file 2 — Supplementary Table 1 [file 41392_2025_2259_MOESM2_ESM.docx]

**Table S1. Immune response rates of rTV-only and DNA-only groups**

|  | Group | Time post final vaccination | | |
| --- | --- | --- | --- | --- |
|  |  | Week 2 | Week 8 | Week 24 |
| ICS | rTV-H | 2/6 | 5/6 | 0/6 |
|  | rTV-L | 1/6 | 4/6 | 1/6 |
|  | DNA | 1/6 | 2/6 | 1/6 |
| Anti-HIV | rTV-H | 0/6 | 0/6 | 0/6 |
|  | rTV-L | 0/6 | 0/6 | 0/6 |
|  | DNA | 1/6 | 0/6 | 0/6 |
