# Supplementary material for: Safety and immunogenicity of an HIV vaccine trial with DNA prime and replicating vaccinia boost
Source: Signal Transduct Target Ther. 2025 Jul 2;10:208. doi: 10.1038/s41392-025-02259-y (PMC12217030; doi:10.1038/s41392-025-02259-y)
Supplement: Supplementary file 3 — Protocol 1 [file 41392_2025_2259_MOESM3_ESM.pdf]

AIDS Vaccine (Nucleic Acid in Combination with Recombinant Vaccinia Tiantan)

Phase Ia Clinical Trial Protocol

Approval number: 2006L04176

Vaccine Development Units:

National Center for AIDS/STD Control and Prevention, Chinese Center for Disease Control and Prevention

Beijing Institute of Biological Products

Clinical trial site：

Peking Union Medical College Hospital, Chinese Academy of Medical Sciences

Statement of Project Leader

I have read and agreed with this study protocol and will conduct this clinical study in accordance with the design and requirements in this protocol. The changes in this study protocol will not be made unless such changes have been agreed by both parties, and this study can be implemented only after it has been approved by the Ethics Committee.

National Center for AIDS/STD Control and Prevention, Chinese Center for Disease Control and Prevention

Professor Yiming Shao

________/_______/_______

YYYY/MM/DD

Peking Union Medical College Hospital, Chinese Academy of Medical Sciences

Professor Taisheng Li

________/_______/_______

YYYY/MM/DD

Table of Contents

Synopsis of Clinical Trial Protocol 1
Table of Abbreviations 5
1 Introduction 7
1.1 Current Status of the HIV/AIDS Epidemic 7
1.2 Molecular epidemiology of HIV 8
1.3 Methods for HIV / AIDS prevention and control 8
1.4 Overview of AIDS Vaccine Research 9
2 Study Product 11
2.1 DNA Vaccine 11
2.1.1 Current Status of AIDS DNA Vaccine Research 11
2.1.2 HIV1 DNA Vaccine Vector 12
2.1.3 HIV1 DNA Vaccine 12
2.2 Recombinant Vaccinia Virus Vector Vaccine 12
2.2.1 Research of Replicating Vaccinia Virus Vector Vaccine 12
2.2.2 Recombinant Vaccinia Virus Vaccine 13
2.3 Summary of Preclinical Trials 14
2.3.1 Safety Evaluation in Animal 14
2.3.2 Immunogenicity Study in Animal 14
2.4 Product Package, Doses and Method of Vaccination 15
3 Objectives and Rationale for the Study 16
4 Research Institutions and their Responsibilities 17
4.1 Responsibilities of Sponsor(s) 17
4.2 Responsibilities of Investigator 19
4.3 Responsibilities of the Data Management Center 20
5 Phase Ia protocol 21
5.1 Objectives 21
5.2 Endpoint Events 21
5.2.1 Primary Endpoint Events 21
5.2.2 Secondary Endpoint Events 22
5.3 Recruitment of Study Subjects 22
5.4 Protocol 23
5.4.1 Inclusion and Exclusion Criteria 24
5.4.2 Immunization and Followup Plan 26
5.4.3 Determination of Success of rTV Vaccination and Revaccination 33
5.5 Vaccine Safety Evaluation 33
5.5.1 Evaluation of Adverse Events and Their Relationship to the Investigational Vaccine 34
5.5.2 Reporting of Adverse Events 37
5.5.3 Management of Adverse Events 37
5.5.4 Immunogenicity Evaluation 38

5.5.5 Early Termination of the Trial......39
5.6 HIVPositive Related Issues and Management During the Trial......40
5.6.1 HIV Antibody Positivity Induced by Vaccination......40
5.6.2 HIV Infection During the Trial......40
5.7 Data Management......41
5.7.1 Data Management System......41
5.7.2 Completion and Modification of CRF Forms......41
5.7.3 Data Locking......42
5.8 Statistical Analysis......42
5.8.1 Study Indicators......42
5.8.2 Sample Size......42
5.8.3 Randomization of Subjects......42
5.8.4 Blinding Design......43
5.8.5 Statistical Analysis Content and Methods......43
5.8.6 Hypothesis Testing and Significance Level......44
5.8.7 Handling of Missing Data......44
5.8.8 Statistical Analysis Software......44
5.9 Data Management......44
5.9.1 Data Management at Clinical Trial Sites......44
5.9.2 Data Management by the Sponsor......44
5.9.3 Ownership of Trial Data......45
5.10 Confidentiality and Ethics......45
6 Quality Assurance and Quality Control in Clinical Trials......45
6.1 Data Safety Monitoring Board (DSMB)......45
6.2 Quality Assurance in Clinical Trials......46
6.3 Clinical Trial Monitoring......46
6.4 Clinical Trial Audits and Inspections......46
Annex 1. Informed Consent Form for Participation in a Phase Ia Clinical Trial of AIDS vaccine (Nucleic Acid in Combination with Recombinant Vaccinia Tiantan)......48
Annex 2. Grading of Clinical and Laboratory Adverse Events......62
Annex 3. Subject Diary Card......67
Annex 4. Summary of Test Results for HIV1 DNA Vaccine......70
Annex 5. Summary of Test Results of Recombinant Tiantan Vaccinia (rTV) AIDS Vaccine.72
Annex 6： Reactions after Smallpox Vaccination and Principles of Treatment......74
Annex 7. Documents Stored for the Clinical Trial......81
Annex 8. Serious Adverse Events (SAEs) Report Form......83
References......85

Synopsis of Clinical Trial Protocol

The AIDS vaccine (Nucleic Acid in Combination with Recombinant Vaccinia Tiantan) has been jointly developed by the National Center for AIDS/STD Control and Prevention, Chinese Center for Disease Control and Prevention and the Beijing Institute of Biological Products. It consists of two components: the HIV-1 DNA vaccine and the recombinant vaccinia virus (rTV) vaccine. In accordance with SFDA requirements, this Phase I clinical trial protocol is designed to proceed in a stepwise manner, divided into two stages: Ia and Ib. The phase Ia study will observe the safety and immunogenicity of the rTV vaccine alone in 12 subjects. Upon completion of the acute reaction observation period in the phase Ia, safety data will be summarized and reported to the SFDA. Following SFDA approval, the phase Ib will commence. The trials will require a total of 48 subjects, with an expected total study duration of approximately 58 weeks.

The Phase Ia study will involve 12 subjects, including those vaccinated and unvaccinated with smallpox vaccine. The study duration is 26 weeks. The primary objective is to evaluate the safety and tolerability of a single vaccination with rTV vaccine, administered at either one dose or two doses by bifurcated needle, in healthy volunteers with or without prior smallpox vaccination. Additionally, the study will preliminarily assess the vaccine's immunogenicity. The Phase Ia trial is divided into two stages based on the occurrence of reactions to the rTV vaccine vector—the TianTan strain of vaccinia virus. These stages are the acute reaction observation phase following vaccination (weeks 0-8) and the convalescent reaction observation phase (weeks 9-26). During the acute reaction observation phase, local and systemic adverse reactions occurring within the typical post-vaccination reaction period and the subsequent 3-5 weeks will be monitored. Upon completion of this phase, the trial will proceed to the convalescent phase, focusing on the observation of safety and immunogenicity post-vaccination. This includes assessing HIV antigen-specific immune responses in subjects and continuing to monitor all indicators from the first phase.

Phase Ib trial will involve 36 subjects, including 18 individuals who have previously been vaccinated against smallpox and 18 who have not. The study duration is 38 weeks. The primary objective is to evaluate the safety and tolerability of the immunization regimen with 2 mg or 4mg DNA vaccination for three times in combination with rTV vaccination for once in healthy volunteers who have previously been vaccinated against smallpox and those who have not. Additionally, the study will preliminarily assess the immunogenicity of this regimen.

Subjects will be randomly assigned to either the experimental groups or the control group. Subjects in the experimental groups will receive three doses of the DNA vaccine (2 mg or 4 mg) at 4-week intervals, followed by one dose of the rTV vaccine at the highest safe dose determined during Phase Ia. Subjects in the control group will receive placebos of DNA vaccine or rTV vaccine at the same time points and in the same volumes as the experimental groups. Following each vaccination, participants will be closely monitored for systemic and local reactions. After rTV vaccination, safety and HIV antigen-specific immune responses will be observed for at least 24 weeks.

This protocol focuses on the research protocol for the Phase Ia clinical trial.

Timeline for Phase I Clinical Trial:

**Total trial duration (weeks)**	**-4 ~-1**	**0**	**1 ~6**	**7～8**	**9～16**	**17 ~20**	**21**	**22 ~24**	**25**	**26～28**	**29**	**30～32**	**33**	**34 ~57**
Ia-recruitment														
Ia-administration		rTV												
Ia-acute reaction observation phase														
Ia-summary and reporting of the acute reaction observation phase to the SFDA									**End of Phase Ia**					
Ia-recovery reaction observation phase														
Obtained SFDA approval to conduct Phase Ib trial														
Ib-preparation														
Ib-recruitment														

Ib-administration							DNA/ placebo		DNA/ placeb o		DNA/ placebo		rTV/ placebo		
Ib-safety and immunogenicity observation															

Table of Abbreviations

Abbreviations	Full Term
**AE**	Adverse Event
**ALVAC**	Avipoxvirus Canarypox
**ALT**	Alanine Transaminase
**AST**	Aspartate Aminotransferase
**AIDS**	Acquired Immunodeficiency Syndrome
**BLN**	Blood Urea Nitrogen
**CTL**	Cytotoxic T Lymphocyte
**CRF**	Case Report Form
**DMP**	Data Management Plan
**DRQ**	Data Query
**DSMC**	Data and Safety Monitoring Committees
**ELISA**	Enzyme-Linked Immunosorbent Assay
**ELISPOT**	Enzyme-Linked Immunospot Assay
**GCP**	Good Clinical Practice
**GMP**	Good Manufacturing Practice
**HIV**	Human Immunodeficiency Virus
**HIVAC-1e**	Recombinant Vaccinia Virus Vaccine Expressing HIV-1 Envelope Glycoprotein
**HLA**	Human Leukocyte Antigen
**HVTN**	HIV Vaccine Trials Network
**IC**	Informed Consent
**ICF**	Informed Consent Form

**IDU**	Idoxuridine
**IRB**	Institutional Review Board
**MVA**	Modified Vaccinia Virus Ankara
**NCAIDS**	National Center for AIDS/STD Control and Prevention，China CDC
**NIFDC**	National Institutes for Food and Drug Control
**NIH**	National Institutes of Health
**NYVAC**	Copenhagen Strain of Vaccinia Virus
**PBMC**	Peripheral Blood Mononuclear Cell
**PCR**	Polymerase Chain Reaction
**PUMCH**	Peking Union Medical College Hospital
**QA**	Quality Assurance
**QC**	Quality Control
**RPR**	Rapid Plasma Reagin test
**rTV**	Recombinant Tiantan Vaccinia
**SAE**	Serious Adverse Event
**SAP**	Statatistical Analysis Plan
**SFDA**	State Food and Drug Administration
**SHIV**	Simian-human Immunodeficiency Virus
**SIV**	Simian Immunodeficiency Virus
**SJS**	Stevens-Johnson Syndrome
**SMP**	Study Monitoring Plan
**TPHA**	Treponema Pallidium Hemaggulination Test
**ULN**	Upper Limit of Normal

1 Introduction

1.1 Current Status of the HIV/AIDS Epidemic

Acquired Immunodeficiency Syndrome (AIDS) is an infectious disease caused by the Human Immunodeficiency Virus (HIV). Since the first case was identified in 1981, HIV has been spreading globally at an alarming rate, becoming one of the most severe viral diseases threatening human life and health. Despite the annual increase in global funding for HIV/AIDS prevention and control and the widespread promotion of HIV infection drug therapies, the epidemic trend of HIV/AIDS has still exceeded expectations. By the end of 2006, the number of people living with HIV worldwide had reached 39.5 million. In 2006 alone, there were approximately 4.3 million new HIV infections and about 2.9 million deaths due to AIDS[1]. In China, the HIV epidemic has transitioned from a sporadic phase to a localized epidemic phase, and by the late 1990s, it had entered a rapid growth phase. As of January 2006, there were approximately 650,000 HIV-infected individuals in China, including about 75,000 AIDS patients. In 2005 alone, there were 70,000 new HIV infections and 25,000 deaths due to AIDS in China[2]. Without effective prevention and treatment methods, the number of HIV-infected individuals in China could reach 10 million by 2010.

The HIV epidemic has had a profound impact on the social and economic development of the world. In some developing countries, HIV infection has led to a reduction in life expectancy, a decrease in the labor force, and food shortages, setting back economic and social development by 20 years. Since the majority of HIV-infected individuals are young adults, the loss of labor capacity directly results in reduced household income, while the treatment of AIDS increases household expenditures. According to statistics, household incomes in South Africa and Zambia have decreased by 66-80% due to HIV infection, and the economies of these countries have consequently suffered significant damage.

Since the first imported case of AIDS was identified in 1985, the HIV epidemic in China has gone through three phases: the introduction phase (1985-1988), the dissemination phase (1989-1994), and the growth phase (1995 to present). Currently, the trend of HIV prevalence in China is markedly increasing. The epidemic among high-risk groups has not been effectively controlled and is beginning to spread to the general population, with the peak period of AIDS incidence and mortality already occurring in some areas. Moreover, due to the widespread presence of risk factors for the AIDS epidemic, there is a significant potential for an expanded epidemic in China. Therefore, the threat posed by the HIV epidemic to the

lives and health of the Chinese people is severe, and it is having a significant impact on the social and economic development of China, as well as Asia and the world at large[3].

1.2 Molecular epidemiology of HIV

Through phylogenetic analysis of HIV strains, it has been determined that HIV can be classified into distinct genetic subtypes. The HIV subtype B is predominantly prevalent among men who have sex with men and intravenous drug users in Europe and America. However, in regions such as sub-Saharan Africa, India, and China, subtype B constitutes only a minor portion of HIV infections, with a significant proportion of infections being attributed to subtype C or B/C recombinant forms primarily based on subtype C. Since the 1990s, the National Center for AIDS/STD Control and Prevention (NCAIDS) of the Chinese Center for Disease Control and Prevention has conducted two large-scale national molecular epidemiological surveys of HIV. These surveys identified eight types of HIV-1 and HIV-2 strains across thirty provinces, municipalities, and autonomous regions, including subtypes A, B (Western B), B' (Thai B), C, D, E, F, and G. Among these, over 80% were HIV-1 strains of subtypes B' and C. Further research revealed that subtypes B' and C of HIV-1 have recombined within China, and this B'/C recombinant virus may have gained a transmission advantage, potentially accelerating the HIV epidemic. The proportion of infections caused by the B'/C recombinant strain has been increasing annually among all infected individuals. In 1996, the recombinant virus was detected in only four provinces and autonomous regions (Xinjiang, Sichuan, Yunnan, and Shanghai). By 2000, it had spread to ten provinces, municipalities, and autonomous regions, including Xinjiang, Sichuan, Zhejiang, Gansu, Ningxia, Yunnan, Shanghai, Chongqing, Fujian, and Guangdong. By the end of 2002, the recombinant virus was found in most parts of the country, with the infection proportion rising from 30.39% in 1996 to 50.20%. Given the increasing proportion of the B'/C recombinant strain among infections in China and the apparent transmission advantage of this strain, selecting the major antigenic genes of the B'/C recombinant strain as immunogens for a vaccine would be beneficial for rapidly curbing the spread of AIDS in China.

1.3 Methods for HIV / AIDS prevention and control

Due to the high cost of antiretroviral therapy (ART) drugs, it is currently impractical to implement large-scale anti-HIV drug treatments in HIV/AIDS prevalent areas, especially in developing countries. More importantly, there is no drug available that can completely eradicate HIV infection. The emergence of drug-resistant strains means that these

medications are not effective for every patient, and the complexity of the medication regimen leads to some patients failing to adhere to the treatment schedule, resulting in treatment failure. Therefore, there is an urgent need to explore safe and effective methods to prevent and control the spread of HIV/AIDS.

The prevention and control of HIV/AIDS primarily involve two aspects: firstly, implementing educational campaigns and behavioral interventions, and secondly, developing vaccines for widespread vaccination among susceptible populations. Only by adopting a dual strategy of behavior intervention, primarily through education and awareness campaigns, and biomedical intervention, primarily through vaccines, can we ultimately control HIV/AIDS globally. Abandoning or neglecting either strategy will delay the control of the epidemic. Historical experience has proven that the most cost-effective method to control epidemic diseases is the application of vaccines, as seen in successful cases such as smallpox, polio, measles, and hepatitis. Therefore, the development of a safe and effective AIDS vaccine has always been a goal for scientists. The Chinese government places great importance on the prevention and control of HIV/AIDS. In 1998, it released the "Medium and Long-Term Plan for the Prevention and Control of AIDS in China," setting the goal of developing an AIDS vaccine targeting the prevalent strains in China and completing clinical trials by 2010.

1.4 Overview of AIDS Vaccine Research

The research on HIV/AIDS vaccines can be divided into four stages: The first stage (1980s) was the initial phase of AIDS vaccine research, characterized mainly by single protein subunit vaccines aimed at inducing neutralizing antibodies, while overlooking the role of cellular immunity. The second stage (1990s) was marked by an overemphasis on cellular immunity and a neglect of the role of neutralizing antibodies. The vaccine forms during this stage were predominantly recombinant viral vector vaccines. The third stage (2000-2005) focused on balancing the humoral and cellular immune responses induced by vaccines, with comprehensive development of DNA vaccines, live vector vaccines, and multivalent protein vaccines, emphasizing combined immunization with various types of vaccines, significantly accelerating the pace of vaccine clinical trials. The fourth stage (2005-present) has learned from the lessons of the previous three stages, with vaccine design paying more attention to antigen modification and the replicability of vectors to induce stronger humoral and cellular immune responses.

AIDS candidate vaccines include traditional vaccines (inactivated vaccines and live

attenuated vaccines), synthetic peptide and protein subunit vaccines, DNA vaccines, and live vector vaccines. The basic research status of these vaccine forms is as follows: (1) Due to safety concerns, researchers are cautious about the application of live attenuated vaccines in humans[4-6]. (2) Inactivated vaccines cannot establish virus-specific protective immunity in monkeys. (3) Synthetic peptide vaccines have a poor ability to stimulate the body to produce neutralizing antibodies and cellular immunity[7-9]. Subunit vaccines, such as highly purified recombinant monomeric HIV-1 envelope proteins, do not elicit virus-specific cytotoxic T lymphocyte (CTL) responses even when effective immune activators are used, and the antibodies produced cannot neutralize HIV-1 primary isolates. Recently, VaxGen in the United States has just concluded Phase III clinical trials of an AIDS subunit vaccine in the United States and Thailand, with very disappointing results, as no protective effect of the vaccine was observed[10]. (4) DNA vaccines have shown good application prospects due to their ability to induce effective cellular and humoral immune responses, good safety, low production and transportation costs, good technical versatility, and short development cycles[11]. (5) Live vector vaccines, mainly based on poxvirus vectors, have the advantages of actively infecting target tissues or cells, large vector capacity, intrinsic adjuvant effects of the vector, and the ability to induce long-term immune responses in most cases. These advantages align with the requirements for constructing an effective AIDS vaccine, making them a hot topic in vaccine research. Candidate AIDS vaccines using vaccinia virus as a vector have undergone several clinical trials, proving to have good safety, but their immunogenicity remains inconclusive[12-18]. (6) Boosting with live vector vaccines after DNA vaccine immunization has special advantages in inducing cellular immune responses and protective immune responses. It can significantly enhance the immune response induced by DNA vaccines and avoid the impact of immune responses against the vector on vaccine efficacy during multiple immunizations with live vector vaccines. Currently, using a DNA vaccine prime-live vector vaccine boost strategy to effectively induce HIV-specific CTL and neutralizing antibody responses has become a development strategy for the new generation of AIDS vaccines. Some candidate vaccines including the DNA prime-vaccinia virus boost strategy have entered clinical trials[19].

The world's first AIDS vaccine clinical trial was conducted in the United States in 1987. As of January 2006, 160 AIDS vaccine clinical trials have been conducted internationally, including 134 Phase I, 13 Phase I/II, 10 Phase II, and 3 Phase III trials, testing vaccine forms including: DNA vaccines, protein/peptide vaccines, recombinant viral vector vaccines, and

combinations of different vaccines. Phase II-III clinical trial results have shown that not only do protein subunit vaccines, which can only induce a certain humoral immune response, fail to protect humans from HIV infection, but even non-replicating viral vector vaccines such as ALVAC and MVA, which show good prospects in animal experiments, cannot induce sufficient humoral and cellular immune responses in humans, suggesting that viral vector vaccines lacking replication capacity in humans may not be sufficient to protect against HIV infection. In November 2004, the China Food and Drug Administration (SFDA) approved the "mixed AIDS vaccine" from Changchun BCHT Biotechnology Co., Ltd. to enter Phase I clinical trials in Guangxi. Currently, this clinical trial has ended, and the results are being analyzed.

2 Study Product

The vaccine applying for Phase I clinical trials this time is a DNA-Tiantan vaccinia complex AIDS vaccine, which includes a recombinant DNA vaccine and a recombinant vaccinia virus (rTV) vaccine.

2.1 DNA Vaccine

2.1.1 Current Status of AIDS DNA Vaccine Research

DNA vaccines can induce HIV- and simian immunodeficiency virus (SIV)-specific cytotoxic T lymphocytes (CTL), T-cell proliferative responses, and antibodies in mice and primates, and protect chimpanzees from HIV infection[20-21]. In a Phase I clinical trial of an HIV DNA vaccine conducted by the University of Oxford, 18 HIV-negative healthy volunteers were given two intramuscular injections of two DNA doses (100 or 500 micrograms) three weeks apart. The results showed that the human body tolerated both doses of DNA very well[22]. A preliminary trial of a DNA vaccine conducted by the National Institutes of Health (NIH) in the United States indicated that a single injection of a 3mg dose of DNA caused mild and rare adverse reactions[23]. In a Phase I clinical trial organized by the U.S. AIDS Vaccine Evaluation Group, multiple injections of a 3mg DNA dose at months 0, 1, 3, and 6 showed that all 52 volunteers tolerated the vaccine well and induced antigen-specific cell proliferation and chemokines[24]. In a recent human trial organized by Merck, the safety of 1mg and 5mg DNA vaccine doses was explored, with two different adjuvants used in the 5mg group, and immunizations given at weeks 0, 4, 8, and 26. The company reported its findings at the HIV Vaccine Trials Network meeting in May 2003: the human body tolerated the 5mg DNA dose very well; at week 12 post-immunization, the CTL response induced by the 5mg dose group

was significantly stronger than that of the 1mg dose group[25].

2.1.2 HIV-1 DNA Vaccine Vector

The HIV-1 DNA vaccine in this study utilizes the pDRVISV1.0 vector system, which carries a kanamycin resistance gene. DNA vectors containing this type of resistance gene have been approved by the U.S. FDA for use in humans.

2.1.3 HIV-1 DNA Vaccine

The DNA vaccine is composed of two plasmids, namely pGP140 and pGPNef. The HIV-1antigens in vaccines are from the major circulating CRF B'/C strain CN54 in China. The pGP140 plasmid expresses membrane protein Gp140TM, and pGPNef plasmid expresses Gag-pol-nef fusion protein GPNef. The gp140 and GPNef genes have been optimized for codon humanization, RNA stability and nuclear output signal, to improve the safety and expression efficiency. The information of DNA vaccine is shown in Table 1. Each of the two plasmids, pGP140 and pGPNef , have been produced 1,000 doses under Good Manufacturing Practice (GMP) conditions. All quality control tests have been passed, and the test results are detailed in Annex 4.

Table 1. Main information of HIV-1 DNA vaccine

Plasmid name	pGP140	pGPNef
Promoter	CMV	CMV
Full-length of plasmid (bp)	7118	9228
HIV-1 antigen genes	Env	gag, pol, nef
Solvent	20mM phosphate buffer, 0.9% NaCl, pH7.2	

2.2 Recombinant Vaccinia Virus Vector Vaccine

2.2.1 Research of Replicating Vaccinia Virus Vector Vaccine

Currently, Modified Vaccinia Virus Ankara (MVA), Avipoxvirus Canarypox (ALVAC) and Copenhagen Strain of Vaccinia Virus (NYVAC) are the most extensively studied and widely used poxvirus vectors. Clinical trials have shown that these non-replicating vaccinia virus vector vaccines are very safe in humans, but their immunogenicity is relatively weak, and the immune response induced in humans is significantly weaker than that in monkeys[12, 26].

Although the reasons for the large differences in immune effects between humans and animals are not yet clear, the weak immunogenicity of these vaccines may be related to the non-replicating nature of the vectors. After infecting host cells, these vector vaccines can only undergo one cycle of antigen expression, processing, and presentation, and cannot produce infectious viruses. Their stimulation of the immune system is limited and transient. In contrast, replicating vaccinia virus vectors can continuously and repeatedly stimulate the immune system over a longer period, thereby inducing a strong immune response in the body, and are expected to produce long-term immune protection and memory.

Replicating recombinant poxviruses can protect animals against many veterinary diseases. Currently, several recombinant poxvirus veterinary vaccines have been approved for use. Replicating recombinant poxviruses as vectors for hepatitis B virus, Epstein-Barr virus, and therapeutic cancer vaccines have entered the human immunization observation stage, and some have shown good immune effects[27-29]. In the SIV-rhesus monkey model, priming with a recombinant vaccinia virus expressing the SIV env gene and boosting with Env protein can protect rhesus monkeys from homologous virus challenge[30]. The Phase I clinical trial of a replicating recombinant vaccinia virus (HIVAC-1e) expressing HIV-1 env, developed by Cooney EL et al., showed that boosting once 8 weeks after priming with HIVAC-1e could induce HIV-1-specific T lymphocyte proliferative responses and Env-specific antibodies lasting more than a year in testers who had not been vaccinated against smallpox[31]. These results suggest that HIV vaccines using replicating poxviruses as vectors may have good application prospects.

Since its isolation in the 1920s, the vaccinia virus Tiantan strain has been used to prevent smallpox for decades, immunizing over a billion Chinese people. Vaccinia virus is generally harmless to humans, causing only local, short-term symptoms at the inoculation site, with a very low incidence of more serious adverse reactions. The incidence of post-vaccination encephalitis with the Tiantan strain is 0.5 per million, progressive vaccinia is 0 per million, and post-vaccination eczema and generalized vaccinia are 1 per million, making it safer than smallpox vaccine strains used in other countries. The high safety profile of the Tiantan strain ensures its suitability as a vector for other vaccines.

2.2.2 Recombinant Vaccinia Virus Vaccine

The vaccinia virus vector vaccine in this study is named the recombinant vaccinia virus (rTV) AIDS vaccine, and its vector is vaccinia virus Tiantan strain 752-1. This Tiantan strain

vaccinia virus was previously used in the smallpox vaccine administered to a large population in China.

The HIV-1 antigens also come from the major circulating CRF B'/C strain CN54. The optimized env (gp140TM), gag and pol genes were inserted into the TK region of the vector. To ensure the safety and immunogenicity of the vaccine, these HIV genes have been modified.

We have produced 50,000 doses of rTV vaccine under GMP conditions, and all quality control tests have been passed. The test results are detailed in Annex 5. The main information of rTV vaccine is presented in Table 2.

Table 2. Main information of rTV vaccine

**Vaccine**	**Recombinant vaccinia virus (rTV)**
**Vector**	Vaccinia virus Tiantan strain 752-1
**HIV-1 antigens**	gag, pol, env
**Titer**	1.6×10^{7} PFU/ml
**Solvent**	60% glycerol, 1 % to 2 % starch, 5.0 % albumose

2.3 Summary of Preclinical Trials

2.3.1 Safety Evaluation in Animal

The animal safety of HIV-1 DNA vaccine and rTV vaccine was entrusted to the National Center for Safety Evaluation of Drugs at the National Institutes for Food and Drug Control (NIFDC). The test results indicated that both the DNA vaccine and the rTV vaccine have good safety profiles in small animals.

2.3.2 Immunogenicity Study in Animal

Immunological experiments have shown that three doses of the DNA vaccine can induce strong antigen-specific humoral and cellular immune responses in mice; a fourth dose of the DNA vaccine did not generally enhance the immune response compared to three doses. Priming with three doses of the DNA vaccine followed by a booster with the recombinant vaccinia virus significantly enhanced the antigen-specific humoral immune response, although the cellular immune response varied with different antigens, showing both increases

and decreases. Overall, priming with the DNA vaccine followed by a booster with the recombinant vaccinia virus has advantages over DNA vaccine immunization alone.

Mouse immunization trials with rTV have shown that the intramuscular route tends to induce humoral immune responses, while the intradermal route may be a better pathway for inducing cellular immune responses, which aligns with the scarification route used for smallpox vaccination. The strength of the immune response induced by rTV is positively correlated with the immunization dose; as the dose increases, both cellular and humoral immune responses are enhanced.

Rhesus monkey immunization trials have demonstrated that the DNA vaccine induces strong antigen-specific humoral and cellular immune responses, which are significantly enhanced after boosting with rTV.

SHIV challenge trials in rhesus monkeys showed that after three immunizations with rTV, all monkeys (4/4) were protected against SHIV infection. After priming with DNA and boosting with rTV, 1/4 of the test monkeys were protected against SHIV infection, 2/4 showed viral loads 14 days post-challenge, but the peak viral loads were 2 logarithms lower than the average of the control group, and another monkey died of acute intestinal infection one week before the challenge, so no results were obtained. In contrast, 3 out of 4 control monkeys that received the empty vector had detectable virus replication by day 14 post-challenge. These results indicate that vaccination with the rTV AIDS vaccine can protect animals from SHIV infection.

Detailed information on the safety, immunogenicity, and monkey challenge trials of the vaccine can be found in the Investigator's Brochure.

2.4 Product Package, Doses and Method of Vaccination

The DNA vaccine is colorless and transparent liquid, packaged in a 2ml vial and each bottle contains 2.0mg/1ml. The label on the packaging indicates HIV-1 DNA vaccine (pGPNEF) or HIV-1 DNA vaccine (pGP140). It should be stored below -70℃. Each plasmid vaccine is extracted with 1ml, and injected using conventional intramuscular injection, with the injection site being the bilateral upper arm triceps.

The rTV vaccine is a light yellow or tan-colored turbid and viscous liquid. It is packaged in 0.4ml per vial. The label on the packaging indicates recombinant vaccinia virus (rTV) AIDS vaccine. It should be stored below -20℃. Vaccine administration is done using a bifurcated

needle skin prick method. The specific inoculation method is: insert the bifurcated needle into the vaccine bottle to coat it with enough vaccine liquid. Rest the wrist against the upper arm of the recipient, and vertically position the bifurcated needle over the deltoid muscle attachment site with a 5mm diameter range, prick 15 times quickly. The prick should be forceful, and there should be bleeding spots after 15 to 30 seconds. To inoculate the second pox on the same arm, change to another needle, coat it with vaccine, and use the same inoculation method to inoculate again outside the 2cm range of the first needle. Cover the inoculation site with gauze and breathable film after it dries up.

Notes: Skin scarification is a conventional method of smallpox vaccination in China, and its operation process is as follows: first, drop 10μl vaccine (estimated dose of 2×10^5 PFU) at the vaccination site and then scarifies to make the vaccine go through the damaged skin into body, but the actual amount of vaccine into body is much lower than 10μl. According to the international trend in this field, we use the immunization method with a bifurcated needle recommended by WHO in this clinical study. This method is simple in immunization procedure and easy to grasp as compared with the traditional ones. Using a bifurcated needle, dip for a volume of about 2.5μl, it has the same vaccination effect as a skin scarification vaccination using four times of dose (10μl). It ensures the effect of immunization while reducing the dosage of vaccine and improving the absorption rate of vaccine as compared to the traditional methods[32]. Each bifurcated needle dips this vaccine containing approximately 0.4×10^5 PFU of rTV, and its immunization effect is equivalent to that of an estimated dose of 1.6 × 105 PFU in a traditional skin scarification method.

The concentration of this rTV vaccine is 1.6×10^7 PFU/ml, lower than the concentration of 1.0 × 108 PFU/ml for smallpox vaccine recommended by WHO. In a recent study, however, the smallpox vaccine was diluted by 5 folds and 10 folds to the concentrations of approximately 0.5×10^8PFU/ml and 1.0×10^7PFU/ml, and was vaccinated using a bifurcated needle in volunteers. The results showed that the success rate was 98% to 100% for three concentrations of vaccines, and that there were no significant differences in the size of timing of skin lesions after vaccination of the three vaccines[33,34]. These results suggest that the concentration of this vaccine may not affect its vaccination effect.

3 Objectives and Rationale for the Study

The objective of this clinical trial is to test the safety and immunogenicity of the HIV-1 DNA vaccine and the recombinant vaccinia virus vector vaccine in healthy volunteers. This study has chosen the prevalent HIV strain in China—the B'/C recombinant strain CN54 (CRF07)—as the pathogen model for designing the AIDS vaccine, providing a solid foundation for future vaccine evaluation and application. Existing research data indicate that DNA vaccines can induce HIV- and SIV-specific protective immune responses in mice and primates, and there have been no reports of severe adverse reactions in humans following vaccination with such vaccines. Candidate AIDS vaccines using vaccinia virus as a vector have undergone several clinical trials, demonstrating good safety and the ability to induce certain HIV-specific immune responses. The safety of the replicating vaccinia virus Tiantan strain vector we have adopted has been tested in large-scale population applications.

Preclinical trial results have shown that the DNA vaccine and recombinant vaccinia virus vaccine we developed are well-tolerated in animals and can induce HIV-1 antigen-specific cellular and humoral immune responses. Therefore, we anticipate that these two vaccines will have good safety profiles in humans and are expected to induce favorable immune responses.

4 Research Institutions and their Responsibilities

This trial is funded by the National High-Tech Research and Development Program of China (863 Program) and is supervised by the Clinical Pharmacology Trial Ethics Review Committee of Peking Union Medical College Hospital (PUMCH) and the Ethics Review Committee of NCAIDS. Major research institutions to participate in this clinical trial and their responsibilities are shown in Table 3.

Table 3. The major research institutions of clinical trial and their responsibilities

Research institution	Team leader	Responsibilities
NIFDC	Sang, Guowei<br>Wang, Youchun	Clinical trial guidance
PUMCH	Li, Taisheng	Conduct the Phase Ia clinical trial
NCAIDS	Shao, Yiming	Provide AIDS vaccine (DNA), and organize the clinical trial
Beijing Institute of Biological Products	Xu, Jing	Provide AIDS vaccine (rTV)
Health Statistics Office, China CDC	Jin, Shuigao	Clinical trial data management and statistical analysis

4.1 Responsibilities of Sponsor(s)

NCAIDS and the Beijing Institute of Biological Products are the sponsors of the clinical trial, with responsibilities as follows:

(1) Confirm the qualifications and conditions of the investigators at the clinical trial sites to ensure the completion of the trial;

(2) Provide the investigators with the Investigator's Brochure, which includes

pharmaceutical, toxicological, and preclinical data and information about the test vaccine;

(3) Begin organizing the clinical trial according to this protocol after obtaining approval from SFDA and consent from the ethics committee;

(4) Collaborate with the investigators to design the clinical trial protocol, specifying responsibilities in data processing, statistical analysis, result reporting, and publication methods, as well as the division of tasks agreed upon with the investigators. Sign the mutually agreed trial protocol and contract;

(5) Assist investigators in recruiting subjects;

(6) Provide investigators with the test vaccine and instructions for use, ensuring the vaccine's quality is qualified. Establish a management system and record-keeping system for the registration, storage, and distribution of the test vaccine;

(7) Appoint monitors acceptable to the investigators to oversee the progress of the clinical trial;

(8) Be responsible for establishing a quality control and quality assurance system for the clinical trial. If necessary, organize audits of the clinical trial to ensure quality;

(9) Collaborate with investigators to promptly study any serious adverse events that occur, take necessary measures to ensure the safety of subjects, and report to the drug regulatory authorities in a timely manner. Also, inform other investigators involved in clinical trials of similar vaccines about the adverse events;

(10) Notify investigators, the ethics committee, and the State Food and Drug Administration promptly if the clinical trial is terminated or suspended early, stating the reasons;

(11) Submit the trial's summary report to the State Food and Drug Administration, or provide a report on the termination of the trial and the reasons for it;

(12) Be responsible for covering the treatment or handling costs for subjects who are injured or die due to vaccination during the clinical trial, and provide appropriate financial compensation to those who experience serious adverse reactions. Provide subjects with accidental injury insurance during the trial period;

(13) If investigators do not follow the approved protocol, Good Clinical Practice (GCP), or relevant regulations in conducting the clinical trial, the sponsor should point this out to

seek correction. If the situation is serious or persists, the sponsor should terminate the investigator's participation in the clinical trial and report to the State Food and Drug Administration.

4.2 Responsibilities of Investigator

PUMCH is the investigator for this clinical trial, with responsibilities as follows:

(1) Investigators participating in the clinical trial should be familiar with Good Clinical Practice (GCP), comply with relevant national laws, regulations, and ethical standards, have extensive experience in clinical trial research methods or receive academic guidance from experienced researchers within their institution, and possess the professional knowledge and experience required by the trial protocol;

(2) Be familiar with the materials and literature provided by the sponsor related to the clinical trial; understand and be familiar with the nature, effects, and safety of the investigational vaccine (including relevant data from preclinical studies), and also be aware of all new information related to the vaccine discovered during the course of the clinical trial;

(3) Have and be able to allocate the necessary personnel and equipment for the trial; possess all facilities to handle emergencies to ensure the safety of subjects;

(4) Laboratory testing methods must be nationally certified, and laboratory results must be accurate and reliable;

(5) Must thoroughly read and understand the content of the trial protocol, sign the clinical trial protocol jointly with the sponsor, and strictly adhere to the protocol's stipulations; should promptly submit the clinical trial protocol and informed consent forms to the ethics committee for approval;

(6) Must obtain the consent of their hospital, ensuring sufficient time to be responsible for and complete the clinical trial within the period specified by the protocol; must explain the trial's information, regulations, and responsibilities to all staff involved in the clinical trial, ensuring an adequate number of subjects who have given informed consent and meet the inclusion criteria enter the clinical trial;

(7) Take necessary measures to protect the safety of subjects and document these measures. Be responsible for making medical decisions related to the clinical trial, ensuring that subjects receive appropriate treatment for any adverse events that occur during the trial;

(8) In the event of serious adverse events during the clinical trial, immediately provide appropriate treatment to the subjects, report to the drug regulatory authorities, the sponsor, and the ethics committee, and sign and date the report;

(9) Ensure that subject data is accurately, completely, timely, and legally recorded in the case report forms;

(10) Accept monitoring and auditing by the sponsor's appointed monitors or auditors, as well as inspections by the drug regulatory authorities, to ensure the quality of the clinical trial;

(11) Upon completion of the clinical trial, the investigator must write a summary report, sign and date it, and then send it to the sponsor;

(12) Must notify subjects, the sponsor, the ethics committee, and the State Food and Drug Administration if the clinical trial is terminated or suspended early, stating the reasons.

4.3 Responsibilities of the Data Management Center

The Health Statistics Office of the Chinese Center for Disease Control and Prevention (CDC) will serve as the data management center for this clinical trial, with responsibilities as follows:

(1) To be responsible for the statistical design of the clinical trial;

(2) To provide statistical requirements for the design of the Case Report Forms (CRFs) and participate in the revision of the CRFs;

(3) To complete the randomization process and blinding design according to the research requirements, and assist researchers in implementing the blinding;

(4) To complete the randomization process according to the research requirements;

(5) To be responsible for developing the Data Management Plan (DMP), organizing data entry, data checking, and management;

(6) To be responsible for developing the Statistical Analysis Plan (SAP), completing statistical analysis and reporting of results;

(7) To be responsible for developing the Study Monitoring Plan (SMP), completing the monitoring of the entire study;

(8) To be responsible for regularly submitting data and safety reports to the Data Safety Monitoring Board (DSMB).

5 Phase Ia protocol

5.1 Objectives

Primary Objective:

To observe the safety and tolerability of a single inoculation of the rTV vaccine by bifurcated needle with either one or two doses in healthy volunteers who have or have not previously been vaccinated against smallpox;

Secondary Objective:

To preliminarily test the immunogenicity of a single inoculation of the rTV vaccine by bifurcated needle with either one or two doses in healthy volunteers who have or have not previously been vaccinated against smallpox.

5.2 Endpoint Events

The endpoint events of the study refer to the endpoint indicators that are expected to be observed in the clinical trial. The results of the clinical trial will be analyzed based on the frequency of occurrence of these endpoint events.

5.2.1 Primary Endpoint Events

5.2.1.1 Primary Safety Endpoint Events

During the clinical trial, safety indicators will be graded according to Annex 2, and the following will be considered as primary safety endpoint events:

(1) Grade 3 or higher local adverse events;

(2) Grade 3 or higher systemic adverse events;

(3) Grade 3 or higher laboratory test adverse events;

(4) Any event leading to the termination of the immunization regimen due to vaccine-related issues.

5.2.1.2 Primary Immunogenicity Endpoint Events

HIV-specific T-cell responses will be measured by the ELISPOT method at 2, 4, 8, and 24 weeks after the last vaccination in each group.

5.2.2 Secondary Endpoint Events

Secondary safety and immunogenicity endpoint event will be collected according to the following criteria:

(1) All Grade 1 and 2 adverse events occurring within 4 weeks after each vaccination;

(2) The following responses will be tested at 2, 4, 8, and 24 weeks after the last vaccination in each group:

- Total HIV antibodies, Env, Gag binding antibody titers, and HIV neutralizing antibody titers;
- The proportion of HIV-specific IFN-γ, IL-2, and other cytokines;
- The binding antibody titer of the vaccinia virus after rTV immunization.

5.3 Recruitment of Study Subjects

Healthy volunteers will be recruited by NCAIDS through promotions in hospitals, universities, and social organizations. Detailed information about the clinical trial will be provided to those interested in participating, and all questions regarding the trial details will be answered. For those still willing to participate, informed consent for subject screening will be obtained (see Annex 1-II, Part 1), and they will be referred to PUMCH for screening, along with notification of their screening registration number. PUMCH will organize the collection of personal information, physical examinations, specimen collection, and laboratory tests for the volunteers. For volunteers who meet the inclusion criteria, informed consent for participation in the clinical trial will be obtained (see Annex 1-II, Part 2), and they will be assigned a group number in preparation for entering the clinical trial. The flowchart for subject recruitment and screening is as follows:

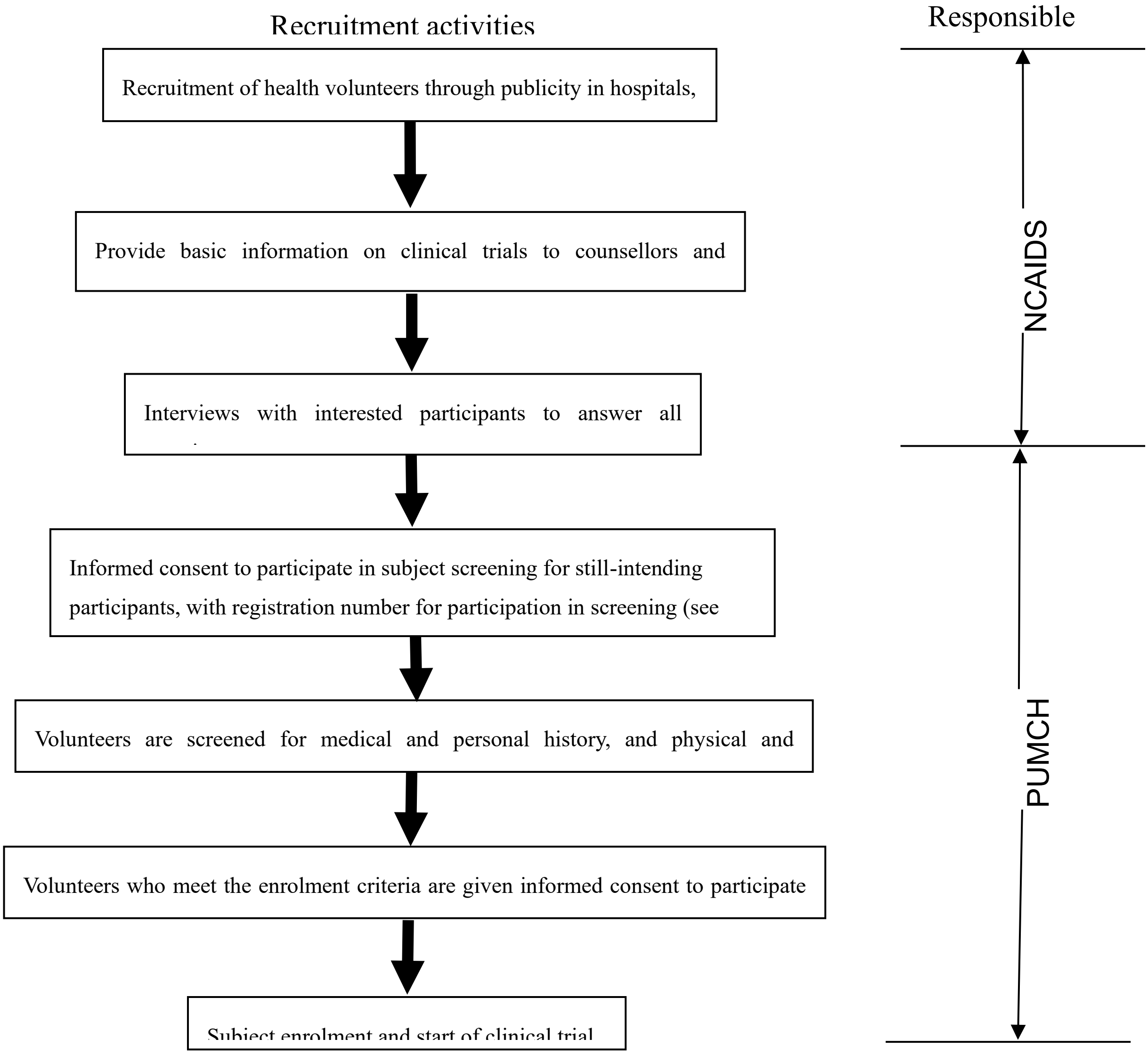

Figure 1. Flowchart for Subject Recruitment and Screening

5.4 Protocol

This Phase I clinical trial is conducted in two stages, Ia and Ib, requiring 48 subjects, with a total study duration of approximately 58 weeks. The Phase Ia requires 12 subjects and lasts for 26 weeks, primarily observing the safety and tolerability of a single inoculation of the rTV vaccine by bifurcated needle with either one or two doses in healthy volunteers who have or have not previously been vaccinated against smallpox, and preliminarily observing its immunogenicity.

The criteria for determining individuals who have been vaccinated against smallpox must meet at least two of the following conditions: (1) age ≥ 27 years; (2) presence of a typical smallpox vaccination scar on the upper arm skin; (3) positive vaccinia antibody test. The

criteria for determining individuals who have not been vaccinated against smallpox must meet all three of the following conditions: (1) age < 27 years; (2) absence of a typical smallpox vaccination scar on the upper arm skin; (3) negative vaccinia antibody test.

5.4.1 Inclusion and Exclusion Criteria

Inclusion criteria

(1) Gender is not restricted;

(2) Age between 18 and 55 years;

(3) Able to undergo follow-up within 6 months after vaccination and agree to venous blood sampling and specimen storage;

(4) Capable of understanding and agreeing to the content of the informed consent form;

(5) At low risk for HIV infection:

- No history of intravenous drug use
- No history of gonorrhea or syphilis in the past year
- No current or past high-risk sexual partners (e.g., intravenous drug users, HIV-positive partners)
- No unprotected sexual intercourse with non-regular partners in the past year

(6) Willing to undergo HIV and syphilis blood tests;

(7) Willing to use effective contraception with their sexual partners from 2 weeks before immunization until 6 months after immunization, and female volunteers are willing to undergo urine pregnancy tests before immunization and during follow-up.

Exclusion criteria

(1) Pregnant or breastfeeding, or planning to become pregnant during the trial, or having close contacts who are pregnant or breastfeeding within 1 month after rTV vaccination;

(2) Having the following diseases or medical history:

- Congenital or acquired immunodeficiency diseases such as AIDS, or having close contacts with such patients within 1 month after rTV vaccination;
- Requiring treatment that affects immune responses, such as: use of corticosteroids for

more than two weeks, or use of immunosuppressants such as alkylating agents, antimetabolites, or receiving radiation therapy, etc.; or having close contacts who are receiving such treatments within 1 month after rTV vaccination;

- Having immunosuppressive diseases such as malignant tumors, organ or stem cell transplantation, agammaglobulinemia, etc.; or having close contacts with such patients within 1 month after rTV vaccination;
- Previous or current history of eczema or atopic dermatitis; currently suffering from diseases that cause skin damage such as: burns, scalds, chickenpox, impetigo, shingles, psoriasis, etc.; or having close contacts with such patients within 1 month after rTV vaccination;
- Previous or current history of hypertension, heart disease, diabetes, thyroid disease, asthma, angioneurotic edema, asplenia, mental illness, psychological illness, epilepsy, etc.;
- Having diseases that require repeated injections or blood sampling;
- History of fainting or allergies after vaccination;
- Currently suffering from acute infectious diseases and febrile illnesses;

(3) Having the following situations:

- Received live attenuated vaccines within the last 2 months or other vaccines within the last 2 weeks;
- Received immunoglobulin or other blood products within the last 4 months;
- Participated in other medical product trials within the last 1 month;
- Drug abuse, drug dependence, alcoholism, heavy smoking;

(4) Abnormal laboratory test results: The following laboratory test results are excluded if they exceed the normal reference range and are judged by the investigator to have no clinical significance.

- HIV antibody positive or suspicious, HIV nucleic acid test positive;
- Hepatitis B surface antigen or hepatitis C antibody positive, syphilis rapid plasma reagin test (RPR) positive;
- Immunoglobulin G, A, M values are not within the normal range;

- Lymphocyte phenotype analysis shows CD3+, CD4+, CD8+, CD4+CD28+, CD8+CD28+, CD8+DR+, CD8+CD38+ cell values are not within the normal range;
- Hemoglobin ≤110g/L for females, ≤120g/L for males;
- White blood cell count ≤3.3×10^9/L or ≥12.0×10^9/L;
- Total lymphocyte count ≤0.8×10^9/L;
- Platelet count ≤100×10^9/L or ≥300×10^9/L;
- Biochemical indicators: alanine aminotransferase (ALT), aspartate aminotransferase (AST), myocardial enzymes (CK), fasting blood glucose, total bilirubin (TBIL), blood urea nitrogen (BUN), creatinine (Cr), etc. exceed the normal reference range (excluded if judged by the investigator to have no clinical significance);
- Urine glucose ≥15mmol/L, urine protein ≥0.3g/L;
- Unable to comply with the study protocol or obtain informed consent due to medical, psychological, social, occupational, or other reasons.

5.4.2 Immunization and Follow-up Plan

The Phase Ia clinical trial includes 12 subjects, consisting of individuals who have been vaccinated against smallpox and those who have not. The subjects are divided into two groups: a low-dose group and a high-dose group, each with 6 participants. The low-dose group receives one dose of the rTV vaccine via a bifurcated needle, while the high-dose group receives two doses of the rTV vaccine two weeks later. Safety assessments of the rTV vaccine are conducted immediately after the low-dose administration. If the number of grade 3 or higher adverse events related to vaccination in the low-dose group is ≤2, the high-dose group trial will proceed. If three or more grade 3 or higher adverse events related to vaccination occur in the low-dose group (excluding fever above 39℃ for ≤3 days), the subsequent clinical trial will be terminated.

The maximum safe dose of the rTV vaccine for use in the Phase Ib study will be determined based on the results of the Phase Ia trial. The criteria for determining the severity of adverse events and their relationship to vaccination are detailed in Annex 2 and Table 8.

After immunization, subjects will be observed for 24 hours, during which researchers will closely monitor local and systemic adverse reactions and complete the CRF forms. Subjects will then be provided with daily diary cards and instructed on how to fill them out. Subjects

are required to complete the diary cards daily for 4 weeks post-vaccination and submit them to researchers during each follow-up visit. If subjects agree, researchers may arrange to meet with them every other day post-vaccination to observe post-vaccination reactions until the scab at the vaccination site falls off (approximately 2-3 weeks). Follow-up visits at the hospital will occur on the 3rd day, 1st week, 2nd week, 4th week, 8th week, 16th week, and 24th week post-vaccination. During each follow-up, researchers will record all subject-related information, including physical examinations and laboratory tests, in medical records and transcribe them onto CRF forms. During the trial, Phase Ia subjects will need to visit PUMCH at least 9 times for follow-ups and venous blood sampling.

Based on the occurrence of smallpox vaccination reactions, after vaccinia virus vaccination, the local site will undergo a typical process of papule, vesicle, pustule, scab formation, and scab detachment, which takes about 3 weeks. General systemic reactions and rare abnormal adverse reactions to smallpox vaccination also commonly occur within 3 weeks post-vaccination. Accordingly, this Phase Ia trial will be conducted in two stages: the acute reaction observation phase post-vaccination and the convalescent reaction observation phase.

Table 4. Acute Reaction Observation Phase Trial Plan

**Visit week**		**-4～-2**	**-1**	**0**	**0.5**	**1**	**2**	**4**	**8**
**Pre-screening**									
**Screening**	**Informed consent for screening**								
	**Personal information collection**								
	**Physical examination**								
	**Laboratory tests**								
**Enrollment**	**Informed**								

**and Vaccination**	**consent for trial participation**								
	**Vaccination**								
**Acute Reaction Observation**	**AEs collection**								
	**Immune response testing**								
	**Vaccinia virus culture**								

The convalescent reaction observation phase spans weeks 9-26 of the trial, during which local and systemic adverse reactions occurring within 24 weeks post-rTV vaccination will continue to be observed. Additionally, during both the acute and convalescent reaction observation phases, the immune responses of subjects to HIV antigen-specific stimuli will be tested.

The immunization schedule, follow-up schedule, and venous blood sampling schedule for Phase Ia are detailed in Tables 5, 6, and 7, respectively.

Table 5. Phase Ia Immunization Schedule

**Group**	**Number of subjects**	**Week**		
		**0**	**2**	**3～26**
Low-dose	6	One pox		Follow-up
High-dose	6		Two poxes	Follow-up

Table 6. Follow-up schedule

Visit Week	-2	0	0.5	1	2	4	8	16	24
**Personal Information Collection/HIV Testing Consultation**	x				x	x	x	x	x
**Physical Examination**	x	x	x	x	x	x	x	x	x
**Chest X-ray (PA view)**	x								x
**Electrocardiogram (ECG)**	x			x	x	x	x	–	x
**Complete Blood Count (CBC)**	x			x	x	x	x	x	x
**Urinalysis**	x			x	x	x	x	x	x
**Liver and Kidney Function Tests and Myocardial Enzymes**	x			x	x	x	x	–	x
**HIV Antibody Test**	x				x	x	x	x	x
**HIV Nucleic Acid Test a**	x								x
**Hepatitis B Surface Antigen and Hepatitis C Antibody Tests**	x								x
**Syphilis Test**	x								x

**Urine Pregnancy Test**	x	x					x	x	x
**Immune Function Tests**	x								x
**HLA Typing**		x							
**Immune Response Testing**									
**Humoral Immune Response (ELISA) b**		x			x	x	x	x	x
**Cellular Immune Response (ELISPOT)**		x			x	x	x	x	x
**Intracellular Staining (ICS)**		x			x	x	x	x	x
**HIV Neutralizing Antibody c**		x				x	x	x	x
**Vaccinia Virus Antibody Test**	x				x	x	x	x	x
**Vaccinia Viremia Detection**			x	x	x	x			

Note: "-" indicates that if there were abnormal changes in the previous test, continued testing is required. "a" After vaccination, if the HIV antibody test is positive, an HIV nucleic acid test is also required. "b, c" Specimen collection at the corresponding time points; when the total HIV antibody test is positive, Env and Gag binding antibody (b) and neutralizing antibody (c) tests are conducted.

Table 7. Venous Blood Collection Sheet (Vaccination Week is Week 0) Unit: ml

Visit Week	-2	0	0.5	1	2	4	8	16	24
Complete Blood Count	2E			2E	2E	2E	2E	2E	2E
Liver and Kidney Function Tests	2			2	2	2	2	—	2
Myocardial Enzymes								—	
Syphilis RPR	2								2
Immunoglobulins G, A, M	2								2
Hepatitis B Surface Antigen and Hepatitis C Antibody Tests	2								2
HIV Antibody Test					2	2	2	2	
T Cell Phenotype Analysis	4E								16E
HIV Nucleic Acid Test*					*	*	*	*	
Humoral Immune Response (ELISA)		14E			14E	14E	14E	14E	
Cellular Immune Response									

**(ELISPOT)**									
**Vaccinia Virus Antibody Test**	**2E**				**16E**	**16E**	**16E**	**16E**	**16E**
**Intracellular Staining (ICS)**		**16E**							
**Cell Cryopreservation**									
**HIV Neutralizing Antibody Test**									
**HLA Typing**									
**Vaccinia Virus Culture**			**7E**	**7E**	**7E**	**7E**			
**Total Blood Collection Volume**	**16**	**30**	**7**	**11**	**43**	**43**	**36**	**34**	**42**

Note:

1. Samples marked with "E" indicate EDTA anticoagulated blood; all others are non-anticoagulated blood.

2. "－" If there were abnormal changes in the previous test, continued testing is required.

3. "*" If the HIV antibody test is positive after vaccination, HIV nucleic acid testing must be performed simultaneously.

4. Tests conducted at the NCAIDS laboratory require the samples to be blinded by the Infectious Diseases Laboratory of Union Hospital. After testing is completed, the original results must be submitted to the Infectious Diseases Laboratory, where the blinding will be removed by laboratory personnel.

5.4.3 Determination of Success of rTV Vaccination and Re-vaccination

As this trial is the first clinical study of the rTV vaccine, the vaccination reactions induced by this vaccine can only be determined after the completion of the clinical trial. However, the reactions observed after vaccination with the TianTan strain smallpox vaccine can serve as a reference for the expected reactions following rTV vaccination.

Based on experience with smallpox vaccination, first-time vaccine recipients typically exhibit a series of skin reactions at the vaccination site, including papules, vesicles, pustules, scabbing, and scab shedding. This process generally takes about 3 weeks (see Annex 6 for details). For those revaccinated with the vaccinia virus, the same process occurs after successful vaccination, but the reactions are milder and of shorter duration. In the case of the rTV vaccine, the insertion of the HIV gene has significantly reduced the virulence of the Tian Tan strain vaccinia virus. Animal studies have also shown that the toxicity of the rTV vaccine is 100 to 1000 times lower than that of the Tian Tan strain vaccinia virus. Therefore, the skin reactions induced by the rTV vaccine in humans may be milder than those caused by the Tian Tan strain smallpox vaccine. Additionally, due to technical issues during administration, the vaccination site may only show mild needle trauma reactions without the typical pox formation, which indicates vaccination failure.

For the reasons mentioned above, rTV vaccination failure can be determined if both of the following conditions are met: (1) The vaccination site does not exhibit the aforementioned pox formation; (2) Serum vaccinia antibodies do not seroconvert (for those initially negative for vaccinia antibodies) or do not show an increase in antibody titer compared to pre-vaccination levels (for those initially positive for vaccinia antibodies) two weeks after vaccination. For individuals who fail the rTV vaccination, a re-vaccination will be administered at the same dose on the opposite upper arm four weeks after the first vaccination. Follow-up procedures for re-vaccinated participants will be conducted in the same manner as after the initial vaccination.

5.5 Vaccine Safety Evaluation

On the day of vaccination or follow-up, the research staff should record the subjects' symptoms, physical signs, and laboratory test results in the Case Report Form (CRF). Any discomfort experienced by the subjects outside the hospital should be recorded by the subjects themselves on the Daily Diary Card (Annex 3). During the next follow-up visit,

adverse events occurring between the two follow-ups should be documented in the medical records, transcribed into the CRF, and entered into the database. In case of discrepancies between the CRF and the Daily Diary Card, the records in the CRF shall prevail.

5.5.1 Evaluation of Adverse Events and Their Relationship to the Investigational Vaccine

An adverse event refers to any unfavorable experience that occurs to a subject during the clinical trial. A serious adverse event is an adverse experience that results in any of the following outcomes: (1) death; (2) life-threatening conditions; (3) requiring hospitalization or prolongation of existing hospitalization (excluding hospitalization due to pre-existing conditions); (4) persistent or significant disability or incapacity; (5) congenital anomalies or birth defects (referring to consequences resulting from the subject's pregnancy); (6) other significant medical events (e.g., allergic reactions requiring emergency treatment for bronchospasm, seizures not requiring hospitalization or medication, etc.). Systemic and local adverse events, as well as laboratory test-related adverse events, will be graded according to the criteria in Annex 2. Social harm should also be classified as an adverse event.

Based on their relationship to the investigational product, adverse events can be categorized into five types: definitely unrelated, possibly unrelated, possibly related, definitely related, or undetermined. The latter three categories are defined as adverse reactions caused by vaccination and are considered adverse events related to vaccination. Specific criteria for determining the relationship between adverse events and the investigational vaccine are outlined in Table 8.

Table 8. Relationship Between Adverse Events and the Investigational Vaccine

**Definitely Unrelated**	The symptoms differ from the expected post-vaccination reactions or are not temporally related to the vaccination. The symptoms may be caused by other diseases or treatments, and they resolve or disappear after the disease improves or the treatment is stopped.
**Possibly Unrelated**	The symptoms differ from the expected post-vaccination reactions or are not temporally related to the vaccination. The symptoms may be caused by other diseases or treatments.
**Possibly Related**	The symptoms are similar to the expected post-vaccination reactions and/or align with the expected timing of post-vaccination reactions. The symptoms resolve or improve after vaccination is stopped and cannot be explained by other causes.

**Definitely Related**	The symptoms and their timing are consistent with the expected post-vaccination reactions. The symptoms resolve or improve after vaccination is stopped and reappear upon re-vaccination.
**Unable to Determine**	The symptoms may be caused by factors outside the trial but are similar to post-vaccination reactions and/or their timing may be related to vaccination. It cannot be ruled out that the symptoms are adverse events caused by the vaccine.

The grading of adverse events is determined after review by the head of the clinical trial unit. The relationship between adverse events and vaccination is initially assessed by the researchers of the clinical trial unit but must be finalized in writing after discussion between the sponsor and the clinical trial unit.Evaluation of Local Adverse Reactions

5.5.1.1 Evaluation of Local Adverse Reactions

Local adverse reactions are evaluated according to the criteria outlined in Annex 2, with the following explanations:

(1) Pain at the vaccination site: Recorded by the subject themselves.

(2) Skin erythema, redness, swelling, papules, or induration at the injection site: Graded based on the maximum diameter of the reaction, duration, and other factors.

(3) Blisters or ulcers: Graded according to their size, depth, healing time, nature of the blister (e.g., whether it is bloody), duration, and the presence of necrosis.

(4) Pustules: Graded based on their size, duration, and the presence of necrosis.

(5) Generalized rash: Graded according to the number of papules, presence of itching, exudation, and other factors.

5.5.1.2 Evaluation of Systemic Adverse Reactions

(1) Before vaccination and within 2 hours after vaccination, researchers will measure the subject's body temperature and grade it according to Annex 2. Each subject will be provided with a thermometer. Subjects should measure and record their body temperature daily from the day of vaccination until 28 days post-vaccination on the daily report form. If the body temperature remains above 37°C at 28 days post-vaccination, the subject should continue to measure and record daily until the temperature returns to normal. The body temperature measured by researchers during follow-up visits should be recorded in the medical records and transcribed into the CRF.

(2) From the day of vaccination until 28 days post-vaccination, subjects should record symptoms such as chills, headache, nausea, vomiting, lethargy, and generalized myalgia on the daily diary card. If these symptoms persist beyond 28 days post-vaccination, the subject should continue recording until the symptoms resolve. During follow-up visits, researchers should document the collected information in the medical records and transcribe it into the CRF. In case of discrepancies between the CRF and the daily diary card, the records in the CRF shall prevail for data analysis.

5.5.1.3 Evaluation of Ocular Adverse Reactions

During the period of skin reactions following rTV vaccination, if a subject touches the vaccination site or its covering and then touches their own or another person's eyes without disinfection, there is a possibility of developing vaccinia-related eye diseases (e.g., blepharitis, conjunctivitis, keratitis). If the subject or their close contacts experience ocular symptoms, they should immediately seek medical attention at the clinical research unit. The diagnosis should be recorded in the medical records and transcribed into the CRF.

5.5.1.4 Evaluation of Other Clinical Abnormalities

For other adverse reactions, subjects should promptly record the start and end dates as well as any treatment measures taken on the daily diary card. These reactions should be graded according to the criteria in Annex 2. For clinical abnormalities not covered in Annex 2, the severity should be assessed based on the criteria outlined in Table 9.

Table 9. Criteria on intensity grading of adverse effects

**Grade 1**	Mild	A discomfort shorter than 48 hours, not requiring medical treatment
**Grade 2**	Moderate	Mild to moderate limitation of daily activities, not requiring or requiring only a little medical intervention
**Grade 3**	Severe	Significantly restrict daily activities, requiring the care of daily living, requiring medical treatment, and may need to be hospitalized
**Grade 4**	Life-threatening	Extremely restrict daily activities, significantly requiring the care of daily living, requiring medical treatment and hospitalization

5.5.1.5 Evaluation of Laboratory Test Results

The following tests will be conducted at the certified laboratory of PUMCH in accordance with standard operating procedures. Abnormal test results will be graded according to the criteria in Annex 2.

(1) Hematology tests: Hemoglobin, white blood cell count, platelet count.

(2) Blood biochemistry: ALT, AST, total bilirubin, blood urea nitrogen, creatinine, cardiac enzymes, etc.

(3) Urinalysis: Urine protein, urine glucose, blood cells.

5.5.2 Reporting of Adverse Events

All adverse events should be documented in the CRF and entered into the database. The documentation should include a general description of symptoms and signs, dates of onset and resolution, severity, relationship to the investigational vaccine or other medications, and measures taken to alleviate the symptoms.

If a subject experiences a serious adverse event during the clinical trial, the investigator must immediately provide appropriate treatment to the subject. Simultaneously, a Serious Adverse Event Report Form (see Annex 8) should be completed and submitted within 24 hours to the Safety Supervision Department of the State Food and Drug Administration, with copies sent to the Registration Department, the sponsor, the ethics committee, and the higher-level health administration authorities. The report should include the rationale for diagnosing the event as a serious adverse event, and be signed and dated by the investigator. Grade 3 adverse events should be reported to the clinical trial sponsor within 2 working days after the decision is made.

5.5.3 Management of Adverse Events

When a subject experiences an adverse event during the clinical trial, the investigator at the clinical trial site should closely monitor the subject and provide appropriate treatment. The following principles should be followed:

(1) Immediate treatment for serious adverse events.

(2) Discontinuation of vaccination for the subject if any of the following adverse events

occur:

- Symptoms appearing within 2 hours after vaccination, including:Respiratory symptoms: chest tightness, wheezing, palpitations, difficulty breathing, etc.; Circulatory failure symptoms: pallor or cyanosis, bradycardia, weak or absent pulse, hypotension, hypothermia, cold extremities, etc.; Central nervous system symptoms: confusion, convulsions, coma.
- Grade 3 or higher systemic or local adverse events, or laboratory adverse events related to vaccination.

(3) Postponement of the next vaccination for subjects experiencing other adverse events until the abnormal changes resolve, while continuing clinical observation and providing appropriate treatment.

(4) Evaluation and management of adverse events following rTV vaccination should be conducted in accordance with the principles for adverse reactions and management after smallpox vaccination (see Annex 6).

In the event of a serious adverse event requiring emergency unblinding during the clinical trial, the principal investigator and the sponsor should jointly perform the unblinding.

5.5.4 Immunogenicity Evaluation

5.5.4.1 Antibody Response

The following experiments will be conducted according to standard operating procedures:

- Detection of total HIV antibodies and Env, Gag antibodies using ELISA. Confirmation of experimental results will be performed by analyzing the following plate readings: (1) samples from all subjects at the same time point; (2) randomly selected samples from the same subject at different time points.
- Detection of HIV neutralizing antibodies using the TZM-bl method.
- Detection of vaccinia virus binding antibodies using ELISA.

5.5.4.2 Cellular Immune Response

- ELISPOT assays will be performed using freshly isolated PBMCs stimulated with Env, Gag, and Pol peptide pools. Results will be measured using an automated plate reader.
- Intracellular staining will be performed using freshly isolated PBMCs stimulated with

Env, Gag, and Pol peptide pools. Results will be measured using flow cytometry.

5.5.4.3 Storage of HLA Specimens

To assist in further evaluation of cellular immune responses, peripheral blood samples from subjects will be stored at enrollment for HLA typing.

5.5.4.4 Detection of Vaccinia Viremia

Vaccinia viremia will be detected using viral culture methods.

5.5.5 Early Termination of the Trial

5.5.5.1 Early Withdrawal of Subjects or Termination of Vaccination

Subjects may withdraw from the trial at any time for any reason. The following situations require early termination of a subject's participation. Researchers must record the time and reason for withdrawal in the medical records and transcribe them into the CRF.

(1) HIV antibody positivity not related to vaccination (subsequent vaccinations will be stopped, but follow-up will continue).

(2) Pregnancy (for pregnancies occurring after completion of all vaccinations, follow-up should continue with the subject's consent to ensure safety).

(3) Adverse events following vaccination:

- Symptoms appearing within 2 hours after vaccination, including: Respiratory symptoms: chest tightness, palpitations, difficulty breathing, etc.; Circulatory failure symptoms: pallor or cyanosis, bradycardia, weak or absent pulse, hypotension, cold extremities, etc.; Central nervous system symptoms: confusion, convulsions, coma.
- Grade 3 or higher systemic or local adverse events, or laboratory adverse events related to vaccination.

For subjects terminating the trial due to adverse events, the reason and date of termination should be recorded on the adverse event report form and submitted within 24 hours to the Safety Supervision Department of the State Food and Drug Administration, the sponsor, and the IRB. Follow-up should continue as much as possible until the expected end of the trial, or at least until the adverse event stabilizes or resolves.

(4) Failure to attend follow-up visits or provide samples multiple times, significantly affecting data collection. The sponsor and clinical trial unit may decide to terminate the

subject's participation early.

5.5.5.2 Early Termination of the Clinical Trial

If three or more vaccine-related adverse events of grade ≥3 (excluding fever above 39°C lasting ≤3 days) occur in both the previously vaccinated and unvaccinated groups after low-dose (1 pock) rTV vaccination in Phase Ia, this Phase I clinical trial will be terminated.

5.6 HIV-Positive Related Issues and Management During the Trial

5.6.1 HIV Antibody Positivity Induced by Vaccination

Subjects may test positive for HIV antibodies after vaccination. Upon the subject's request, NCAIDS will conduct additional nucleic acid testing to differentiate between HIV infection and HIV antibody positivity caused by vaccination. For subjects who test positive on ELISA due to vaccination, they will be invited annually for HIV antibody testing until the antibodies turn negative. To reduce potential social discrimination in education, employment, healthcare, international travel, and other areas, NCAIDS will issue a proof card confirming participation in the HIV vaccine clinical trial. The card will include the contact information of the relevant sponsor representative and bear the official seal of the National Center for AIDS/STD Control and Prevention, China CDC, for use when needed.

5.6.2 HIV Infection During the Trial

If a subject tests positive for HIV antibodies or nucleic acids during the trial due to reasons unrelated to vaccination, the following measures will be taken:
(1) Discontinue the subject's vaccination schedule.
(2) Refer the subject to a medical institution for further care, based on their willing.
(3) Provide counseling by clinical trial researchers on the following issues:

- Psychological and social issues related to HIV infection.
- Partner-related concerns.
- Measures to avoid infecting others.

(4) Conduct immunological follow-up for subjects who become HIV-infected during the trial. The content and frequency of follow-up will be determined by the trial management committee based on the vaccination schedule.

5.7 Data Management

The data management center for this study is the Health Statistics Office of the Chinese Center for Disease Control and Prevention.

5.7.1 Data Management System

The data management system used in this study is DataFax 3.7, specifically designed for clinical trial data management.

The DataFax system is a computer-based data management system that includes data transmission, database creation, data entry, data quality control, and data reporting. Its primary working principle involves transmitting scanned images of survey forms from the research site to the data center via fax or the internet. The DataFax system at the data center converts these images into data using ICR (Intelligent Character Recognition) and inputs them into the study project database, while saving all fax images on disk. The DataFax system reviews the data through pre-set quality control procedures and identifies issues, which are then reported back to the clinical research unit via fax or the internet in the form of quality reports. Clinical researchers can immediately confirm and correct any issues, and promptly provide feedback to the data center.

The schematic diagram of the DataFax system is as follows:

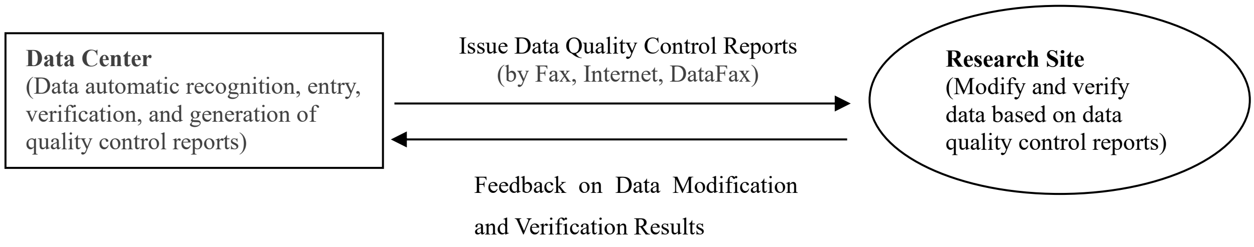

Figure 2. Schematic Diagram of the DataFax System

5.7.2 Completion and Modification of CRF Forms

Clinical researchers are responsible for completing the CRF forms for each enrolled subject. After review by data quality control personnel, the forms are transmitted to the DataFax system at the data center via fax or the internet.

If there are any questions regarding the CRF forms, the data manager will generate a Data Query Form (DRQ) to inquire with the clinical researchers. Researchers should respond promptly, make corrections on the original report form, and then fax the updated form back to

the data center.

5.7.3 Data Locking

After completing the collection of clinical and laboratory data and ensuring that the database meets the locking criteria, the clinical research unit, statistical analysts, and sponsor will lock the data. Once locked, the data files will no longer be modified. Any data errors discovered after locking will be confirmed and corrected in the statistical analysis program.

5.8 Statistical Analysis

5.8.1 Study Indicators

(1) Safety Indicators:

Incidence rates of various adverse events; Abnormal changes in vital signs, physical examinations, blood tests, blood biochemistry, electrocardiograms, and other laboratory test results.

(2) Immunogenicity Indicators:

Positive rates of humoral and cellular immune responses related to vaccination.

5.8.2 Sample Size

According to the "Technical Guidelines for Clinical Research of HIV Vaccines" issued by the State Food and Drug Administration, Phase I clinical trials of HIV vaccines should include 20-30 HIV-negative healthy volunteers.

As required by the clinical trial approval from the China Food and Drug Administration, this Phase Ia study will enroll 12 healthy volunteers to receive the rTV vaccine.

5.8.3 Randomization of Subjects

To avoid subjective selection bias by researchers and severe imbalance in sample allocation, a block randomization method will be used in this trial. The randomization table will be generated using SAS 9.1.2 software by statisticians from the Health Statistics Office of the Chinese Center for Disease Control and Prevention.

To ensure the safety of all subjects, the high-dose group will only begin vaccination after a 2-week observation period following the completion of the low-dose group. Therefore, Phase Ia subjects will not be randomized but will be divided into two groups based on prior smallpox vaccination history. Subjects will be assigned to the low-dose or high-dose group in

the order of enrollment, with 3 subjects in each group.

5.8.4 Blinding Design

Due to the noticeable differences in local reactions among subjects receiving different doses of the rTV vaccine in Phase Ia, blinding will not be implemented in this phase.

5.8.5 Statistical Analysis Content and Methods

(1) Subject Enrollment and Trial Completion Status.

(2) Baseline Data Analysis of Enrolled Subjects:

Continuous variables will be described using mean, median, standard deviation, maximum, minimum, and interquartile range. Categorical data will be described using frequency, percentage, cumulative frequency, and cumulative percentage.

(3) Safety and Tolerability Evaluation:

In the safety evaluation, descriptive analysis will be used for vital signs (blood pressure, pulse, temperature, respiration), physical examinations, and laboratory tests (blood tests, blood biochemistry) at each follow-up period. Statistical comparisons of measurement indicators across different follow-up periods will be conducted using repeated measures ANOVA. Electrocardiogram (ECG) results will list the status at each follow-up period and changes before and after the trial, along with a list of participants with abnormal results.

Descriptive analysis will be conducted for all adverse events and adverse reactions (definitely related, possibly related, or undetermined related to vaccination). For adverse events and reactions with higher incidence rates, chi-square tests will be used for statistical comparison. For those with very low incidence rates, Poisson distribution-based statistical tests will be applied.

Subjects included in the safety analysis must meet the following criteria:

- Meet all inclusion and exclusion criteria.
- Have received at least one dose of the vaccine.

(4) Immunogenicity Evaluation:

Descriptive analysis will be used to report the positive rates of humoral and cellular immune responses.

(5) Exploratory Analysis:

Certain characteristics of subjects, such as gender and ethnicity, may influence study outcomes. Identifying factors affecting vaccine safety and immunogenicity is a key focus of exploratory analysis.

5.8.6 Hypothesis Testing and Significance Level

All statistical tests will be conducted at a 5% significance level. P-values will be provided for all hypothesis tests.

5.8.7 Handling of Missing Data

In statistical analysis, missing data will not be estimated. Unless otherwise specified, all results are based on available data.

5.8.8 Statistical Analysis Software

Unless otherwise stated, all analyses in this report were generated using the licensed SAS 9.1.3 software (Site ID: 47174001).

5.9 Data Management

5.9.1 Data Management at Clinical Trial Sites

Clinical trial sites are responsible for maintaining detailed medical records of subjects, including all laboratory test reports, with subject ID, specimen collection date, and examiner's signature. If abnormal results are found, actions taken, report review date, and reviewing physician's signature must be noted. Any modifications to test results require the researcher's signature and date.

Researchers must accurately complete CRF forms and transmit them to the clinical data management center. Upon receiving data quality control reports from the data center, researchers should promptly address queries, make corrections on the original report forms, and fax them back to the data center.

According to the "Good Clinical Practice for Drugs" issued by the State Food and Drug Administration, clinical trial sites must securely store all research materials, including subject medical records, original laboratory reports, signed informed consent forms, CRFs, and detailed treatment records, for 5 years after the trial ends. A list of materials to be retained is provided in Annex 7.

5.9.2 Data Management by the Sponsor

According to the "Good Clinical Practice for Drugs," the NCAIDS clinical trial management core will securely store all materials related to the trial application, approval, execution, and completion (details in Annex 7) for 5 years after product approval or termination of development.

5.9.3 Ownership of Trial Data

All data from this clinical trial belong to the National Center for AIDS/STD Control and Prevention, China CDC, and the Beijing Institute of Biological Products. Without written consent from the sponsor, researchers may not provide any data to third parties, except as required by the State Food and Drug Administration.

5.10 Confidentiality and Ethics

The clinical trial will strictly adhere to medical confidentiality principles, following the guidelines of GCP and the Helsinki Declaration (2000). The clinical research protocol, subject information sheets, and informed consent forms must be approved by the NCAIDS and the Ethics Review Committee of PUMCH.

To protect subjects, interviews with volunteers will be conducted in private, and information about their participation must not be disclosed to unrelated personnel. All laboratory specimens, CRFs, examination reports, and other records will be identified by numerical codes and stored securely under专人保管. No personal information will be published without the subject's consent.

6 Quality Assurance and Quality Control in Clinical Trials

Both the sponsor and clinical trial researchers must fulfill their respective responsibilities, strictly follow the clinical trial protocol, and implement standard operating procedures to ensure the quality control and quality assurance system of the trial. All observations and findings must be verified, and quality control must be applied at every stage of data processing to ensure data integrity, accuracy, and reliability.

6.1 Data Safety Monitoring Board (DSMB)

An independent DSMB, comprising renowned statisticians, clinical experts, vaccinologists, and ethicists, will oversee the study. The DSMB will review the study protocol, monitor protocol adherence, regularly review safety data, and audit trial phases and final reports to protect subject interests and ensure scientific rigor. The DSMB will meet every two months to discuss data reports, guide the determination of serious adverse events related to the vaccine,

and provide recommendations to the sponsor regarding protocol modifications or trial termination. Written opinions and recommendations will be submitted to the sponsor after each meeting.

6.2 Quality Assurance in Clinical Trials

Before the trial begins, standard operating procedures (SOPs) must be established for trial management, subject recruitment, screening, enrollment, follow-up, adverse event recording and reporting, specimen collection and management, laboratory safety and operations, vaccine transportation, usage, storage, and data management. Clinical trial sites must strictly adhere to the protocol and SOPs, with designated quality assurance personnel overseeing all trial activities, including informed consent, CRFs, adverse event reports, and other records. Quality assurance personnel must maintain daily trial logs, documenting all activities, data completeness and accuracy, responses to data center queries, and any unresolved issues.

6.3 Clinical Trial Monitoring

NCAIDS will establish a quality control and assurance system, appoint trained monitors acceptable to researchers, and report trial progress and data verification to the sponsor. Monitoring activities include:

(1) Confirming that trial sites have adequate conditions, including trained personnel, functioning laboratory equipment, necessary examination facilities, and sufficient subject enrollment.

(2) Ensuring protocol adherence, verifying informed consent, monitoring enrollment rates and progress, and confirming subject eligibility.

(3) Verifying the accuracy and completeness of all data and CRFs, ensuring consistency with source documents, and documenting corrections, researcher signatures, and dates.

(4) Ensuring all adverse events are recorded, and serious adverse events are reported and documented within the required timeframe.

(5) Verifying vaccine usage records, including quantity, shipment, receipt, storage, and disposal of unused vaccine, and ensuring compliance with the trial protocol.

(6) Assisting trial site personnel in reporting data and results to the sponsor.

(7) Submitting written monitoring reports to the sponsor after each visit, detailing findings and observations.

6.4 Clinical Trial Audits and Inspections

Depending on trial progress, the sponsor or regulatory authorities may appoint auditors to

systematically review trial activities and documents to evaluate compliance with the protocol, SOPs, and regulations. Audits will be conducted by individuals not directly involved in the trial. The sponsor and trial sites may also undergo inspections by regulatory authorities to assess task execution and review relevant documents and facilities.

Annex 1. Informed Consent Form for Participation in a Phase Ia Clinical Trial of AIDS vaccine (Nucleic Acid in Combination with Recombinant Vaccinia Tiantan)

I. Information About the Phase Ia Clinical Trial

Study Title: Phase I Clinical Trial to Evaluate the Safety and Immunogenicity of the Recombinant Vaccinia Tiantan (rTV) AIDS Vaccine in Healthy Volunteers

Project leader：

Principal investigator: Li Taisheng, Peking Union Medical College Hospital
Team leader of vaccine providing unit:

YimingShao, National Center for AIDS/STD Control and Prevention, China CDC;

Jing Xu , Beijing Institute of Biological Products

You are invited to participate in a clinical research study. Before deciding whether to participate, it is important for you to understand the details of the study and the potential benefits and risks involved. This process is called informed consent. This informed consent form provides information about the study and its potential impact on your health and life, helping you make an informed decision.

Some terms or information in this form may be unfamiliar to you. Study staff will explain any unclear content. Please feel free to ask questions to ensure you fully understand the study and why you are being invited to participate.

Thank you for taking the time to read this information.

Why is HIV Vaccine Research Being Conducted?

Since the first case was identified in 1981, HIV/AIDS has spread rapidly worldwide, becoming one of the most severe viral diseases affecting human health. By the end of 2006, there were approximately 39.5 million people living with HIV globally, with 4.3 million new infections and 2.9 million AIDS-related deaths in that year alone. In China, after periods of sporadic and localized outbreaks, the HIV epidemic entered a rapid growth phase in the late 1990s. By January 2006, China had an estimated 650,000 HIV-infected individuals, including 75,000 AIDS patients.

The global HIV/AIDS epidemic urgently necessitates the development of safe, effective, and affordable HIV vaccines. The goal of HIV vaccine research is to prevent HIV infection or delay the onset of AIDS/reduce viral load in patients.

What is a Clinical Trial?

A clinical trial, also known as clinical research, involves systematic studies of new drugs or vaccines in humans (healthy volunteers or patients) before they can be marketed. These studies aim to determine the safety, side effects, and effectiveness of the new drug or vaccine in preventing or treating diseases. Any vaccine must undergo scientifically designed clinical trials to evaluate its safety, immunogenicity, and protective efficacy.

Do Vaccine Clinical Trials Require Regulatory Approval?

Not all vaccines can undergo clinical trials. Extensive preclinical research must demonstrate that the vaccine is likely to be safe and effective in humans. The vaccine must undergo quality testing and safety evaluation in animals at designated national institutions, and all preclinical data must pass technical review by the State Food and Drug Administration (SFDA). Clinical trials can only begin after receiving approval from the SFDA.

To protect the safety and rights of trial participants, the clinical trial protocol must be reviewed and approved by the ethics review committee of the trial site. The committee includes medical professionals, non-medical professionals, legal experts, and external members to ensure impartiality.

What is the Current Status of HIV Vaccine Research Internationally?

The first HIV vaccine clinical trial was conducted in the United States in 1987. By January 2006, 160 HIV vaccine clinical trials had been conducted globally, most of which were Phase I trials (involving dozens of participants), with a few larger Phase II trials (200–300 participants) and only three large-scale Phase III trials (thousands of participants). Almost all trials showed that the experimental HIV vaccines were safe in humans. Although progress has been made, the efficacy of these vaccines in preventing HIV infection remains limited, and scientists continue to develop new vaccines.

What is the Product and Vaccination Method in This Clinical Trial?

The product in this Phase I clinical trial is a combination HIV vaccine consisting of two components: an HIV-1 DNA vaccine and a recombinant poxvirus (rTV) HIV vaccine. This vaccine received approval from the SFDA in November 2006 to proceed with Phase I clinical

trials. The trial is divided into Phase Ia and Phase Ib, involving a total of 48 participants and lasting approximately 58 weeks. You are being invited to participate in Phase Ia.

Phase Ia requires 12 participants, including those who have or have not previously received the smallpox vaccine, and lasts 26 weeks. Participants will be divided into groups to receive two different doses of the rTV vaccine, and you may be assigned to either dose group.

The rTV HIV vaccine uses the Tian Tan strain of the poxvirus as a vector to carry HIV gene fragments. The Tian Tan strain was widely used as a smallpox vaccine in China, effectively preventing smallpox and contributing to its eradication in the country. The vaccine will be administered using a bifurcated needle on the outer upper arm.

What Are the Reactions After Vaccination?

(1) **Reactions to the rTV Vaccine**:

Since this is the first clinical trial of the rTV vaccine, its specific reactions will only be known after the trial concludes. However, because the vaccine uses the Tian Tan strain as a vector, reactions observed during its use as a smallpox vaccine can serve as a reference. The insertion of HIV gene fragments has significantly reduced the virulence of the Tian Tan strain. Animal studies show that the rTV vaccine is 100–1000 times less toxic to mice than the Tian Tan strain, suggesting that adverse reactions to this HIV vaccine should be much milder than those of the Tian Tan smallpox vaccine.

Reactions to the Tian Tan smallpox vaccine can be divided into common and rare adverse reactions. Common reactions are normal responses to successful vaccination and may include pain at the injection site, followed by papules, vesicles, pustules, scabbing, and permanent scarring (if you have previously received the smallpox vaccine, you may have such a scar on your arm). These skin reactions typically last 2–3 weeks. You may also experience fever, myalgia, chills, rash outside the injection site, nausea, headache, fatigue, and swollen lymph nodes, but these symptoms usually resolve within a few days without medication.

In rare cases, severe adverse reactions may occur after receiving the Tian Tan smallpox vaccine, with an incidence of approximately 5.6 per million. However, over 80% of these reactions occur in infants, the elderly, those with a history of eczema, or immunocompromised individuals. These reactions include generalized vaccinia, progressive vaccinia, eczema vaccinatum, and post-vaccinial encephalitis, which are generally treatable. Additionally, if you have close contacts who are infants, pregnant women, or

immunocompromised (e.g., due to HIV or cancer), improper handling of the vaccination site could lead to vaccinia virus infection in them.

During screening, researchers will assess your personal history, conduct physical examinations, and perform laboratory tests to determine your suitability for the rTV vaccine. They will also guide you on personal precautions to minimize severe adverse reactions and prevent vaccinia virus infection in your contacts.

(2) **Other Vaccination Reactions**:
As with other vaccines, in rare cases, you may experience fainting (vasovagal syncope) due to fasting, fatigue, nervousness, or fear. If you are allergic to any vaccine components, you may experience symptoms such as chest tightness, shortness of breath, palpitations, difficulty breathing, pallor, cyanosis, bradycardia, hypotension, cold extremities, convulsions, or coma shortly after vaccination. These symptoms can usually be managed with appropriate treatment.

(3) **HIV Testing After Vaccination**:
The HIV vaccine itself will not cause HIV infection. If you test positive for HIV, there are two possibilities: a vaccine-induced positive result or actual HIV infection. The National Center for AIDS/STD Control and Prevention will conduct additional nucleic acid testing to distinguish between true HIV infection and vaccine-induced responses.

> **Vaccine-Induced HIV Antibody Positivity**: The HIV vaccine may stimulate an immune response, leading to positive results in routine HIV tests. If this occurs, researchers will invite you for annual HIV antibody testing until the results turn negative. To reduce potential social discrimination in education, employment, and healthcare, we will provide you with a proof card confirming your participation in the HIV vaccine trial. The card will include contact information for the study lead and the official seal of the National Center for AIDS/STD Control and Prevention for your use as needed. We recommend that you avoid donating blood until your routine HIV test results turn negative.
>
> **HIV Infection**: If you are found to be HIV-infected during the trial, researchers will discontinue your vaccination and arrange referral based on your preference. Experts from PUMCH will provide counseling on psychological, social, partner-related, and risk-reduction issues.

Am I Eligible to Participate?

Phase Ia of the trial requires 12 participants, including both Beijing residents and long-term residents from other regions. Researchers will assess your personal history and test results to determine your eligibility. The criteria for participation are as follows:

You are eligible to participate in the trial if you meet the following criteria:

1. Aged between 18 and 55 years, in good health.
2. Able to attend follow-up visits during the clinical trial period.
3. Able to understand and agree to the content of the informed consent form.
4. At low risk of HIV infection and willing to maintain this low-risk status during the study period:

- No history of intravenous drug use.
- No diagnosis of gonorrhea or syphilis in the past year.
- No current or past high-risk sexual partners (e.g., intravenous drug users or HIV-positive partners).
- No unprotected sex with non-regular sexual partners in the past year.

5. Agree to undergo approximately 9 venous blood draws and allow blood samples to be stored.
6. Willing to undergo HIV and syphilis blood tests.
7. Willing to use effective contraception (including combined oral contraceptives, injectable contraceptives, intrauterine devices, or consistent condom use) with your sexual partner from 2 weeks before vaccination until 6 months after vaccination; If you are a female volunteer, you must be willing to undergo urine pregnancy tests before vaccination and during follow-up visits.

You cannot participate in the trial if you meet any of the following conditions:

1. Pregnancy, breastfeeding, or planning to become pregnant during the trial, or having close contacts who are pregnant or breastfeeding within 1 month of vaccination.
2. Presence of the following diseases or medical history:

- Congenital or acquired immunodeficiency diseases such as AIDS, or having close contacts with such patients within 1 month of vaccination.
- Undergoing treatments that affect immune responses, such as intravenous or intramuscular corticosteroids or oral prednisone for more than 2 weeks, or using immunosuppressants like alkylating agents, antimetabolites, or radiation therapy; or having close contacts undergoing such treatments within 1 month of vaccination.
- Immunosuppressive diseases such as malignant tumors, organ or stem cell transplantation, or agammaglobulinemia; or having close contacts with such patients within 1 month of vaccination.
- History or current diagnosis of eczema or atopic dermatitis; current skin conditions causing skin damage, such as burns, scalds, chickenpox, impetigo, shingles, or psoriasis; or having close contacts with such patients within 1 month of vaccination.
- History or current diagnosis of hypertension, heart disease, diabetes, thyroid disease, asthma, angioedema, asplenia, mental or psychological disorders, epilepsy, etc.
- Diseases requiring frequent injections or blood draws.
- History of fainting or allergic reactions after vaccination.
- Current acute infectious diseases or febrile illnesses.

3. Presence of the following situations:

- Received a live attenuated vaccine within the last 2 months or any other vaccine within the last 2 weeks.
- Received immunoglobulin or other blood products within the last 4 months.
- Participated in another medical product trial within the last 1 month.
- Drug abuse, substance dependence, alcoholism, or heavy smoking.

4. Abnormal laboratory test results:

- HIV antibody positive or indeterminate.
- Hepatitis B surface antigen or hepatitis C antibody positive, or serological evidence of active syphilis.
- Abnormal liver function, kidney function, cardiac enzymes, blood glucose, etc.
- Abnormal humoral or cellular immune function.
- Other significant abnormalities in physical examination or laboratory tests that the physician deems unsuitable for participation.

5. Inability to comply with the study protocol or provide informed consent due to medical, psychological, social, occupational, or other reasons.

Am I Required to Participate in the Trial?

After carefully reading this information, you can decide whether to participate in the trial. If you decide to participate, please sign this informed consent form. During the trial, you may withdraw from the study at any time without providing a reason. Your decision not to participate or to withdraw will not affect your medical treatment at PUMCH.

What Do I Need to Do If I Decide to Participate?

First, you will come to the hospital for a screening visit. All your questions will be answered at that time. Researchers will determine whether you are eligible to participate. They will ask you personal questions, including those related to HIV risk behaviors, and provide you with a free physical examination and blood tests, including an HIV test. If your personal and medical conditions are suitable for participation, you will be randomly assigned to a study group after signing the informed consent form, and vaccination will begin.

After vaccination, you will be observed for 24 hours. Researchers will closely monitor your local and systemic reactions. They will then provide you with a daily diary card and explain how to fill it out to record post-vaccination reactions. You will complete the diary card daily for 4 weeks after vaccination and submit it to the researchers during each follow-up visit. Any discomfort experienced outside the hospital should be recorded on the diary card. If you experience symptoms of grade 3 or higher as listed on the diary card, you should contact medical staff as soon as possible. If you agree, researchers will meet with you every other day to observe post-vaccination reactions until the scab at the vaccination site falls off (approximately 2–3 weeks). Follow-up visits will occur at PUMCH on days 3, 1 week, 2 weeks, 4 weeks, 8 weeks, 16 weeks, and 24 weeks after vaccination. Therefore, you will need to visit the hospital at least 9 times for follow-up and venous blood draws. The maximum blood volume drawn each time will not exceed 43 ml. Your blood will be used directly for testing, but a small portion will be stored for future analysis, such as HLA typing, to accurately evaluate the immune response induced by the vaccine.

As a participant, you will also need to do the following:

- Maintain a low-risk status for HIV infection.
- You and your spouse should avoid pregnancy for 6 months after vaccination.
- Avoid contact with the following individuals for 1 month after receiving the rTV vaccine: pregnant women, immunocompromised individuals (e.g., those with AIDS, leukemia, other cancers, organ transplants, lupus, etc.), individuals with eczema or skin lesions, and infants under 1 year old.
- Due to individual differences, the expected skin reaction at the vaccination site may not occur, and there may be no significant change in serum vaccinia virus antibodies compared to pre-vaccination levels, indicating unsuccessful vaccination. In such cases, researchers will administer a second dose on the opposite upper arm 2 weeks after the first vaccination, using the same dose as the first.
- Avoid touching or scratching the vaccination site until the scab falls off. If you accidentally touch the site, wash your hands with soap immediately and avoid rubbing your eyes. Please cooperate with medical staff to cover the vaccination site with breathable gauze or bandages until the scab falls off. Wear loose-fitting long-sleeved clothing until the scab falls off.

- Adhering to the follow-up schedule is crucial for the trial results. Please attend follow-up visits as arranged by the researchers. If you cannot attend a scheduled visit, please contact the researchers to arrange an alternative time.
- Since this is the first trial of this vaccine, we will monitor your health status over an extended period. We will contact you annually. If your contact information changes, please keep us updated.

As compensation for your travel expenses and the inconvenience of frequent testing, we will provide you with 5,000 yuan at the end of the trial.

What Happens If I Experience Issues During the Trial?

If you experience adverse reactions due to vaccination during the trial, the vaccine development unit will cover your medical expenses and provide appropriate financial compensation for severe adverse reactions. For illnesses unrelated to vaccination or medical procedures, researchers will assist in arranging your treatment, but you will need to cover the costs. If you do not have accidental injury insurance, the vaccine development unit will provide such insurance during the trial. If you experience psychological or social issues during the trial, you can consult the researchers. If you have any concerns about our staff, please contact the project lead directly.

Is My Participation Confidential?

Yes. Your name and personal information will only be collected during volunteer registration and when signing the informed consent form. These records will be securely stored by designated researchers and will not be disclosed to anyone unrelated to the study. During screening and laboratory tests, you will be assigned a participant number. All subsequent trial records will use this number for identification. Interviews with researchers will be conducted in private settings. Your personal information will not be disclosed without your consent.

How Will the Trial Results Be Handled?

The trial results will be summarized in scientific papers and submitted to medical journals or presented at scientific conferences. However, your personal information will not be disclosed.

The potential benefits of participating in the trial for you:

• You will undergo a full body check-up before entering the clinical trial and after the trial ends, providing an opportunity to assess your health status.

• You will receive information and preventive knowledge about sexually transmitted diseases, including AIDS.

• The information obtained from the trial will help scientists develop an effective AIDS vaccine, which could benefit hundreds of millions of people around the world who are under the threat of AIDS.
**The potential inconveniences and risks of participating in the trial for you:**<br>• You will need to visit the hospital multiple times for follow-ups, which may cause inconvenience to your work and life.<br>• Vaccination may cause you physical discomfort. Although the incidence is very low, there is still a risk of more serious adverse reactions after vaccination.<br>• For 1 month after receiving the vaccinia virus vaccine, your activities and contact with people will be somewhat restricted.<br>• If your blood tests positive for HIV antibodies due to vaccination, you may face some social discrimination.<br>• You might mistakenly believe that after vaccination, you are resistant to HIV infection and therefore can engage in high-risk behaviors. This is a misconception, as it cannot be guaranteed that the vaccine has a protective effect before the clinical trial is completed.<br>• After participating in this trial, you will not be able to participate in any clinical trials for AIDS vaccines.

If You Would Like More Information, Please Contact:

National Center for AIDS/STD Control and Prevention, Chinese Center for Disease Control and Prevention

Contact Person: ______________________

Phone: ___________________

Department of Infectious Diseases, Peking Union Medical College Hospital

Contact Person: _________________________

Phone: ___________________

Ethics Committee for Clinical Pharmacology Trials, Peking Union Medical College Hospital

Contact Person: _________________________________

Phone: _________________________________

Please keep a copy of this information sheet. If you are willing to participate in this clinical trial, please sign this information sheet and read and sign the informed consent form for screening.

Volunteer Name: __________ Signature: ______________ Date: _____________

Witness Name: __________ Signature: ______________ Date: _____________

Investigator Name: __________ Signature: ______________ Date: _____________

II. Informed Consent Form

Part I: Informed Consent for Screening to Participate in the Phase I Clinical Trial of the AIDS Vaccine

Screening Number: ______________________ Date of Birth: ____ Year ____ Month ____ Day

The subject must personally complete all the following questions.

Please select your answer:

Have you received the information sheet for the Phase I clinical trial of the AIDS vaccine?
Yes No

Do you understand the purpose of the AIDS vaccine clinical trial?
Yes No

Are you aware of the product and trial plan for this vaccine clinical trial?
Yes No

Have you had the opportunity to ask questions or participate in discussions about this trial?
Yes No

Have all your questions been answered satisfactorily?
Yes No

Are you fully aware that you will need to undergo HIV testing before and during the trial to participate?
Yes No

Do you understand that you and your spouse must use contraception from 2 weeks before the first vaccination until 6 months after the last vaccination?
Yes No

Please fill in the name of the researcher who discussed the trial with you:

______________________________ ______________________________

Do you understand that you can withdraw from this trial at any time without providing a reason, and this will not affect your future medical care?

Yes No

Do you agree to participate in the screening as a candidate for this trial?

Yes No

Volunteer Name: __________ Signature: ______________ Date: _____________

Witness Name: __________ Signature: ______________ Date: _____________

Investigator Name: __________ Signature: ______________ Date: _____________

Part II: Informed Consent to Participate in the Phase I Clinical Trial of the AIDS Vaccine

Screening Number: ________________ Date of Birth: ____ Year ____ Month ____ Day

The subject must personally complete all the following questions.

Please select your answer:

Have you received sufficient information about this study?

Yes No

Do you understand that it is uncertain whether the tested vaccine can prevent HIV infection?

Yes No

Do you agree to retain your samples for future immunological experiments related to HIV vaccines?

Yes No

Are you aware of the potential severe adverse reactions after vaccination?

Yes No

Do you expect to have close contacts within 1 month after receiving the recombinant poxvirus vaccine who are immunocompromised (e.g., AIDS, leukemia, cancer, organ transplant, lupus, immunodeficiency diseases) or undergoing treatments that may reduce immune function (e.g., hormones, chemotherapy, immunosuppressants)?

Yes No

Do you expect to have close contacts within 1 month after receiving the recombinant poxvirus vaccine who are infants under 1 year old, pregnant women, or individuals with

eczema or other skin diseases?

Yes No

Do you agree to participate in this trial?

Yes No

Are you willing to undergo long-term follow-up and regular contact?

Yes No

If you are participating in Phase Ia, do you agree to allow medical staff to visit you every other day for 2–3 weeks after receiving the rTV vaccine?

Yes No

Are you aware that you need to sign two copies of the informed consent form, one for your own records and one to be kept by the research unit?

Yes No

Volunteer Name: __________ Signature: ______________ Date: _____________

Witness Name: __________ Signature: ______________ Date: _____________

Investigator Name: __________ Signature: ______________ Date: _____________

Annex 2. Grading of Clinical and Laboratory Adverse Events

Parameter	Mild (Grade 1)	Moderate (Grade 2)	Severe (Grade 3)	Potentially Life-Threatening (Grade 4)
**Laboratory Tests**				
**Hematology**				
Hemoglobin (g/L)	95–105	80–94	65–79	<65
Leukocytosis (10^9/L)	13.1–15	15.1–20	20.1–30	>30
Leukopenia (10^9/L)	2.5–3.5	1.5–2.49	1.0–1.49	<1.0
Thrombocytopenia (10^9/L)	75–99	50–74	25–49	<25
**Blood Chemistry**				
Liver Function – ALT, AST	1.25–2.5×ULN*	2.6–5×ULN	5.1–10×ULN	>10×ULN
Creatinine	1.1–1.5×ULN	1.6–3.0×ULN	3.1–6×ULN	>6×ULN
BUN	1.25–2.5×ULN	2.6–5×ULN	5.1–10×ULN	>10×ULN
Bilirubin: Elevated due to factors with normal liver function	1.1～1.5×ULN	1.6～2.0×ULN	2.0～3.0×ULN	＞3.0×ULN
Bilirubin: Elevated due to factors with abnormal liver function	1.1～1.25×ULN	1.26～1.5×ULN	1.51～1.75×ULN	＞1.75×ULN
CK and Isoenzymes	1.25～1.5×ULN	1.6～3.0×ULN	3.1～10×ULN	＞10×ULN
Troponin	1.25～1.5×ULN	1.6～3.0×ULN	3.1～10×ULN	＞10×ULN
**Urine**				
Glucose (mmol/L)	≤15	15.1～20	20.1～30	＞30
Protein (g/L)	0.16～0.3	0.31～0.75	0.76～1.0	＞1.0
Red Blood Cells (cells/μL)	25～80	81～200	＞200	Gross hematuria
**Local Reactions and Skin Manifestations**				
Injection Site Pain	Mild pain, no treatment or occasional non-prescription	Moderate pain, regular non-prescription medication or	Severe pain, repeated prescription medication required	Hospitalization required

		medication required	occasional prescription medication required		
**DNA Vaccine**	Injection Site Erythema	Diameter <1.5 cm	Diameter 1.5–3 cm	Diameter >3 cm	Gangrene or exfoliative dermatitis
	Injection Site Blisters and Ulcers	Blisters or ulcers at injection site, diameter <1 cm	Blisters or ulcers at injection site, diameter 1–2 cm, heals within 2 weeks	Blisters or ulcers at injection site, does not heal for over 2 weeks	Necrosis
	Injection Site Induration	Diameter <1.5 cm	Diameter 1.5–3 cm	Diameter >3 cm	Gangrene or exfoliative dermatitis
	Injection Site Papules	Diameter <1.5 cm	Diameter 1.5–3 cm	Diameter >3 cm	
	Injection Site Pustules	Diameter <0.5 cm	Diameter 0.5–1 cm	Diameter >1 cm, no tendency to heal for 2 weeks	Necrosi
	Injection Site Swelling	Diameter <1.5 cm, no impact on activity	Diameter 1.5–3 cm or impacts activity	Diameter >3 cm or restricts daily activities	Gangrene
	Generalized Rash	Scattered maculopapular rash (<5 areas), no itching or other symptoms	Scattered macules or papules (>5 areas), itching or other symptoms, no treatment required	Diffuse macules or papules, or exudative rash, requires medication	Rash involving mucous membranes, exfoliative dermatitis, or erythema multiforme, or Stevens-Johnson syndrome
**rTV vaccine**	Injection Site Papules	Diameter 1.1–1.5 cm	Diameter 1.6–3 cm	Diameter >3 cm	
	Injection Site Swelling or Redness	Diameter 3–5 cm	Diameter 5.1–10 cm, improves within 5 days	Diameter 5.1–10 cm, persists for over 5 days, or diameter >10 cm	
	Injection Site Induration	Diameter 1.1–1.5 cm	Diameter 1.6–3 cm	Diameter >3 cm	
	Injection Site Blisters	Diameter 1.1–1.5 cm	Diameter 1.6–3 cm	Diameter >3 cm	

	Injection Site Pustules	Diameter 1.1–1.5 cm	Diameter 1.6–3 cm	Diameter >3 cm	Pustules continue to expand without scabbing after 2 weeks; gangrene or exfoliative dermatitis
	Injection Site Erythema	Diameter <1.5 cm	Diameter 1.5–3 cm	Diameter >3 cm	Gangrene or exfoliative dermatitis
**Systemic Symptoms**					
Headache		Does not affect activity, no treatment required	Transient, affects daily activities, requires treatment (non-narcotic analgesics)	Severely affects daily activities, occasional narcotic treatment required	Persistent, requires repeated narcotic treatment
Chills		Occasional OTC non-steroidal antipyretics required	Limits daily activities >6 hours, or requires repeated non-steroidal antipyretics	Severely affects daily activities, requires prescription medication	Hospitalization required
Fatigue		Reduced normal activity <48 hours	Reduced normal activity 20%–50% >48 hours	Severely affects daily activities, reduces normal activity >50%, unable to work	Unable to care for self, emergency or hospitalization
Myalgia		Does not affect daily activities	Tenderness in non-injection site muscles, slightly limits daily activities	Severe muscle tenderness, severely affects daily activities	Severe symptoms, muscle necrosis, emergency or hospitalization
Arthralgia		Mild pain, does not limit daily activities	Moderate pain, slightly limits daily activities	Severe pain, severely affects daily activities	Hospitalization required
Nausea		Mild or transient, does not affect normal eating	Affects normal eating	Unable to eat, requires outpatient IV fluids	Hospitalization required
Vomiting		1 episode in 24 hours	2–5 episodes in 24 hours	>6 episodes in 24 hours or IV fluids required	Requires hospitalization or alternative nutrition
Diarrhea		Mild or transient, 2–3 episodes/day, lasts <1 week	Moderate or persistent, 4–5 episodes/day, or diarrhea >1 week	>6 episodes/day, or bloody stools, orthostatic hypotension, electrolyte imbalance,	Hypotensive shock, requires hospitalization

			requires >2L IV fluids in 24 hours	
Cough	Transient, no treatment required	Paroxysmal cough, treatment effective	Persistent cough, treatment ineffective	Emergency or hospitalization
Allergic Reaction	Itching without rash	Localized urticaria	Generalized urticaria, angioedema	Severe allergic reaction, or post-vaccination encephalitis
Lymphadenopathy	Newly appeared ipsilateral axillary lymphadenopathy, multiple nodes possible, largest single node diameter ≤2 cm; may be tender but does not affect daily activities	Newly appeared ipsilateral axillary lymphadenopathy, plus one of the following: ① New lymphadenopathy in 1–2 other lymph node regions; ② Largest single node diameter 2–3 cm; ③ Slightly limits daily activities	Newly appeared ipsilateral axillary lymphadenopathy, plus one of the following: ① New lymphadenopathy in ≥3 other lymph node regions; ② Largest single node diameter >3 cm; ③ Severe tenderness, severely affects daily activities	—
Other Discomfort	Does not affect daily activities	Slightly affects daily activities, no medication required	Severely affects daily activities, requires medication	Requires hospitalization
**Vital Signs***				
Fever, Axillary Temperature	37.3–37.9°C	38.0–39.0°C	>39.0°C	
Tachycardia (beats/min)	101–115	116–130	>130, or other rapid arrhythmias	Emergency or hospitalization due to arrhythmia
Bradycardia (beats/min)	50–54	45–49	<45	Emergency or hospitalization due to arrhythmia
Hypertension (Systolic BP, mmHg)**	141–150	151–155	>155	Emergency or hospitalization due to severe hypertension
Hypertension (Diastolic BP, mmHg)**	91–95	96–100	>100	Emergency or hospitalization due to severe hypertension
Hypotension (Systolic BP, mmHg)**	85–89	80–84	<80	Emergency or hospitalization due to hypotensive

				shock
Hypertension (Diastolic BP, mmHg)**	91–95	96–100	>100	Emergency or hospitalization due to severe hypertension

Note： ULN: Upper Limit of Normal

* Cited from the “Chinese Vaccination Manual”, subjects should be tested in a resting state.

** The determination of abnormal blood pressure should be compared with the baseline blood pressure before vaccine administration for specific analysis.

Annex 3. Subject Diary Card

Notes for completing this card:

1) Every day, complete the diary card within 2 weeks after DNA vaccination and 4 weeks after rTV vaccination, and record any discomforts occurring at other times on the dairy card.
2) Please record the non-prescription drugs or other treatments to alleviate local reactions;
3) If you have any symptoms that cannot go away by themselves, please contact the study staff;
4) After you have completed the diary card on the day, please give the completed dairy to the study staff at your next visit.
5) For safety, after rTV vaccination, subjects themselves may not remove the gauze covering the vaccination site without special circumstances

Diary Card

Screening number □□□□□□ Subject initials □□□□

Recording date: 20 Year Month Day Day after vaccination: (day 1-28)

Axillary temperature:

A.M. (6:00- 8:00) ℃ P.M. (8:00- 10:00) ℃

Have you participated in sports or heavy physical labor? Yes No

Type of sports or heavy physical labor:

Duration: hours

Please record the physical discomfort after vaccination:

Name of symptom	Duration of symptom	Time to relieve

Please record the medications after vaccination:

Drug name	Reason for medication	Time of starting medication	Dosage of medication	Route of administration	Time of ending medication
				1. Oral;<br>2. intramuscular injection;<br>3. Intravenous;<br>4. Topical	
				1. Oral;<br>2. intramuscular injection;<br>3. Intravenous;<br>4. Topical	

Subject's signature: Date：□□□□Year□□Month□□Day

Report form of treatment of systemic and local reactions (including non-prescription drugs)

**NO.**	**Drug name**	**Dosage**	**Times per day**	**Duration of use (from Month Day to Month Day)**	**Reason for using the drug and the effect**
1					
2					
3					
4					
5					
**If other treatments are used, please explain them specifically**					

Annex 4. Summary of Test Results for HIV-1 DNA Vaccine

Test Item	Test Method	Quality Standard	pGP140 (20041123)	PGPNEF (20041124)
**Appearance**	Visual inspection	Clear, colorless liquid, free of foreign matter	Pass	Pass
**pH Value**	*Chinese Pharmacopoeia*	6.7–7.7	Pass	Pass
**Fill Volume**	*Chinese Pharmacopoeia*	Average fill volume not less than the labeled volume (1.0 ml/vial), each vial not less than 93% of the labeled volume	Pass	Pass
**Sterility Test**	*Chinese Pharmacopoeia*	No microbial growth	Pass	Pass
**Identification Test (Restriction Enzyme Mapping)**	Restriction enzyme mapping	Consistent with expected results	Pass	Pass
**Residual E. coli Genomic DNA**	Solid-phase slot blot hybridization	≤2 μg/mg plasmid	Pass	Pass
**Residual Host Cell Protein**	*Chinese Biological Products Regulation*	≤1 μg/mg plasmid	Pass	Pass
**Endotoxin Content**	*Chinese Pharmacopoeia*	≤5 EU/mg plasmid	Pass	Pass
**Residual RNA (Agarose Gel Electrophoresis)**	Agarose gel electrophoresis	No other bands or diffuse nucleic acid bands except plasmid band	Pass	Pass
**Purity Test (UV Absorption)**	UV absorption	A260/A280 ≥ 1.75	Pass	Pass
**Concentration Test (UV Absorption)**	UV absorption	1.8–2.2 mg/ml	Pass	Pass

**Plasmid Conformation Test (Agarose Gel Electrophoresis)**	Agarose gel electrophoresis	Circular plasmid ≥ 90%	Pass	Pass
**Residual LiCl**	ICP-MS	≤1 µg/mg plasmid	Pass	Pass
**In Vitro Gene Expression Test (Western Blot)**	Western Blot	pGP140: Specific band at 130 kDa; pGPNEF: Specific band at 160 kDa	Pass	Pass
**Abnormal Toxicity Test**	*Chinese Biological Products Regulation*	Guinea pig test: Guinea pigs survive and gain weight; Mouse test: Mice survive and gain weight	Pass	Pass
**Humoral Immunogenicity Test (ELISA)**	ED50 ELISA	ED50 ≤ 100 µg (pGP140); ED50 ≤ 80 µg (pGPNEF)	Pass	Pass
**Cellular Immunogenicity Test (ELISPOT)**	ELISPOT	Positive cellular immune response rate in mice vaccinated with DNA vaccine ≥ 60%	Pass	Pass

Annex 5. Summary of Test Results of Recombinant Tiantan Vaccinia (rTV) AIDS Vaccine

Test Item	Test Method	Quality Standard	Results
**Appearance**	Visual inspection	Light brown viscous liquid	Pass
**pH Value**	*Chinese Pharmacopoeia*	7.2 ± 0.5	7.02
**Fill Volume**	*Chinese Pharmacopoeia*	Average fill volume not less than the labeled volume, each vial not less than 93% of the labeled volume	Pass
**Sterility Test**	*Chinese Pharmacopoeia*	Negative	Pass
**Viral Titer**	Hemadsorption method	$5.0 \times 10^6 – 3.0 \times 10^7$ PFU/ml	Pass
**Abnormal Toxicity Test**	*Chinese Biological Products Regulation*	Animals survive for 5 days with no local ulcers	Pass
**Rabbit Virulence Test**	*Chinese Biological Products Regulation* (1979 edition)	Necrosis diameter at 10^{-1} and 10^{-2} dilutions should not exceed 10 mm in total; no necrosis at other dilutions. Local redness or swelling at all 5 dilutions should be less than 70 mm in total.	Pass
**Bacterial Endotoxin**	*Chinese Biological Products Regulation* (2000 edition)	Endotoxin content < 500 EU/ml, < 10 EU per dose	Pass
**Rabbit Virulence Test**	*Chinese Biological Products Regulation* (1979 edition)	Necrosis diameter at 10^{-1} and 10^{-2} dilutions should not exceed 10 mm in total; no necrosis at other dilutions. Local redness or swelling at all 5 dilutions should be less than 70 mm in total.	Pass
**Bacterial Endotoxin**	*Chinese Biological Products Regulation* (2000 edition)	Endotoxin content < 500 EU/ml, < 10 EU per dose	Pass
**HIV Target Gene**	1. PCR<br>2. Western Blotting	1. gagpolΔ fragment should be around 2.9 kb; gp140TM should be around 2.1 kb. gp140TM digested with StuI yields 1170 bp and 975 bp fragments; digested with NdeI yields 176 bp and 1969 bp	Pass

		fragments. gagpolΔ digested with PstI yields 617 bp and 2271 bp fragments; digested with SpeI yields 709 bp and 2079 bp fragments.<br>2. gagpolΔ gene-specific band at 55 kDa, sometimes also 24 kDa and 41 kDa bands. gp140TM gene-specific band at 140 kDa, sometimes degraded bands below 140 kDa.	
**Humoral Immunogenicity Test (ELISA)**	ED50 ELISA	ED50 < 8.0 × 10^6 PFU	Pass
**Cellular Immunogenicity Test**	Intracellular IFN-γ secretion by flow cytometry	Positive if ≥ 50% higher than the mean of the negative control group; vaccination group positive rate ≥ 60%	Pass

Annex 6: Reactions after Smallpox Vaccination and Principles of Treatment

As this is the first clinical study of rTV vaccine with Tiantan strain of vaccinia virus as a vector, the vaccination reactions caused by the vaccine can be determined only after completion of this clinical trial, and the reactions caused by Tiantan strain of vaccinia virus for smallpox vaccination can be regarded as a reference for expected adverse reactions. However, when constructing the rTV vaccine, the insertion of HIV genes has significantly reduced the virulence of Tiantan strain of vaccinia virus. The experiments of animals have also shown that the toxicity of rTV vaccine in mice is 100-1000 times lower than that of Tiantan vaccinia virus, therefore, the adverse reactions caused by AIDS vaccine should be much lower than those caused by Tiantan strain smallpox vaccine.

Reactions in human body after smallpox vaccination include general reaction and abnormal reactions. The general reactions are typical local reactions of smallpox and possibly some systemic symptoms after vaccination. An abnormal reaction refers to a rarely serious adverse reaction after vaccination. Tiantan strain vaccinia virus as a smallpox vaccine has been universally vaccinated in Chinese population, and it has played a good role in the prevention of smallpox epidemic, and successfully eradicates smallpox disease in China. Different from the crude smallpox vaccine from tissues such as cow skin used in other countries, Tiantan strain smallpox vaccine is a refined vaccine obtained by cell culture, therefore, it has lower rate of abnormal reactions after vaccination than other smallpox vaccines, with the incidence of approximately 5.6/million for post-vaccination encephalitis, progressive pox, chicken pox rash, generalized pox and pleomorphic smallpox, and 80% of these reactions occur in infants under 1 year of age and immunocompromised persons. There is no report of deaths after vaccination of cell-cultured Tiantan strain smallpox vaccine manufactured by Beijing Institute of Biological Products Co., Ltd. The general reactions and abnormal reactions developed after smallpox vaccination as well as treatment principles are listed by referring to Compilation of Data from Experience Exchange Meeting on Vaccinia in 1974[35] in China and the data from U.S. CDC on the New York strain smallpox vaccination[36].

I. General reactions after vaccination

(I) Typical local reactions after vaccination

After the successful smallpox vaccination, there are generally papules, blisters, pustules, crusting

and scab falling off at the injection site, and also small lesions papules and blisters (called sub-pox) can be seen around the damaged skin, accompanied by localized itching and pain.

Typical local reactions after smallpox vaccination occur at the following time:

Time After accination	Typical Vaccination Reaction
**Day 3–4**	A papule appears at the vaccination site.
**Day 5–6**	The papule develops into a vesicle (blister) with a surrounding red halo, and the top of the vesicle becomes umbilicated (indented).
**Day 8–9**	The vesicle turns into a pustule, reaching its maximum size between days 8 and 10.
**Day 12+**	The pustule gradually dries and scabs begin to form.
**Day 17–21**	The scab falls off, leaving a permanent scar.

(II) Common systemic signs and symptoms after vaccination

Systemic symptoms after smallpox vaccination and their occurrence rates are: fever (30%), headache (40%), myalgia (20%), nausea (20%), fatigue (50%) and regional lymph node enlargement (50 %). These signs and symptoms usually go away within 2 weeks after vaccination by themselves or by only symptomatic treatment.

II. Abnormal reactions after vaccination and principles of treatment

(I) Concurrent bacterial infection at vaccination site

1. Clinical manifestations

The abnormal reactions are common in immunocompromised persons. They often occur within 2 to 3 weeks after vaccination, and include induration, swelling, increased skin temperature and pain at the vaccination site, as well as swollen local lymph nodes and systemic infection symptoms.

2.Treatment

Streptococcus or Staphylococcus aureus is the most important pathogen, but these reactions may also be caused by other bacterial infections. The culture should be done for tissues or blood, and the drug susceptibility testing should be conducted. The infection will be managed by use of

antibiotics.

(II) Transplanted pox

1. Clinical manifestations

It is seen in preschool and school-age children. This is caused by scratching the vaccination site using hands of those who were vaccinated, leading to virus heterotopic transplant, or by recently close contact with a person vaccinated with smallpox, leading to accidental infection. Transplanted pox can occur at near the vaccination or at other parts of body including eyes, mouth, lips, tongue, nose, back, anus and genitalia, etc.

The symptoms are more severe in persons with poxes transplanted into the eyes, and are not so serious in persons with poxes transplanted to other parts of body. Generally, the diseases of eye pox virus infection occur 5 to 12 days after vaccination. The symptoms are most common in eyelids: obviously swelling of eyelids, poxes of different sizes in eyelids or ruptured poxes to form shallow ulcers. Eye fissure is often sealed by a lot of secretions, and the eyes cannot be opened. It is often accompanied by significant conjunctival hyperemia, and can cause keratitis, iritis and the like. If not treated early, it may lead to sequela, such as loss of eyelashes, eyelid scarring, corneal scarring and etc., and can also affect vision, in severe cases, can cause blindness.

2.Prevention and treatment

The main measures for vaccinated persons or those in close contact with the vaccinated to avoid transplant poxes are: ① Avoid touching or scratching the vaccination site; ② Do not rub your eyes after contact with the vaccinated person, and often wash your hands with warm soapy water or 60% alcohol sanitizer; ③ Cover the vaccination site with breathable gauze or a bandage until the scab falls off, and the cover should be changed often to prevent leakage of exudate. The vaccinated person should wear long-sleeved clothes as a second layer for isolation; ④ Place the contaminated cover in a sealed plastic bag before disposal, and your clothing having contacted with the vaccination site should be cleaned using hot water and liquid detergent.

As for general transported pox, no special treatment is needed, but preventing the skin infections. Transplanted poxes at eyes should be treated as soon as possible, with principles as follows: ① If the vaccine accidentally splashes into the eyes, you must not rub with your hands, and should immediately flush several times with saline (or water), and then use idoxuridine eye drops, several times a day; ② Avoid scratching using your hands, and administer routine antibiotics to prevent bacterial infections. ③ Gamma-globulin eye-drops containing high titers of vaccinia virus

antibodies can be simultaneously used, but should not be used in patients with keratitis.

(III) Progressive pox (gangrene pox)

1. Clinical manifestations

It is seen in patients with defects in immune function, particularly in cellular immunity. It is characterized by progressive painless gangrene at injection site. Poxes have no scab formation two weeks after vaccination, continue to increase in size, and are not healed after a long term. Ulcers are expanding and deepening with significant central necrosis, forming a thick black eschar. Local inflammatory reactions are mild, and the similar pock lesions can also in other parts of the body. It is also accompanied by symptoms such as fever and systemic failure. If not treated, it will lead to a mortality rate.

2.Treatment

Early diagnosis and early treatment are very important. ① Keep dry at the lesion site, use antibiotics to control or prevent infections, and perform surgical treatment for burn wounds. ②Immunoglobulin containing high titers of vaccinia antibodies should be given in a timely manner. ③ Use of thiosemicarbazone and topical application of idoxuridine (IDU); ④ Systemic supportive therapy.

(IV) Systemic pox

1. Clinical manifestations

It is found in persons with low immunity or slow response, or those with poor resistance after an illness. At 6 to 14 days after vaccination, a number of poxes appear on skin all over the body, and they develop faster than the primary ones through four stages, i.e., papules, blisters, pustules and scabs. Poxes have a shape like umbilical concave with hard base, accompanied by systemic symptoms. It occurs only once in most of patients, and more than once in a small number of patients. After the scabs fall off, they form shallow scars, which can go away later.

2.Treatment

① Immunoglobulin containing high titers of vaccinia antibodies should be given in a timely manner; the treatment should be given by use of thiosemicarbazone and topical application of idoxuridine (IDU); ② Prevent secondary infection; ③ Symptomatic treatment.

(V) Eczema pox

1. Clinical manifestations

It is seen in patients with eczema, and occurs after vaccination or after close contact with vaccinated persons. It is characterized by multiple poxes with different sizes and often fused into a patch at the site of eczema or on normal skin, and it may have systemic symptoms such as high fever and listlessness. At this time point, the original eczema exacerbates, and it is often prone to secondary infection locally. The eczema pox is more severe in those with this condition caused by the contact with than in those who with this condition caused by vaccination.

2.Treatment

① Immunoglobulin containing high titers of vaccinia antibodies should be given in a timely manner; ② Keep local region dry and clean to prevent secondary infection, and give antimicrobial therapy in a timely manner in case of any infections; ③ Systemic supportive therapy, maintaining the balance of water and electrolyte; ④ Symptomatic treatment.

(VI) Allergic polymorphous rash

1. Clinical manifestations

It is caused by the allergies to the ingredients of vaccine. It often occurs in 2 to 5 days (also up to 2 weeks) after vaccination. The skin rashes have various shapes, including erythema, papules, maculopapulaes and urticaria, and one of them is the main in most cases. It can be accompanied by itching. Skin rashes often start from the face and then to all over the body, and can also involve oral mucosa. They can go away by themselves in 1 to 4 days. n severe cases, there may be blisters. The skin rashes are often accompanied by systemic symptoms such as fever. The blisters can be fused and have irregular shapes and different sizes. The surface is flat without depression, and its base presents the burn-like red erosion after rupture. A thin scab will be formed, and there will be no scar after the scab falls off.

Allergic polymorphous rash can also progress to Stevens-Johnson syndrome (SJS). It is mainly manifested as severe erythema multiforme, and can involve skin and mucous membranes.

2. Treatment

No special treatment is required for the mild condition. The severe cases can be treated by anti-allergy treatment such as chlorpheniramine, prednisone, etc., and the infection can be managed by use of antibiotics. Severe blister polymorphous rashes can be treated by supportive therapy using immunoglobulin containing high titers of vaccinia antibodies.

(VII) Purpura after vaccination

1. Clinical manifestations

It is the toxicity or allergic reaction of vaccinia. At 7 to 10 days after vaccination, the bleeding points with the size of needle tip are present on skin. They will gradually expand in different shapes and sizes, and can be fused into a patch. They are not extruding the skin, and are dark and purple in color. It can occur once or more. In severe cases, there may be hematuria, blood in the stool, and internal bleeding. The platelets are normal or decreased, and the blood clotting time was normal or slightly longer. It is often accompanied by systemic symptoms.

2. Treatment

(1) Anti-allergy treatment; (2) Use of hemostatic agents, and blood transfusion if necessary; (3) Trial use of immunoglobulin containing high titers of vaccinia antibodies.

(VIII) Encephalitis after vaccination

1. Clinical manifestations

It is seen in infants or those who are old or physically weak. It is manifested as symptoms of central nervous system damage such as high fever, headache, convulsions, vomiting, unconsciousness and increased intracranial pressure at 9 to 15 days after vaccination. Meningeal irritation sign and pathological reflex are positive. Cerebrospinal fluid pressure may increase, and the number of cells and proteins can be slightly increased. Generally, it will be gradually restored after about one week, and it can cause death in severe cases. Encephalitis caused by other infections or poisoning should be excluded.

2. Treatment

(1) Symptomatic and supportive treatments such as antispasmodic administration, cooling, and maintaining the balance of water and electrolyte; dehydrating agent will be given to those with significantly increased intracranial pressure and cerebral edema. (2) Drugs to promote the restoration of nerve cells and to improve the metabolism of nerve tissues should be given, such as VB12 and so on. (3) Use of immunoglobulin containing high titers of vaccinia antibodies is not recommended.

(IX) Encephalomyelitis after vaccination

1. Clinical manifestations

It is seen in infants or those who are old or physically weak. The latent period is 8 to 15 days. It is manifested as headache, vomiting and systemic discomfort, and it can develop into amnesia, delirium, disorientation, agitation, confusion, drowsiness, seizures and coma, accompanied by incontinence or urinary retention, obstinate constipation; Patients with this disease often have

mutism, dysphagia and unintentional acts, and the spinal cord can be involved.

2. Treatment

Intensive care, supportive therapy and symptomatic treatment.

Annex 7. Documents Stored for the Clinical Trial

I. Preparation stage of clinical trial

Document		Investigator	Sponsor
1	Investigator's Brochure	Retain	Retain
2	Signed Protocol and Amendments	Retain original	Retain
3	Case Report Form (Sample)	Retain	Retain
4	Informed Consent Form	Retain original	Retain
5	Financial Agreement	Retain	Retain
6	Signed Multi-Party Agreement (Investigator, Sponsor, CRO)	Retain	Retain
7	Ethics Committee Approval	Retain original	Retain
8	Ethics Committee Membership List	Retain original	Retain
9	Clinical Trial Application		Retain original
10	Preclinical Laboratory Data		Retain original
11	State Food and Drug Administration (SFDA) Approval	Retain	Retain original
12	Investigator's Curriculum Vitae and Related Documents	Retain	Retain original
13	Normal Range for Laboratory Tests Related to the Clinical Trial	Retain	Retain
14	Quality Control Certificates for Medical or Laboratory Procedures	Retain original	Retain
15	Labels for Investigational Product		Retain original
16	Shipping Records for Investigational Product and Related Materials	Retain	Retain
17	Certificate of Analysis for Investigational Product		Retain original
18	Unblinding Procedure for Blinded Trials		Retain original
19	Master Randomization List		Retain original
20	Monitoring Reports		Retain original

II. Conducting stage of clinical trial

Document		Investigator	Sponsor
21	Updated Investigator’s Brochure	Retain	Retain
22	Updates to Other Documents (Protocol, Case Report Form, Informed Consent Form, Written Notifications)	Retain	Retain
23	Curriculum Vitae of New Investigators	Retain	Retain original
24	Updates to Normal Ranges for Medical and Laboratory Tests/Procedures	Retain	Retain
25	Shipping Records for Investigational Product and Related Materials	Retain	Retain
26	Certificate of Analysis for New Batches of Investigational Product		Retain original
27	Monitor’s Visit Reports		Retain original
28	Signed Informed Consent Forms	Retain original	
29	Original Medical Records	Retain original	
30	Completed, Signed, and Dated Case Report Forms	Retain original	Retain copy
31	Investigator’s Serious Adverse Event Reports to the Sponsor	Retain original	Retain
32	Sponsor’s Unexpected Serious Adverse Drug Reaction Reports to Regulatory Authorities and Ethics Committees	Retain	Retain original
33	Interim or Annual Reports	Retain	Retain
34	Subject Identification Code List	Retain original	
35	Subject Screening and Enrollment Logs	Retain	Retain
36	Site Investigational Product Accountability Records	Retain	Retain
37	Investigator’s Signature Samples	Retain	Retain

III. After completion of the clinical trial

Document		Investigator	Sponsor
38	Certificate of Destruction of Investigational Product	Retain	Retain
39	Subject Identification Code List for Completed Trials	Retain	Retain
40	Audit Certificate		Retain original
41	Final Monitoring Report		Retain original
42	Treatment Allocation and Unblinding Documentation		Retain original
43	Trial Completion Report (to Ethics Committee and State Food and Drug Administration)		Retain original
44	Final Study Report	Retain	Retain original

Annex 8. Serious Adverse Events (SAEs) Report Form

Approval No. of Phase Ia clinical study of AIDS vaccine: Original number:

<table>
<tr><td colspan="2">Type of report</td><td colspan="2">□ Initial report
□ Follow up report
□ Summary report</td><td colspan="3">Reporting time:

(YYYY/MM/DD)</td></tr>
<tr><td colspan="2">Name of medical institution and specialties</td><td colspan="2">Peking Union Medical College Hospital, Clinical Infectious Diseases Specialty.</td><td colspan="3">Tel：63037608</td></tr>
<tr><td colspan="2">Name of sponsor(s)</td><td colspan="2">National Center for AIDS/STD Control and Prevention, Chinese Center for Disease Control and Prevention, Beijing Institute of Biological Products Co., Ltd.</td><td colspan="3">Tel：63037608</td></tr>
<tr><td colspan="2" rowspan="4">investigational vaccine</td><td rowspan="2">Phase Ia</td><td colspan="4">Name in Chinese: 重组天坛株痘苗病毒疫苗</td></tr>
<tr><td colspan="4">Name in English: Recombinant Vaccine Tiantan</td></tr>
<tr><td rowspan="2">Phase Ib,</td><td colspan="4">Name in Chinese: 艾滋病疫苗（核酸与重组天坛痘苗联合使用）</td></tr>
<tr><td colspan="4">Name in English: AIDS Vaccinia (Nucleic Acid in Combination with Recombinant Vaccinia Tiantan)</td></tr>
<tr><td colspan="2">Vaccine registration classification and dosage form</td><td colspan="5">Registration classification: prophylactic biological product Dosage form: Injection</td></tr>
<tr><td colspan="2">Clinical study classification</td><td colspan="2">☐ Phase Ia clinical observation
☐ Phase Ib clinical observation</td><td colspan="3">Clinical indications: Low- and high-risk populations of HIV infection</td></tr>
<tr><td rowspan="2">Basic information about subject</td><td>Initials:</td><td>Date of birth:</td><td>Gender:
□ Male □ Female</td><td>Height (cm):</td><td colspan="2">Weight (Kg):</td></tr>
<tr><td colspan="6">Concomitant diseases and treatment: □ Yes □ No
1. Disease: Treatment medication: Dosage and administration:
2. Disease: Treatment medication: Dosage and administration:
3. Disease: Treatment medication: Dosage and administration:</td></tr>
<tr><td colspan="2">SAE’s medical terminology (diagnosis)</td><td colspan="5"></td></tr>
<tr><td colspan="2">SAE situation</td><td colspan="5">□ Death Year Month Day
□ Leading to hospitalization</td></tr>
</table>

	□ Leading to prolonged hospitalization<br>□ Disability □ Dysfunction<br>□ Leading to congenital malformation<br>□ Life-threatening □ Other
Date of SAE onset:<br>(YYYY/MM/DD)	Date of informing investigator of SAE:<br>(YYYY/MM/DD)
Actions taken for investigational vaccine	□ Continuing with vaccination<br>□ Reducing the dosage<br>□ Suspending and then resuming<br>□ Stopping vaccination
SAE outcome	□ Symptom goes way (Sequelae □ Yes □ No)<br>□ Symptom persists
Relationship between SAE and investigational vaccine	□ Definitely related □ Possibly related<br>□ Possibly unrelated □ Definitely unrelated<br>□ Undeterminable
SAE reporting	China: □ Yes □ No □ Unknown;<br>Outside China: □ Yes □ No □ Unknown
Details of SAE occurrence and treatment:	

Name of reporting institution:

Position/title of reporter:

Signature of reporter:

References

1. http://www.unaids.org/en/HIV_data/2006GlobalReport
2. HIV & AIDS in China. http://www.avert.org/aidschina.htm
3. Ministry of Health of the People's Republic of China, "Joint Assessment Report on HIV/AIDS Prevention and Treatment in China." December 2003..
4. Ruprecht R M, Baba T W,Liska V. Attenuated HIV vaccine: caveats. Science, 1996;271:1790-2.
5. Ruprecht R M. Live attenuated AIDS viruses as vaccines: promise or peril? Immunol Rev, 1999;170:135-49.
6. Baba T W, Liska V, Khimani A H, et al. Live attenuated, multiply deleted simIan immunodeficiency virus causes AIDS in infant and adult macaques. Nat Med, 1999;5:194-203.
7. Keefer M. San Francisco C A Infectious Diseases Society of America Annual Meeting. 1995.
8. Rubinstein A, Goldstein H, Pettoello-Mantovani M, et al. Safety and immunogenicity of a V3 loop synthetic peptide conjugated to purified protein derivative in HIV-seronegative volunteers. Aids, 1995;9:243-51.
9. Naylor P H, Sztein M B, Wada S, et al. Preclinical and clinical studies on immunogenicity and safety of the HIV-1 p17-based synthetic peptide AIDS vaccine--HGP-30-KLH. Int J Immunopharmacol, 1991;13: S117-27.
10. Cohen J. Clinical research. A setback and an advance on the AIDS vaccine front. Science, 2003;300:28-9.
11. Donnelly J J, Ulmer J B, Shiver J W, et al.DNA vaccines. Annu Rev Immunol, 1997; 15:617-48.
12. W.Jaoko,G.Omosa K B. Safety and immunogenicty of DNA and MVA HIVA Vaccines in phase I HIV-1 vaccine trIals in Nairobi,Kenya. AIDS Vaccine04 Conference Lausanne Switzerland. WWW.AIDSVaccine04.org.
13. Pal R, Venzon D, Letvin N L, et al.ALVAC-SIV-gag-pol-env-based vaccination and macaque major histocompatibility complex class I (A*01) delay simIan immunodeficiency virus SIVmac-induced immunodeficiency. J Virol, 2002; 76:292-302.
14. AIDS Vaccine Evaluation Group 022 Protocol Team. Cellular and humoral immune responses to a canarypox vaccine containing human immunodeficiency virus type 1 Env, Gag, and Pro in combination with rgp120. J Infect Dis, 2001;183:563-70.
15. Bures R, Gaitan A, Zhu T, et al.Immunization with recombinant canarypox vectors expressing membrane-anchored glycoprotein 120 followed by glycoprotein 160 boosting fails to generate antibodies that neutralize R5 primary isolates of human immunodeficiency virus type 1. AIDS Res Hum Retroviruses, 2000;16:2019-35.
16. Check E, Army HIV vaccine to undergo clinical trIal as rival is halted. Nature, 2002;416:6.
17. Benson J, Chougnet C, Robert-Guroff M, et al. Recombinant vaccine-induced

protection against the highly pathogenic simIan immunodeficiency virus SIV(mac251): dependence on route of challenge exposure. J Virol, 1998; 72:4170-82.
18. Results from EV01 HIV Vaccine trIal, London and Lausanne, June 7th, 2004.http://www.eurovac.net/EV01Results.pdf.
19. IAVI report. http://www.Iavireport.org/specIals/OngoingTrIalsofPreventiveHIVVaccines.pdf
20. Shiver J W, Davies M E, Perry H C, et al.Humoral and cellular immunities elicited by HIV-1 vaccination. J Pharm Sci, 1996;85:1317-24.
21. Amara R R, Villinger F, Altman J D, et al. Control of a mucosal challenge and prevention of AIDS by a multiprotein DNA/MVA vaccine. Vaccine, 2002; 20:1949-55.
22. Robinson H L, DNA vaccines for immunodeficiency viruses. Aids, 1997; 11:S109-19.
23. Boyer J D, Ugen K E, Wang B, et al.Protection of chimpanzees from high-dose heterologous HIV-1 challenge by DNA vaccination. Nat Med, 1997;3:526-32.
24. Boyer J D, Wang B, Ugen K E, et al.In vivo protective anti-HIV immune responses in non-human primates through DNA immunization. J Med Primatol, 1996; 25:242-50.
25. Boyer J D, Cohen A D, Vogt S, et al.Vaccination of seronegative volunteers with a human immunodeficiency virus type 1 env/rev DNA vaccine induces antigen-specific proliferation and lymphocyte production of beta-chemokines. J Infect Dis, 2000; 181:476-83.
26. Amara R R, Villinger F, Staprans S I, et al.Different patterns of immune responses but similar control of a simIan-human immunodeficiency virus 89.6P mucosal challenge by modified vaccinIa virus Ankara (MVA) and DNA/MVA vaccines. J Virol, 2002;76:7625-31.
27. Chernos V I, ChelIapov N V, Antonova T P, et al.Verification of the safety, inoculability, reactogenicity and antigenic properties of a live recombinant smallpox-hepatitis B vaccine in an experiment in volunteers. Vopr Virusol, 1990;35:132-5.
28. Gu S Y, Huang T M, Ruan L, et al. First EBV vaccine trIal in humans using recombinant vaccinIa virus expressing the major membrane antigen. Dev Biol Stand, 1995;84:171-7.
29. Baldwin P J, van der Burg S H, Boswell C M, et al.VaccinIa-expressed human papillomavirus 16 and 18 e6 and e7 as a therapeutic vaccination for vulval and vaginal intraepithelIal neoplasIa. Clin Cancer Res, 2003;9:5205-13.
30. Polacino P, Stallard V, Klaniecki J E, et al.Limited breadth of the protective immunity elicited by simIan immunodeficiency virus SIVmne gp160 vaccines in a combination immunization regimen. J Virol, 1999;73:618-30.
31. Cooney E L, Collier A C, Greenberg P D, et al.Safety of and immunological response to a recombinant vaccinIa virus vaccine expressing HIV envelope glycoprotein. Lancet, 1991;337:567-72.
32. The achievement of global eradication of smallpox: final report of the global commission for the certification of smallpox eradication.Geneva 1979 Dec: 48

33. Frey SE, Couch RB, Tacket CO，et al. Clinical responses to undiluted and diluted smallpox vaccine. N Engl J Med. 2002; 346: 1265-74.
34. Talbot TR, Stapleton JT, Brady RC, et al. Vaccination success rate and reaction profile with diluted and undiluted smallpox vaccine: a randomized controlled trIal. JAMA. 2004; 292: 1205-12.
35. "Compilation of Materials from the 1974 Vaccine Experience Exchange Conference"
36. Smallpox Vaccination and Adverse Reactions Public Health Guidance for Clinicians. A Teaching Set Reviewing Key Points from the February 21, 2003 MMWR Recommendations and Reports.www.bt.cdc.gov/agent/smallpox
37. "Good Clinical Practice for Drugs" - State Food and Drug Administration (Order No. 3)
