# Supplementary material for: Safety and immunogenicity of an HIV vaccine trial with DNA prime and replicating vaccinia boost
Source: Signal Transduct Target Ther. 2025 Jul 2;10:208. doi: 10.1038/s41392-025-02259-y (PMC12217030; doi:10.1038/s41392-025-02259-y)
Supplement: Supplementary file 4 — Protocol 2 [file 41392_2025_2259_MOESM4_ESM.pdf]

AIDS Vaccine (Nucleic Acid in Combination with Recombinant Vaccinia Tiantan) Phase Ib Clinical Trial Protocol

Approval Number: 2008L04306

Sponsor:

National Center for AIDS/STD Control and Prevention, Chinese Center for Disease Control and Prevention

Project leader: Shao Yiming

Beijing Institute of Biological Products Co., Ltd.

Project leader: Xu Jing

Clinical Trial site:

Peking Union Medical College Hospital, Chinese Academy of Medical Sciences

Statement of Project Leader

I have read and agreed with this study protocol and will conduct this clinical study in accordance with the design and requirements in this protocol. The changes in this study protocol will not be made unless such changes have been agreed by both parties, and this study can be implemented only after it has been approved by the Ethics Committee.

National Center for AIDS/STD Control and Prevention, Chinese Center for Disease Control and Prevention	Peking Union Medical College Hospital, Chinese Academy of Medical Sciences
Professor Shao Yiming	Professor Taisheng Li
____________________	____________________
20 Y/M/D	20 Y/M/D

Table of Contents

Synopsis of the Clinical Trial Protocol 1
Table of Abbreviations 3
1 Introduction 4
1.1 Current Status of the AIDS Epidemic 4
1.2 Molecular Epidemiology of HIV 4
1.3 AIDS prevention and treatment 5
1.4 Overview of AIDS vaccine research 6
2 Study Product 7
2.1 DNA Vaccine 7
2.1.1 Research of AIDS DNA vaccine 7
2.1.2 HIV-1 DNA vaccine 7
2.2 Recombinant Vaccinia Virus Vaccine 8
2.2.1 Research of replication competent viral vector vaccines 8
2.2.2 Recombinant vaccinia virus vaccine 9
2.3 Summary of preclinical experiment 9
2.3.1 Safety evaluation in animal 9
2.3.2 Immunogenicity study in animal 10
2.4 Product Package, Doses and Method of Vaccination 10
3 Objectives and Rationale of the Study 11
4 Scientific Problems to be Solved by this Clinical Trial 12
5 Research Institutions and their Responsibilities 12
5.1 Responsibilities of Sponsor(s) 13
5.2 Responsibilities of Investigator(s) 14
5.3 Responsibilities of Data Management Center 15
6 Phase Ib Protocol 16
6.1 Objectives 16
6.2 Endpoint Events 16
6.2.1 Primary Endpoint Events 16
6.2.2 Secondary Endpoint Events 16
6.3 Recruitment of Study Subjects 17
6.4 Phase Ib Protocol 18
6.4.2 Immunization and Follow-Up Plan 20
6.4.3 Determination of Success of rTV Vaccination and re-Vaccination 27
6.5 Evaluation of Vaccine Safety 27
6.5.1 Evaluation of Local Adverse Events 28
6.5.2 Evaluation of Systemic Adverse Events 28
6.5.3 Evaluation of Ocular Adverse Events 28
6.5.4 Evaluation of Other Clinical Abnormalities 28
6.5.5Evaluation of Laboratory Test Results 29
6.5.6 Evaluation of the Relationship between Adverse Events and the Investigational Vaccine29
6.5.7 Report of Adverse Events 30
6.5.8 Treatment of Adverse Events 30
6.6 Evaluation of Vaccine Immunogenicity 31
6.6.1 Humoral Immune Response 31
6.6.2 Cellular Immune Response 32
6.6.3 The storage of HLA testing specimens. 32
6.7 Early Study Termination 32

6.7.1 Subjects Early Withdrawal from the Study or Terminate the Vaccination......................32
6.7.2 Early Termination of the Clinical Study 33
6.8 HIV-positive Problems and Treatment in the Study 33
6.8.1 Positive HIV Antibody Caused by Vaccination 33
6.8.2 HIV Infections during the Study..34
6.9 Data Management 34
6.9.1 Data Management System 34
6.9.2 Completion and Modification of CRF Forms...35
6.9.3 Lock of Database 35
6.9.4 Unblinding 35
6.10 Statistical Analysis 35
6.10.1 Study Indicators 35
6.10.2 Sample Size...36
6.10.3 Randomization 36
6.10.4 Blinding Design 36
6.10.5 Statistical Analysis Content and Methods...36
6.10.6 Hypothesis Testing and Significance 38
6.10.7 Treatment of Missing Values 38
6.10.8 Statistical Analysis Software..38
7 Management of Clinical Trial 38
7.1 Data Management 38
7.1.1 Data Management of the Clinical Study Site...38
7.1.2 Sponsor's Data Management 38
7.1.3 Ownership of Study Data...39
7.2 Confidentiality and Ethics...39
7.3 Quality Assurance and Quality Control of the Clinical Trial..39
7.3.1 Data and Safety Monitoring Board..39
7.3.2 Quality Assurance of Clinical Trial...39
7.3.3 Monitoring of the Clinical Trial...40
7.3.4 Audit and Inspection of the Clinical Trial...41
Annex 1. Informed Consent to Participate in a Phase Ib Clinical Study of AIDS vaccine (Nucleic Acid in Combination with Recombinant vaccina Tiantan) 42
Annex 2. Grading of Clinical and Laboratory Adverse Reactions 56
Annex 3. Subject Diary Card..60
Annex 4. Summary of Test Results for HIV-1 DNA Vaccine..63
Annex 5. Summary of Test Results of Recombinant Tiantan Vaccinia (rTV) AIDS Vaccine .65
Annex 6. Reactions after Smallpox Vaccination and Principles of Treatment 67
Annex 7. Documents Stored for the Clinical Trial 72
Annex 8. Serious Adverse Events (SAEs) Report Form...74

Synopsis of the Clinical Trial Protocol

The AIDS vaccine (Nucleic Acid in Combination with Recombinant Vaccinia Tiantan) has been jointly developed by the Center for Disease Control and Prevention of Sexually Transmitted Diseases and AIDS in China and the Beijing Institute of Biological Products. It consists of two components: the HIV-1 DNA vaccine and the recombinant vaccinia virus (rTV) AIDS vaccine. In accordance with NMPA requirements, the Phase I clinical trial protocol is designed to proceed in a stepwise manner, divided into two stages: Ia and Ib.

The Ia trial will observe the safety and immunogenicity of the rTV vaccine administered alone in 12 subjects. Following the acute reaction observation period after the Ia phase vaccination, safety data will be summarized and reported to the NMPA, and the Ib phase trial will commence upon receiving NMPA approval. A total of 48 subjects will be required for the trial, with an expected total study duration of approximately 58 weeks.

The Ia trial requires 12 subjects, including those who have and have not been vaccinated against smallpox, with a study duration of 26 weeks. The primary objective is to observe the safety and tolerability of administering either one or two doses of the rTV vaccine via bifurcated needle in healthy volunteers who have previously received or not received the smallpox vaccine, and to preliminarily assess its immunogenicity. Based on the occurrence of reactions to the rTV vaccine vector—the Tian Tan strain of vaccinia virus—the Ia phase trial will be divided into two stages: the acute reaction observation phase (weeks 0-8 of the trial) and the recovery phase observation (weeks 9-26 of the trial). The acute reaction observation phase will monitor typical post-vaccination reactions and any local or systemic adverse reactions occurring within 3-5 weeks thereafter. Upon completion of this phase, the trial will proceed to the recovery phase, where safety and immunogenicity will be observed, including the detection of immune responses specific to HIV antigens in the subjects, while continuing to monitor the various indicators from the first phase.

The Ib phase requires 36 subjects, including those who have and have not been vaccinated against smallpox, with a study duration of 38 weeks. The primary objective is to observe the safety and tolerability of a combined immunization regimen involving three doses of either 2 mg or 4 mg of the DNA vaccine and one dose of the rTV vaccine in healthy volunteers, and to preliminarily assess its immunogenicity. Subjects will be divided into a combined immunization group, a DNA-only immunization group, and a placebo group. The first two groups will receive three doses of either 2 mg or 4 mg of the DNA vaccine at four-week intervals. Four weeks after the last DNA vaccine administration, the combined immunization group will receive the rTV vaccine at a dose determined in the Ia phase trial, while the DNA-only group will receive a corresponding dose of the rTV vaccine placebo; the placebo group will receive the same volume of solvent for the DNA or rTV vaccine at the same time points. After each vaccination, subjects will be closely monitored for systemic and local reactions, and safety and HIV antigen-specific immune responses will be observed for at least 24 weeks following the rTV vaccine (or placebo) administration.

Following the approval of the Phase Ia clinical trial protocol by the Ethics Review Committee

of the National Clinical Trial Base at Peking Union Medical College Hospital and the Ethics Review Committee of the Center for Disease Control and Prevention of Sexually Transmitted Diseases and AIDS in China, the Phase Ia clinical trial was officially initiated at Peking Union Medical College Hospital and conducted in strict accordance with the Good Clinical Practice (GCP) guidelines, ensuring the protection of the subjects' interests while adhering to the research protocol.

All 12 subjects in the Phase Ia trial had completed immunization with the rTV vaccine, and all follow-up visits required by the trial protocol were completed. Statistical analysis of the results indicated that all subjects exhibited the expected local reactions following rTV vaccination, seroconversion for vaccinia virus antibodies, and no vaccine-related adverse events of grade 3 or higher occurred during the observation period. The trial results preliminarily demonstrated that the rTV vaccine could be successfully administered according to the methods described in the clinical trial protocol, that subjects exhibited good tolerability to the rTV vaccine, and that the rTV vaccine showed favorable safety in human subjects.

After the completion of the acute reaction observation period following the Ia phase vaccination, safety data were summarized and reported to the National Medical Products Administration (NMPA).

This protocol focuses on the research protocol for the Ib phase clinical trial.

Table of Abbreviations

Abbreviation	Full term
**AE**	Adverse Event
**ALVAC**	Avipoxvirus Canarypox
**ALT**	Alanine Transaminase
**AST**	Aspartate Aminotransferase
**AIDS**	Acquired Immunodeficiency Syndrome
**BLN**	Blood Urea Nitrogen
**CTL**	Cytotoxic T Lymphocyte
**CRF**	Case Report Form
**DMP**	Data Management Plan
**DRQ**	Data Query
**DSMC**	Data and Safety Monitoring Committees
**ELISA**	Enzyme-Linked Immunosorbent Assay
**ELISPOT**	Enzyme-Linked Immunospot Assay
**GCP**	Good Clinical Practice
**GMP**	Good Manufacturing Practice
**HIV**	Human Immunodeficiency Virus
**HIVAC-1e**	Recombinant Vaccinia Virus Vaccine Expressing HIV-1 Envelope Glycoprotein
**HLA**	Human Leukocyte Antigen
**HVTN**	HIV Vaccine Trials Network
**IC**	Informed Consent
**ICF**	Informed Consent Form
**IDU**	Idoxuridine
**IRB**	Institutional Review Board
**MVA**	Modified Vaccinia Virus Ankara
**NCAIDS**	National Center for AIDS/STD Control and Prevention，China CDC
**NIH**	National Institutes of Health
**NYVAC**	Copenhagen Strain of Vaccinia Virus
**PBMC**	Peripheral Blood Mononuclear Cell
**PCR**	Polymerase Chain Reaction
**PUMCH**	Peking Union Medical College Hospital
**QA**	Quality Assurance
**QC**	Quality Control
**RPR**	Rapid Plasma Reagin test
**rTV**	Recombinant Tiantan Vaccinia
**SAE**	Serious Adverse Event
**SAP**	Statatistical Analysis Plan
**SFDA**	State Food and Drug Administration
**SHIV**	Simian-human Immunodeficiency Virus
**SIV**	Simian Immunodeficiency Virus
**SJS**	Stevens-Johnson Syndrome
**SMP**	Study Monitoring Plan
**TPHA**	Treponema Pallidium Hemaggulination Test
**ULN**	Upper Limit of Normal

1 Introduction

1.1 Current Status of the AIDS Epidemic

Acquired Immunodeficiency Syndrome (AIDS) is an infectious disease caused by the Human Immunodeficiency Virus (HIV). Since the first case was identified in 1981, AIDS has spread at an alarming rate globally, becoming one of the most severe viral diseases threatening human life and health. Despite the annual increase in funding for AIDS prevention and treatment worldwide, and the ongoing promotion of antiretroviral therapy for HIV infection, the trend of AIDS prevalence continues to exceed expectations. By the end of 2007, the number of people living with HIV globally reached 33.2 million. In that year alone, approximately 2.5 million new HIV infections occurred, and around 2.1 million people died from AIDS-related causes.

In China, the epidemic of AIDS has progressed through phases of sporadic occurrence and localized outbreaks, transitioning into a rapid growth phase in the late 1990s. By the end of 2007, it was estimated that there were approximately 700,000 people living with HIV and AIDS in China, including about 85,000 AIDS patients. In 2007, there were approximately 50,000 new HIV infections in the country, and around 20,000 deaths due to AIDS.

The spread of AIDS has had a profound impact on social and economic development worldwide. In some developing countries, HIV infection has led to a reduction in life expectancy, a decrease in the labor force, and food shortages, causing economic and social development to regress by 20 years. As HIV-infected individuals are predominantly young adults, the loss of labor capacity directly results in decreased household income, while the costs associated with AIDS treatment further increase family expenditures. Statistics indicate that households of HIV-infected individuals in South Africa and Zambia have experienced a reduction in income of 66-80%, severely impacting the national economy.

Since the first reported cases of AIDS in China in 1985, the epidemic has undergone three phases: the introduction phase (1985-1988), the dissemination phase (1989-1994), and the growth phase (1995 to present). Currently, there is a clear upward trend in the prevalence of AIDS in China, with the epidemic among high-risk populations not being effectively controlled and beginning to spread to the general population. The peak of incidence and mortality in certain regions has already been reached. Furthermore, due to the widespread presence of risk factors for AIDS, there is a significant potential for further expansion of the epidemic in China. Therefore, the status of HIV prevalence poses a severe threat to the life and health of the Chinese population and is having a significant impact on social and economic development in China, as well as in Asia and globally.

1.2 Molecular Epidemiology of HIV

Through phylogenetic analysis of the HIV virus, HIV strains can be classified into discrete genetic subtypes. The HIV subtype B is primarily prevalent among male homosexual patients and intravenous drug users in Europe and North America, while in sub-Saharan Africa, India, and China, subtype B accounts for only a small proportion of infections. A significant number of infected individuals belong to subtype C or to B/C recombinant types, which are primarily

based on subtype C. Since the 1990s, the National Center for AIDS/STD Control and Prevention (NCAIDS) has conducted two large-scale national molecular epidemiological surveys of HIV, identifying a total of eight types of HIV-1 and HIV-2 strains, including subtypes A, B (Western B), B' (Thai B), C, D, E, F, and G across thirty provinces, municipalities, and autonomous regions, with over 80% being B' and C subtype HIV-1.

Further research has revealed that the B' and C subtypes of HIV-1 have undergone recombination in the context of their prevalence in China. This B'/C recombinant virus may have acquired a certain transmission advantage, leading to an accelerated trend in the spread of HIV. Investigations have shown that the proportion of infections caused by B'/C recombinant strains has been increasing annually among all infected individuals. In 1996, recombinant viruses were identified in only four provinces and municipalities in China (Xinjiang, Sichuan, Yunnan, and Shanghai). By 2000, they had spread to ten provinces, municipalities, and autonomous regions, including Xinjiang, Sichuan, Zhejiang, Gansu, Ningxia, Yunnan, Shanghai, Chongqing, Fujian, and Guangdong. By the end of 2002, this recombinant virus had been detected in most regions of the country, with the infection rate rising from 30.39% in 1996 to 50.20%.

Considering both the proportion of B'/C recombinant strains among infected individuals in China and the demonstrated transmission advantages of these strains, selecting the major antigen genes of the B'/C recombinant strain as immunogens for vaccines would be beneficial in rapidly curbing the trend of AIDS spread in the country.

1.3 AIDS prevention and treatment

Due to the high costs of antiretroviral therapy (ART), it is currently unrealistic to implement large-scale antiretroviral treatment in regions where HIV/AIDS is prevalent, particularly in developing countries. More importantly, there is currently no single drug that can completely eradicate HIV infection. The emergence of drug-resistant strains means that these medications are not effective for every patient, and the complexity of the treatment regimen prevents some patients from adhering to their medication schedules, leading to treatment failure. Therefore, there is an urgent need to explore safe and effective methods for preventing and controlling the spread of HIV/AIDS.

The prevention and control of AIDS primarily involve two aspects: first, the implementation of educational outreach and behavioral intervention measures; second, the development of vaccines for widespread administration among susceptible populations. Only by adopting a dual strategy that combines behavior interventions focused on education with biomedical interventions centered on vaccination can we ultimately control AIDS globally. Neglecting or abandoning either strategy will hinder efforts to control the epidemic. Historical evidence has shown that the most cost-effective and efficient method for controlling epidemic diseases is through vaccination, with successful examples including smallpox, poliomyelitis, measles, and hepatitis. Consequently, the development of a safe and effective AIDS vaccine has been a longstanding goal for researchers.

The Chinese government places great importance on the prevention and control of AIDS. In 1998, it issued the "Medium and Long-term Plan for the Prevention and Control of AIDS in

China," which established the goal of developing an AIDS vaccine targeting the prevalent strains in China and completing clinical trials by 2010.

1.4 Overview of AIDS vaccine research

The research on an AIDS vaccine can be divided into four phases: The first phase (1980s) marked the inception of AIDS vaccine research, characterized predominantly by single protein subunit vaccines aimed at inducing neutralizing antibodies, largely overlooking the role of cellular immunity. The second phase (1990s) saw an overemphasis on cellular immunity at the expense of neutralizing antibodies, with recombinant viral vector vaccines being the primary form. The third phase (2000-2005) focused on achieving a balance between humoral and cellular immune responses induced by vaccines, witnessing comprehensive development in DNA vaccines, live vector vaccines, and multivalent protein vaccines, and emphasizing combined immunization strategies, significantly accelerating the pace of vaccine clinical trials. The fourth phase (2005-present) has learned from the previous phases, with vaccine design now placing greater emphasis on antigen modification and the replicability of vectors to induce stronger humoral and cellular immune responses.

AIDS vaccine candidates include traditional vaccines (inactivated and live attenuated vaccines), synthetic peptide and protein subunit vaccines, DNA vaccines, and live vector vaccines. The basic research status of these vaccine forms is as follows: ① Due to safety concerns, researchers are cautious about the application of live attenuated vaccines in humans [4-6]. ② Inactivated vaccines cannot establish virus-specific protective immunity in monkeys. ③ Synthetic peptide vaccines are poor at stimulating the body to produce neutralizing antibodies and cellular immunity [7-9]. Subunit vaccines, such as highly purified recombinant monomeric HIV-1 envelope proteins, even when used with effective immunostimulants, do not elicit virus-specific cytotoxic T lymphocyte (CTL) responses, and the antibodies produced cannot neutralize HIV-1 primary isolates. Recently, VaxGen's phase III clinical trials of an AIDS subunit vaccine in the United States and Thailand concluded with disappointing results, showing no observed protective effect of the vaccine [10]. ④ DNA vaccines, capable of inducing effective cellular and humoral immune responses, offer good safety, low production and transportation costs, broad technical applicability, and short development cycles, showing promising application prospects [11]. ⑤ Live vector vaccines, primarily based on poxvirus vectors, have the advantages of actively infecting target tissues or cells, large vector capacity, intrinsic adjuvant effects, and the ability to induce long-term immune responses in most cases. These advantages align with the requirements for constructing an effective AIDS vaccine, making them a research hotspot. Poxvirus vector-based AIDS vaccine candidates have undergone several clinical trials, proving their safety, though their immunogenicity remains inconclusive [12-18]. ⑥ Boosting with live vector vaccines following DNA vaccine immunization offers unique advantages in inducing cellular immune responses and protective immunity, significantly enhancing the immune response induced by DNA vaccines while avoiding the impact of immune responses against the vector during multiple immunizations with live vector vaccines. Currently, the strategy of using DNA vaccines for initial immunization followed by live vector vaccine boosting, aiming to effectively induce HIV-specific CTL and neutralizing antibody responses, has

become the development strategy for a new generation of AIDS vaccines. Some candidates employing the DNA prime-poxvirus boost strategy have entered clinical trials [19].

The world's first AIDS vaccine clinical trial was conducted in the United States in 1987. By January 2006, 160 AIDS vaccine clinical trials had been conducted internationally, including 134 phase I, 13 phase I/II, 10 phase II, and 3 phase III trials, testing vaccine forms including DNA vaccines, protein/peptide vaccines, recombinant viral vector vaccines, and combinations thereof. Phase II-III clinical trial results indicated that not only did protein subunit vaccines, which could only induce a certain level of humoral immune response, fail to protect humans from HIV infection, but even non-replicating viral vector vaccines like ALVAC and MVA, which showed promise in animal experiments, could not induce sufficient humoral and cellular immune responses in humans, suggesting that viral vector vaccines lacking replicative capacity in humans may not be sufficient to protect against HIV infection.

2 Study Product

The vaccine proposed for this Phase Ib clinical trial is a DNA-Tian Tan vaccinia combined AIDS vaccine, which includes two components.

2.1 DNA Vaccine

2.1.1 Research of AIDS DNA vaccine

DNA vaccines have been shown to induce HIV- and simian immunodeficiency virus (SIV)-specific cytotoxic T lymphocytes (CTL), T-cell proliferative responses, and antibodies in mice and primates, and to protect chimpanzees from HIV infection [20-21]. In a phase I clinical trial of an HIV DNA vaccine conducted by the University of Oxford, 18 HIV-negative healthy volunteers were administered two intramuscular injections of the vaccine at two DNA doses (100 or 500 micrograms) three weeks apart. The results demonstrated that both doses were well tolerated in humans [22]. A preliminary trial of a DNA vaccine by the National Institutes of Health (NIH) in the United States indicated that a single injection of a 3 mg dose of DNA caused mild and rare adverse reactions [23]. In a phase I clinical trial organized by the U.S. AIDS Vaccine Evaluation Group, multiple injections of a 3 mg DNA dose at months 0, 1, 3, and 6 were well tolerated by all 52 volunteers and induced antigen-specific cell proliferation and chemokine production [24]. In a recent human trial organized by Merck, the safety of 1 mg and 5 mg DNA vaccine doses was explored, with two different adjuvants used in the 5 mg group, and immunizations administered at weeks 0, 4, 8, and 26. The company reported its findings at the HIV Vaccine Trials Network meeting in May 2003: the 5 mg DNA dose was well tolerated; and by week 12 post-immunization, the CTL response induced by the 5 mg dose was significantly stronger than that induced by the 1 mg dose [25].

2.1.2 HIV-1 DNA vaccine

The AIDS DNA vaccine in this study utilizes the pDRVISV1.0 vector system, which carries a kanamycin resistance gene. DNA vectors containing this type of resistance gene have been approved for use in humans by the U.S. Food and Drug Administration (FDA).

The DNA vaccine consists of two plasmids: pGP140 and pGPNef. The HIV genes expressed by these vectors are derived from the B'/C recombinant strain CN54, a predominant HIV strain in China. The pGP140 plasmid carries the gene encoding the transmembrane form of the envelope protein, gp140TM, while the pGPNef plasmid carries a fusion gene comprising gag, pol, and nef.

Based on the amino acid sequence of the CN54 strain, we optimized the env, gag, pol, and nef genes through codon humanization, RNA stability enhancement, and nuclear export signal modifications. These optimizations were implemented to improve the safety and expression efficiency of the genes. Key information about the DNA vaccine is summarized in Table 1.

Table 1. Main information of HIV-1 DNA vaccine

Plasmid name	pGP140	pGPNEF
**promoter**	CMV	CMV
**Full-length (bp)**	7118	9228
**HIV-1 genes**	Env	gag,pol,nef
**solvent**	20mM phosphate，0.9% NaCl, pH7.2	

We have produced 1,000 doses each of the pGP140 and pGPNef plasmids under Good Manufacturing Practice (GMP) conditions. All quality control tests for the DNA vaccine have been completed and passed, with the detailed test results provided in Appendix 4.

2.2 Recombinant Vaccinia Virus Vaccine

2.2.1 Research of replication competent viral vector vaccines

Currently, MVA (Modified Vaccinia Virus Ankara), ALVAC (Avipoxvirus Canarypox), and NYVAC (Copenhagen Strain of Vaccinia Virus) are the most extensively studied and widely used poxvirus vectors. Clinical trials have shown that these non-replicating vaccinia virus vector vaccines are very safe in humans, but their immunogenicity is relatively weak, and the immune responses they induce in humans are significantly weaker than those in monkeys [12, 26]. Although the reasons for the significant differences in immune effects between humans and animals are not yet clear, the weak immunogenicity of these vaccines may be related to the non-replicative nature of the vectors. After infecting host cells, these vector vaccines can only undergo one cycle of antigen expression, processing, and presentation, and cannot produce infectious viruses. Their stimulation of the immune system is limited and transient. In contrast, replicating vaccinia virus vectors can persistently and repeatedly stimulate the immune system over a longer period, thereby inducing strong immune responses in the body, which holds promise for long-term immune protection and memory.

Replicating recombinant poxviruses can protect animals against many veterinary diseases. Currently, several recombinant poxvirus veterinary vaccines have been approved for use. Replicating recombinant poxviruses as vectors for hepatitis B virus, Epstein-Barr virus, and therapeutic cancer vaccines have entered the human immunization observation phase, with some showing good immune effects [27-29]. In the SIV-macaque model, initial immunization with a recombinant vaccinia virus expressing the SIV env gene, followed by a boost with Env

protein, protected macaques from challenge with homologous strains [30]. The phase I clinical trial of a replicating recombinant vaccinia virus (HIVAC-1e) expressing HIV-1 env, developed by Cooney EL et al., showed that a booster immunization eight weeks after the initial HIVAC-1e vaccination could induce HIV-1-specific T lymphocyte proliferative responses and Env-specific antibodies lasting more than a year in test subjects who had not previously received the smallpox vaccine [31]. These results suggest that HIV vaccines based on replicating poxvirus vectors may have promising applications.

Since its isolation in the 1920s, the vaccinia virus Tian Tan strain has been used for smallpox prevention for several decades, immunizing over a billion Chinese people. Vaccinia virus is generally harmless to humans, causing only local, short-term symptoms at the vaccination site, with a very low incidence of more serious adverse reactions. The incidence of post-vaccination encephalitis with the Tian Tan strain is 0.5 per million, progressive vaccinia is 0 per million, and post-vaccination eczema and generalized vaccinia are 1 per million, making it safer than smallpox vaccine strains used in other countries. The high safety profile of the Tian Tan strain ensures its suitability as a vector for other vaccines.

2.2.2 Recombinant vaccinia virus vaccine

The vaccinia virus vector vaccine of this study is called recombinant vaccinia virus (rTV) AIDS vaccine, with the vector using Tiantann vaccinia strain 752-1, and is provided by Beijing Institute of Biological Products Co., Ltd. This Tiantan strain of vaccinia virus has been used in smallpox vaccine and extensively vaccinated in China.

The rTV vaccine expressed HIV genes are the main popular B’/C recombinant strain CN54 from China. The env (gp140TM), gag and pol genes act as the target genes and are inserted into TK region of vector. These HIV genes have been transformed to ensure the safety and immunogenicity of the vaccines.

We have manufactured 50,000 dosesof rTV vaccines in compliance with GMP conditions, and all items are tested qualified and the test results are shown in Annex 5. Main information of rTV vaccine is shown in Table 2.

Table 2. Main information of rTV vaccine

Vaccine	Recombinant vaccinia virus（rTV）
Vecter	Vaccinia virus Tiantan 752－1
HIV-1 genes	gag, pol, and env
Titer	1.6×10^7 PFU/ml
Solvent	60% glycerol, 1 % to 2 % starch, 5.0 % albumose

2.3 Summary of preclinical experiment

2.3.1 Safety evaluation in animal

The animal safety evaluation of the HIV-1 DNA vaccine and the rTV AIDS vaccine was

entrusted to the National Center for Safety Evaluation of Drugs at the National Institutes for Food and Drug Control, China. The test results demonstrated that both the DNA vaccine and the rTV vaccine exhibited good safety profiles in small animals.

2.3.2 Immunogenicity study in animal

Immunization experiments have shown that three doses of the DNA vaccine can induce strong antigen-specific humoral and cellular immune responses in mice; however, a fourth dose of the DNA vaccine did not significantly enhance the immune response compared to three doses. Initial immunization with three doses of the DNA vaccine followed by a single booster with the recombinant vaccinia virus (rTV) significantly enhanced antigen-specific humoral immune responses, although the cellular immune responses varied depending on the antigen, with some increasing and others decreasing. Overall, the strategy of priming with the DNA vaccine and boosting with the rTV demonstrated advantages over DNA vaccine immunization alone.

Mouse immunization experiments with rTV indicated that the intramuscular route tended to induce humoral immune responses, while the intradermal route appeared to be more effective for inducing cellular immune responses, consistent with the skin scarification route used for smallpox vaccination. The strength of the immune response induced by rTV was positively correlated with the immunization dose, with both cellular and humoral immune responses increasing as the dose was raised.

Rhesus macaque immunization experiments demonstrated that the DNA vaccine induced strong antigen-specific humoral and cellular immune responses, which were significantly enhanced following a booster immunization with rTV.

SHIV challenge experiments in rhesus macaques revealed that three doses of rTV immunization provided protection against SHIV infection in all monkeys (4/4). Following a DNA prime-rTV boost regimen, one out of four test monkeys was protected against SHIV infection, two out of four showed viral load at 14 days post-challenge, but the peak viral loads were 2 log units lower than the average of the control group, and one monkey died from acute intestinal infection one week before the challenge, precluding results. In contrast, three out of four control monkeys that received empty vector inoculations had detectable viral replication by day 14 post-challenge. These results suggest that vaccination with the rTV AIDS vaccine can protect animals from SHIV infection.

Detailed information on the vaccine's safety, immunogenicity, and monkey challenge experiments can be found in the investigator's brochure.

2.4 Product Package, Doses and Method of Vaccination

DNA vaccine is a colorless transparent liquid. It is supplied in 2ml vials with each vial of 2.0mg/1ml. The package specifies HIV-1 DNA vaccine (pGPNEF) or HIV-1 DNA vaccine (pGP140) and is stored at -70 ℃. Each of the two plasmids was collected by 1ml for

intramuscular injection at the bilateral deltoids of upper arms.

The rTV vaccine is a pale yellow or light brown turbid and viscous liquid. It is supplied in 0.4ml/vial. The package indicates recombinant vaccinia virus (rTV) AIDS vaccine and is stored at -20 ℃. The vaccination is performed with skin pricking method using a bifurcated needle. The vaccination method is as follows: insert the bifurcated needle into the vaccine vial, making it stained with enough vaccine liquid. Position your wrist against the vaccinated upper arm, perpendicularly direct the bifurcated needle to an area of 5mm in diameter at the deltoid of upper arm, and quickly prick 15 times. Prick with strength to the extent that bleeding points should appear at the pricking site in 15 to 30 seconds. If second smallpox will be vaccinated in the ipsilateral arm, another needle is needed to stain with vaccine for another vaccination at a site 2 cm away from the first vaccination site. When the site of vaccination becomes a little dry, it will be covered with gauze and dry breathable film.

Notes: Skin scarification is a conventional method of smallpox vaccination in China, and its operation process is as follows: first, drop 10μl vaccine (estimated dose of 2×10^5 PFU) at the vaccination site and then scarifies to make the vaccine go through the damaged skin into body, but the actual amount of vaccine into body is much lower than 10μl. According to the international trend in this field, we use the immunization method with a bifurcated needle recommended by WHO in this clinical study. This method is simple in immunization procedure and easy to grasp as compared with the traditional ones. Using a bifurcated needle, dip for a volume of about 2.5μl, it has the same vaccination effect as a skin scarification vaccination using four times of dose (10μl). It ensures the effect of immunization while reducing the dosage of vaccine and improving the absorption rate of vaccine as compared to the traditional methods [32]. Each bifurcated needle dips this vaccine containing approximately 0.4×10^5 PFU of rTV, and its immunization effect is equivalent to that of an estimated dose of 1.6×10^5 PFU in a traditional skin scarification method.

The concentration of this rTV vaccine is 1.6×10^7 PFU/ml, lower than the concentration of 1.0×10^8 PFU/ml for smallpox vaccine recommended by WHO. In a recent study, however, the smallpox vaccine was diluted by 5 folds and 10 folds to the concentrations of approximately 0.5×10^8PFU/ml and 1.0×10^7PFU/ml, and was vaccinated using a bifurcated needle in volunteers. The results showed that the success rate was 98% to 100% for three concentrations of vaccines, and that there were no significant differences in the size of timing of skin lesions after vaccination of the three vaccines [33,34]. These results suggest that the concentration of this vaccine may not affect its vaccination effect.

3 Objectives and Rationale of the Study

The objective of this clinical trial is to evaluate the safety and immunogenicity of an AIDS DNA vaccine and a recombinant vaccinia virus vector vaccine in healthy volunteers. For this study, we have selected the B'/C recombinant strain CN54 (CRF07), a prevalent HIV strain in China, as the pathogen model for designing the AIDS vaccine, providing a solid foundation for future vaccine evaluation and application. Existing research data indicate that DNA vaccines can induce protective immune responses specific to HIV and SIV in mice and primates, and there have been no reports of severe adverse reactions in humans following vaccination with such vaccines. Candidate AIDS vaccines based on vaccinia virus vectors have undergone several clinical trials, demonstrating good safety profiles and the ability to induce certain HIV-specific immune responses. The safety of the replicating vaccinia virus Tian Tan strain vector we employ has been validated through large-scale human application. Preclinical trial results have shown that the DNA vaccine and recombinant vaccinia virus vaccine we developed are well-tolerated in animals and can induce HIV-1 antigen-specific

cellular and humoral immune responses. Therefore, we anticipate that these two vaccines will have a good safety profile in humans and are expected to induce favorable immune responses.

4 Scientific Problems to be Solved by this Clinical Trial

This study represents the first clinical trial to evaluate the safety and immunogenicity of a DNA-Tian Tan vaccinia composite AIDS vaccine in humans. The scientific questions it aims to address are outlined in Table 3.

Table 3. Scientific problems to be solved by this phase Ib clinical trial

Problems	Primary safety endpoint events
Is a single intramuscular injection of 2 mg or 4 mg of the DNA vaccine safe?	The proportion of participants experiencing grade 3 or higher adverse events within 4 weeks after the first vaccination in each group.
Is it safe to administer three consecutive intramuscular injections of 2 mg or 4 mg of the DNA vaccine?	The proportion of participants experiencing grade 3 or higher adverse events within 4 weeks after each vaccination in each group.
Is the combined vaccination regimen of three doses of the DNA vaccine at different doses followed by one dose of the replicating recombinant vaccinia virus vaccine safe?	The proportion of participants experiencing grade 3 or higher adverse events from the first vaccination until 24 weeks after the last vaccination in each group.
	**Primary immunogenicity endpoint events**
Is there a qualitative or quantitative difference in the immune responses elicited by different doses of the DNA vaccine when boosted with the same dose of the recombinant vaccinia virus vaccine?	Antigen-specific ELISPOT and antibody responses will be measured at weeks 2, 4, 8, and 24 after the last vaccination.

5 Research Institutions and their Responsibilities

This trial is funded by the National High-Tech Research and Development Program of China (863 Program) and is supervised by the Clinical Pharmacology Trial Ethics Review Committee of Peking Union Medical College Hospital and the Ethics Review Committee of the National Center for AIDS/STD Control and Prevention (NCAIDS). The primary research institutions participating in the clinical trial and their responsibilities are listed in Table 4.

Table 4. The major research institutions of phase Ib clinical trial and their responsibilities

Research institution	Team leader	Responsibilities
National Institutes for Food and Drug Control (NIFDC)	Guowei Sang<br>Youchun Wang	Clinical trial guidance
Peking Union Medical College Hospital	Taisheng Li	Conduct the phase Ib clinical trial
NCAIDS	Yiming Shao	Provide AIDS vaccine (DNA), and the clinical trial organization
Beijing Institute of Biological Products Co., Ltd.	Jing Xu	Provide AIDS vaccine (rTV), and the clinical trial organization
Health Statistics Office, China CDC	Shuigao Jin	Clinical trial data management and statistical analysis

5.1 Responsibilities of Sponsor(s)

The National Center for AIDS/STD Control and Prevention (NCAIDS) and the Beijing Institute of Biological Products are the sponsors of the clinical trial, with responsibilities as follows:

（1） To recognize the qualifications and conditions of the investigators at the clinical trial sites to ensure the completion of the trial;

（2） To provide the clinical trial sites with an investigator's brochure, containing pharmaceutical, toxicological, and preclinical experimental data and information on the test vaccine;

（3） To initiate the clinical trial in accordance with this protocol after obtaining approval from the National Medical Products Administration and consent from the ethics committee;

（4） To collaborate with the investigators in designing the clinical trial protocol, specifying responsibilities in data processing, statistical analysis, results reporting, and publication methods, as well as the division of labor agreed upon with the investigators. To sign the mutually agreed trial protocol and contract;

（5） To assist investigators in recruiting subjects;

（6） To provide investigators with the test vaccine and instructions for use, ensuring the quality of the vaccine is qualified. To establish a management system and recording system for the registration, storage, and distribution of the test vaccine;

（7） To appoint monitors acceptable to the investigators to oversee the progress of the clinical trial;

（8） To be responsible for establishing a quality control and quality assurance system for the clinical trial. If necessary, to organize audits of the clinical trial to ensure quality;

（9） To collaborate with investigators in promptly studying any serious adverse events that occur, taking necessary measures to ensure the safety of subjects, and reporting to the drug regulatory authorities in a timely manner, while also informing other investigators involved in clinical trials of similar vaccines about the adverse events;

（10）To promptly notify investigators, the ethics committee, and the National Medical Products Administration of any early termination or suspension of the clinical trial, stating the reasons;

（11）To submit a summary report of the trial to the National Medical Products Administration, or a report on the termination of the trial and the reasons thereof;

（12）To be responsible for covering the costs of treatment or management for injuries or deaths caused by vaccination during the clinical trial, and to provide appropriate financial compensation to those who experience severe adverse reactions. To provide subjects with accidental injury insurance during the trial period;

（13）If an investigator does not comply with the approved protocol, Good Clinical Practice (GCP), or relevant regulations during the clinical trial, the sponsor should point this out to seek correction. If the situation is serious or persists, the sponsor should terminate the investigator's participation in the clinical trial and report to the National Medical Products Administration.

5.2 Responsibilities of Investigator(s)

Peking Union Medical College Hospital is the investigator for this clinical trial, with responsibilities as follows:

（1） The research personnel participating in the clinical trial should be familiar with Good Clinical Practice (GCP) and adhere to national laws, regulations, and ethical standards. They should have extensive experience in clinical trial methodology or receive academic guidance from experienced researchers within their institution, and possess the professional knowledge and experience required by the trial protocol;

（2） Be familiar with the materials and literature related to the clinical trial provided by the sponsor; understand and be familiar with the nature, effects, and safety of the investigational vaccine (including relevant data from preclinical studies), and also keep abreast of all new information related to the vaccine that arises during the course of the clinical trial;

（3） Have and be able to allocate the necessary personnel and equipment for the trial; possess all facilities to handle emergencies to ensure the safety of the subjects;

（4） Laboratory testing methods must be nationally certified, and laboratory results must be accurate and reliable;

（5） Must thoroughly read and understand the contents of the trial protocol, co-sign the clinical trial protocol with the sponsor, and strictly adhere to the protocol's stipulations; must promptly submit the clinical trial protocol and informed consent forms to the ethics committee for approval;

（6） Must obtain the consent of the hospital to ensure sufficient time is available to responsibly complete the clinical trial within the protocol's specified timeframe; must explain the trial's information, regulations, and responsibilities to all staff

involved in the clinical trial, ensuring an adequate number of informed and eligible subjects are enrolled according to the trial protocol;

（7） Take necessary measures to protect the safety of subjects and document these measures. Be responsible for making medical decisions related to the clinical trial, ensuring that subjects receive appropriate treatment for any adverse events that occur during the trial;

（8） In the event of a serious adverse event during the clinical trial, immediately provide appropriate treatment to the subject, report to the drug regulatory authorities, the sponsor, and the ethics committee, and sign and date the report;

（9） Ensure that subject data is accurately, completely, timely, and lawfully recorded in the case report forms;

（10） Accept monitoring and auditing by the sponsor's monitors or auditors, as well as inspections by the drug regulatory authorities, to ensure the quality of the clinical trial;

（11） Upon completion of the clinical trial, the investigator must write a summary report, sign and date it, and then send it to the sponsor;

（12） Must notify subjects, the sponsor, the ethics committee, and the National Medical Products Administration of any early termination or suspension of the clinical trial, stating the reasons.

5.3 Responsibilities of Data Management Center

The Health Statistics Office of the Chinese Center for Disease Control and Prevention (CDC) will serve as the data management center for this clinical trial, with responsibilities as follows:

（1） To be responsible for the statistical design of the clinical trial;

（2） To provide statistical requirements for the design of the Case Report Forms (CRFs) and participate in the revision of the CRFs;

（3） To complete the randomization process and blinding design according to the research requirements, and assist researchers in implementing the blinding;

（4） To complete the randomization process according to the research requirements;

（5） To be responsible for developing the Data Management Plan (DMP), organizing data entry, data checking, and management;

（6） To be responsible for developing the Statistical Analysis Plan (SAP), completing statistical analysis and reporting of results;

（7） To be responsible for developing the Study Monitoring Plan (SMP), completing the monitoring of the entire study;

（8） To be responsible for regularly submitting data and safety reports to the Data Safety Monitoring Board (DSMB).

6 Phase Ib Protocol

6.1 Objectives

Primary objectives：

1. To observe the safety and tolerability of 1-3 doses of 2 mg or 4 mg DNA vaccine in healthy volunteers.

2. To observe the safety and tolerability of a combined immunization regimen consisting of three doses of 2 mg or 4 mg DNA vaccine followed by one dose of rTV vaccine in healthy volunteers.

Secondary Trial Objectives:

To preliminarily evaluate the immunogenicity of the combined immunization regimen consisting of three doses of 2 mg or 4 mg DNA vaccine followed by one dose of rTV vaccine in healthy volunteers.

6.2 Endpoint Events

Study endpoint events are the endpoint expected to be observed in the clinical trial. Results of the clinical trial will be analyzed using the frequency of endpoint events.

6.2.1 Primary Endpoint Events

6.2.1.1 Primary Safety Endpoint Event

During the clinical trial, the safety indicators are graded according to Annex 2, and the following are the primary safety endpoint events:

（1） Grade 3 or higher local adverse reactions;

（2） Grade 3 or higher systemic adverse reactions;

（3） Any events of immunization regimen termination caused by vaccine problems;

（4） Any event leading to the discontinuation of the immunization schedule due to vaccine-related issues.

6.2.1.2 Primary immunogenicity endpoint event

The HIV-specific T-cell immune response was measured using the ELISPOT method at 2, 4, 8, 12, 16, 20, and 24 weeks following the last vaccination.

6.2.2 Secondary Endpoint Events

Information on secondary safety and immunogenicity endpoints will be collected according to the following criteria：

（1） All Grade 1 and 2 adverse events occurring within 4 weeks after each vaccination；

（2） The following responses will be measured at 2, 4, 8, 12, 16, 20, and 24 weeks after

the last vaccination：

-Titers of HIV total antibodies, Env, Gag binding antibodies, and HIV neutralizing antibodies;

-Proportions of HIV-specific cytokines such as IFN-γ and IL-2;

-Binding antibody titers against vaccinia virus following rTV immunization.

6.3 Recruitment of Study Subjects

Healthy volunteers will be recruited by the National Center for AIDS/STD Control and Prevention (NCAIDS) through outreach at universities and community organizations. Detailed information about the clinical trial will be provided to individuals interested in participating, and all questions regarding the trial will be addressed. Those who remain interested will be referred to Peking Union Medical College Hospital for screening. The hospital will organize the informed consent process for screening (see Appendix 1-II, Part 1). Individuals who agree to undergo screening will be assigned a screening registration number, and their personal information will be collected. They will then undergo physical examinations, specimen collection, and laboratory testing. Volunteers who meet the inclusion criteria will be asked to provide informed consent for participation in the clinical trial (see Appendix 1-II, Part 2), and will be assigned a randomization number and enrollment number in preparation for entering the clinical trial. The flowchart for volunteer recruitment and screening is as follows:

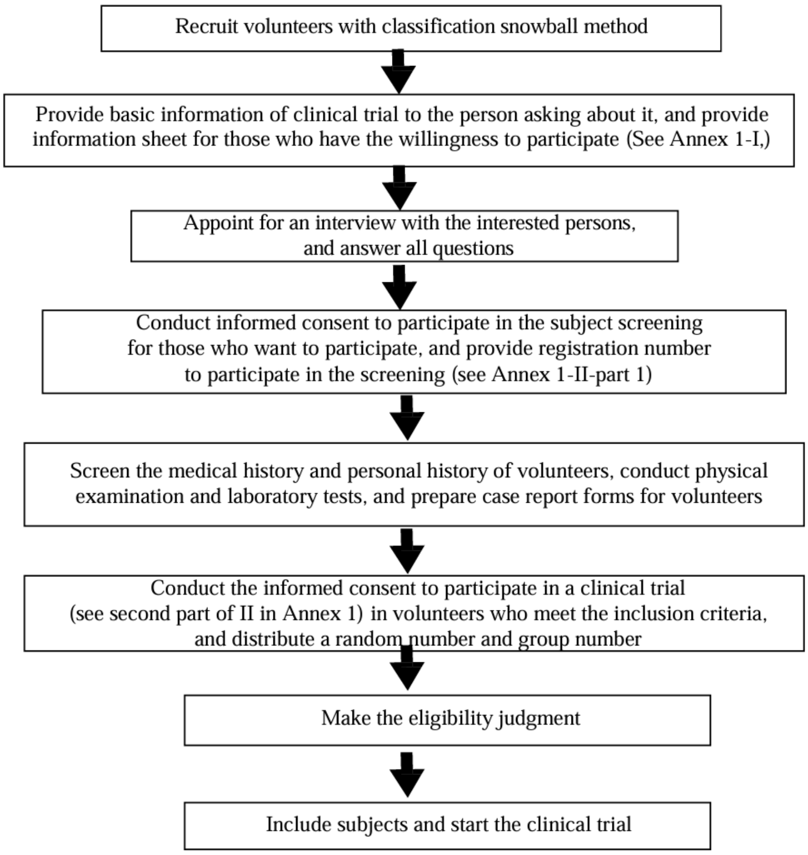

Figure 1 Subject recruitment and screening flowchart

6.4 Phase Ib Protocol

This Phase Ib clinical trial requires 36 participants and will span 38 weeks. The primary objective is to observe the safety and tolerability of a combined immunization regimen consisting of three doses of 2 mg or 4 mg DNA vaccine followed by one dose of rTV vaccine in healthy volunteers who have or have not previously received the smallpox vaccine. Additionally, the trial will preliminarily assess the immunogenicity of this regimen.

Participants who have received the smallpox vaccine must meet at least two of the following conditions:

1. Age ≥ 27 years;
2. Presence of a typical smallpox vaccination scar on the upper arm;
3. Positive vaccinia antibody test result.

Participants who have not received the smallpox vaccine must meet all of the following conditions:

1. Age < 27 years;
2. Absence of a typical smallpox vaccination scar on the upper arm;
3. Negative vaccinia antibody test result.

6.4.1 Inclusion and Exclusion Criteria of Study Subjects

**Inclusion criteria**

1. Gender: No restrictions.
2. Age: 18 to 55 years old.
3. Follow-up and Specimen Collection: Able to attend follow-up visits and agree to venous blood sampling and specimen storage within 9 months after the first vaccination.
4. Informed Consent: Able to understand and agree to the content of the informed consent form.
5. Low Risk of HIV Infection:
   - No history of gonorrhea or syphilis.
   - No high-risk sexual partners (e.g., intravenous drug users, HIV-positive partners) in the past year.
   - No unprotected sex with non-regular sexual partners in the past year.
6. HIV and Syphilis Testing: Willing to undergo HIV and syphilis blood testing.
7. Contraception: Willing to use effective contraceptive measures with their partner from 2 weeks before the first vaccination until 6 months after the last vaccination. Female volunteers must agree to undergo urine pregnancy testing before each vaccination and during follow-up

visits.

Exclusion criteria

1. Psychological State:

- Individuals deemed psychologically unfit to participate in the trial by a psychologist.

2. Pregnancy or Breastfeeding:

- Pregnant or breastfeeding women, or those planning to become pregnant during the trial.

- Close contacts (within 1 month after rTV vaccination) who are pregnant or breastfeeding.

3. Medical History or Conditions:

- Congenital or acquired immunodeficiency diseases (e.g., AIDS) or close contacts (within 1 month after rTV vaccination) with such conditions.

- Ongoing treatments that may affect immune responses, such as:

- Use of corticosteroids for more than 2 weeks.

- Use of immunosuppressants (e.g., alkylating agents, antimetabolites).

- Radiation therapy.

- Close contacts (within 1 month after rTV vaccination) undergoing such treatments.

- Immunosuppressive diseases (e.g., malignancies, organ or stem cell transplantation, agammaglobulinemia) or close contacts (within 1 month after rTV vaccination) with such conditions.

- History or current diagnosis of eczema or atopic dermatitis; current skin conditions causing skin damage (e.g., burns, chickenpox, impetigo, shingles, psoriasis) or close contacts (within 1 month after rTV vaccination) with such conditions.

- History or current diagnosis of hypertension, heart disease, diabetes, thyroid disorders, asthma, angioedema, asplenia, psychiatric disorders, psychological disorders, epilepsy, etc.

- Conditions requiring repeated injections or blood sampling.

- History of fainting or allergic reactions after vaccination.

- Current acute infectious or febrile illnesses.

4. Recent Medical Interventions or Behaviors:

- Received live attenuated vaccines within 2 months or other vaccines within 2 weeks.

- Received immunoglobulin or other blood products within 4 months.

- Participated in other medical product trials within 1 month.
- History of drug abuse, substance dependence, alcoholism, or heavy smoking.

5. Abnormal Laboratory Results:
   - HIV antibody positive or indeterminate, or HIV nucleic acid test positive.
   - Anti-DNA antibody or anti-nuclear antibody positive.
   - Hepatitis B surface antigen (HBsAg) or hepatitis C antibody positive, or rapid plasma reagin (RPR) test positive for syphilis.
   - Immunoglobulin G, A, or M levels outside the normal range.
   - Lymphocyte subset analysis showing abnormal levels of CD3+, CD4+, CD8+, CD8+DR+/CD8+, or CD8+CD38+/CD8+ cells.
   - Hemoglobin levels ≤110 g/L for females or ≤120 g/L for males.
   - White blood cell count ≤3.3×10^9/L or ≥12.0×10^9/L.
   - Total lymphocyte count ≤0.8×10^9/L.
   - Platelet count ≤100×10^9/L or ≥400×10^9/L.
   - Abnormal biochemical indicators: alanine aminotransferase (ALT), aspartate aminotransferase (AST), creatine kinase (CK), CK-MB isoenzyme, cardiac troponin I (cTnI), fasting blood glucose, total bilirubin (TBIL), blood urea nitrogen (BUN), creatinine (Cr), etc., outside the normal reference range.
   - Urine glucose ≥15 mmol/L or urine protein ≥0.3 g/L.
6. Other Exclusions:
   - Individuals unable to comply with the study protocol or provide informed consent due to medical, psychological, social, or occupational reasons.

These criteria ensure the safety of participants and the integrity of the trial results by excluding individuals at higher risk of adverse events or those whose conditions may interfere with the study outcomes.

6.4.2 Immunization and Follow-Up Plan

The Phase Ib clinical trial requires a total of 36 volunteers, including those who have and have not previously received the smallpox vaccine. These volunteers will be divided into three groups: a combined immunization group (24 individuals) receiving three doses of the DNA vaccine and one dose of the rTV vaccine, a DNA-only group (6 individuals) receiving three doses of the DNA vaccine alone, and a placebo group (6 individuals) receiving only a placebo. The combined immunization group is further divided into a low-dose and a high-dose subgroup, each consisting of 12 individuals. Recruitment will aim to include an equal number of individuals who have and have not received the smallpox vaccine in each

dose subgroup. Enrollment and vaccination will begin with the low-dose subgroup of the combined immunization group in the order of enrollment. The safety of the vaccine will be assessed within two weeks after each DNA vaccination in the low-dose subgroup. If no more than two vaccine-related adverse events of grade ≥3 (excluding fever above 39℃ for no more than 3 days) occur, the trial will continue; if three or more such adverse events occur, subsequent vaccinations will be halted. Participants in both dose subgroups of the combined immunization group and the DNA-only group will receive three doses of 2 mg (low-dose subgroup) or 4 mg (high-dose subgroup) of the DNA vaccine at 4-week intervals. Enrollment and vaccination for the DNA-only group will commence simultaneously with the high-dose subgroup of the combined immunization group. If no more than two vaccine-related adverse events of grade ≥3 occur after each 4 mg DNA vaccination in the high-dose subgroup and the DNA-only group, the trial will continue; if three or more such adverse events occur, subsequent vaccinations in that dose subgroup will be stopped. If no more than two vaccine-related adverse events of grade ≥3 occur after the third DNA vaccination in each group, the combined immunization group will receive the rTV vaccine at the dose determined by the Phase Ia trial four weeks after the last DNA vaccination, while the DNA-only group will receive a placebo of the corresponding dose of the rTV vaccine. The placebo group will begin enrollment and vaccination at the same time as the low-dose subgroup of the combined immunization group and will receive placebos of the same volume as the DNA or rTV vaccines at the same time points. After the last vaccine or placebo administration, all participants will undergo at least six consecutive months of clinical and laboratory observation. In the Phase Ib trial, the interval between vaccinations in each group will be ≥4 weeks; if the time required for adverse reactions to heal or resolve exceeds 4 weeks, the interval between vaccinations will be ≥ the actual time required for healing. The criteria for determining adverse events and their relationship to vaccination are detailed in Appendix 2 and Table 9.

Based on the data obtained from the Phase Ia clinical trial, which showed that the rTV vaccine was well tolerated at both 1 and 2 doses, with 2 doses being the highest safe dose determined in this phase, the Phase Ib clinical trial will use 2 doses as the vaccination dose for the rTV vaccine.

After immunization, participants will be observed for 24 hours, during which researchers will closely monitor local and systemic adverse reactions and complete raw data and CRF forms. Participants will then be given daily report cards and instructed on how to complete them. Participants must fill out the daily report cards every day for 2 weeks after DNA vaccination and for 4 weeks after rTV vaccination, and record any abnormalities that occur between follow-up visits on the daily report cards, which will be submitted to researchers at the next follow-up visit (Appendix 3). During the 2 weeks after DNA vaccination and the 4 weeks after rTV vaccination, participants must also complete a temperature chart for the respective periods. All adverse events occurring between follow-up visits will be recorded in the raw data forms, transcribed to the CRF forms, and entered into the database. Follow-up visits to the hospital will occur at weeks 1, 2, and 4 after DNA vaccination, and on days 3, weeks 1, 2, 4, 8, 12, 16, 20, and 24 after rTV vaccination. At each follow-up visit, researchers will record all participant-related data, including physical examinations and laboratory tests, in the raw

data forms and transcribe them to the CRF forms. During the trial, Phase Ib participants will need to visit Peking Union Medical College Hospital at least 20 times for trial-required visits, 14 of which will involve venous blood sampling. The immunization schedule, trial plan, and venous blood sampling plan for the Phase Ib trial are detailed in Tables 5, 6, and 7, respectively.

Table 5. Immunization Plan

Group	Number Of Subjects	Week								
		0	2	4	6	8	10	12	14	～38
DNA-L+rTV	12	DNA 2mg		DNA 2mg		DNA 2mg		rTV		Follow up
DNA-H+rTV	12		DNA 4mg		DNA 4mg		DNA 4mg		rTV	Follow up
DNA	6		DNA 4mg		DNA 4mg		DNA 4mg		rTV placebo	Follow up
placebo	6	DNA placebo		DNA placebo		DNA placebo		placebo		Follow up

Note:

(1) DNA vaccine is composed of 1/2 pGP140 and 1/2 pGPNef. Each of the two plasmids was collected by 1ml for intramuscular injection at the bilateral deltoids of upper arms. Control group: The placebo 1ml was collected for intramuscular injection at the bilateral deltoids of upper arms.

(2) The rTV vaccine was vaccinated for 2 poxes using a bifurcated needle on one side of the upper arm, and two bifurcated needles of placebo were vaccinated in the placebo group.

Table 6. Follow-up plan (the vaccination week is week 0)

Weeks	-2	0	1	2	4	5	6	8	9	10	12	12.5	13	14	16	20	24	28	32	36
Vaccination		DNA			DNA			DNA			rTV									
Personal information collection /HIV test counseling	X				X			X			X			X	X	X	X	X	X	X
Physical Examination	X	X	X	X	X	X	X	X	X	X	X	X	X	X	X	X	X	X	X	X
Anterior-posterior chest X-ray	X																			X
Electrocardiogram a	X				X			X			X		X	X	X	X	X	X	X	X
Routine blood test	X				X			X			X		X	X	X	X	X	X	X	X
Routine urine test	X				X			X			X		X	X	X	X	X	X	X	X
Liver and kidney functions and CK	X				X			X			X	Xb	X	X	X	X	X	X	X	X
CK-MB and cTnI a	X				X			X			X	X	X	X	X	X	—	—	—	X
HIV antibody	X				X			X			X			X	X	X	X	X	X	X
HIV nucleic acid c	X																			X
HBsAg and HCV antibody	X																			X
Syphilis test	X																			X
Urine pregnancy	X	X			X			X			X					X	X	X	X	X
DNA/ANA antibody	X				X			X			X									X
Immune function	X										X									X[e]
HLA typing		X																		
HIV antigen specific antibody (ELISA) d		X			X		X	X		X	X			X	X	X	X	X	X	X
HIV cellular immune response (ELISPOT)		X			X		X	X		X	X			X	X	X	X	X	X	X
HIV intracellular staining (ICS)		X			X		X	X		X	X			X	X	X	X	X	X	X
HIV neutralizing antibody d		X			X		X	X		X	X			X	X	X	X	X	X	X

**Vaccinia antibody**	X										X			X	X	X	X	X	X	X
**Vaccinia viremia testing**												X	X	X	X					

Notes:

a: If total creatine kinase (CK) levels are abnormal, additional tests for CK-MB, cardiac troponin, and electrocardiogram (ECG) should be performed at increased frequency, with repeated testing in the short term. The timing of these tests will be determined by the clinical physician.

b: Only CK testing is required.

c: If HIV antibody testing is positive after vaccination, HIV nucleic acid testing should also be performed.

d: Collect specimens at the corresponding time points for centralized antibody testing.

e: Only immunoglobulin G, A, and M levels will be tested during this follow-up visit.

Table 7. Blood collection form (the vaccination week is week 0) Unit: ml

week	-1	0	4	6	8	10	12	12.5	13	14	16	20	24	28	32	36
**Routine blood test**	2E		2E		2E		2E		2E	2E	2E	2E	2E	2E	2E	2E
**Liver and kidney functions + CK**	2		2		2		2	2	2	2	2	2	2	2	2	2
**CK-MB+cTnI**	3		3		3		3	3	3	3	3	3	—	—	—	3
**Syphilis test**	2															2
**IgG, IgA, IgM**	2						2									2
**DNA/ANA antibody**	2		2		2		2									2
**HbsAg + HCV antibody**	2															2
**HIV antibody**			2		2		2			2	2	2	2	2	2	
**T-cell phenotype.**	8E						4E									
**HIV nucleic acid test**			*		*		*			*	*	*	*	*	*	16E
**HIV cellular responses （ELISPOT）**		14E	14E	14E	14E	14E	14 E			14E	14E	14 E	14E	14E	14E	
**Vaccinia virus antibody**	2E									16E	16E	16E	16E	16E	16E	16E

**HIV antigen specific antibody (ELISA)**		**16E**	**16E**	**16E**	**16E**	**16E**	**16 E**									
**HIV cellular responses (ICS)**																
**HIV neutralizing antibody**																
**Cell cryopreservation**																
**HLA typing**																
**Vaccinia virus culture**								**7E**	**7E**	**7E**	**7E**					
**Total**	**25**	**30**	**41**	**30**	**41**	**30**	**47**	**12**	**14**	**46**	**46**	**39**	**36**	**36**	**36**	**47**

Notes:

1. Sample Collection:
   - Samples marked with "E" indicate EDTA anticoagulated blood; all others are non-anticoagulated blood.
2. Abnormal CK Levels:
   - If total creatine kinase (CK) levels are abnormal, additional tests for CK-MB, cardiac troponin, and electrocardiogram (ECG) should be performed at increased frequency, with repeated testing in the short term. The timing of these tests will be determined by the clinical physician.
3. Blinding and Unblinding:
   - For tests conducted at the NCAIDS laboratory, the specimens must be blinded by the Infectious Diseases Laboratory of Peking Union Medical College Hospital. After testing, the raw results must be returned to the Infectious Diseases Laboratory for unblinding by their personnel.
4. HIV Antibody and Nucleic Acid Testing:
   - If HIV antibody testing is positive after vaccination, HIV nucleic acid testing must also be performed.
5. HIV Antigen-Specific and Neutralizing Antibodies:
   - Specimens for HIV antigen-specific antibodies and neutralizing antibodies will be collected at the corresponding time points and tested in a centralized manner.

6.4.3 Determination of Success of rTV Vaccination and re-Vaccination

This trial uses the historical response to the Tian Tan strain vaccinia virus as a reference for the expected response following rTV vaccination.

Based on smallpox vaccination experience, first-time vaccine recipients typically exhibit a progression of skin reactions at the vaccination site, including papules, vesicles, pustules, scabs, and scab shedding, which generally takes about 3 weeks (see Appendix 6 for details). In individuals revaccinated with vaccinia virus, successful vaccination also follows this progression but with milder reactions and a shorter duration. For the rTV vaccine, the insertion of the HIV gene has significantly reduced the virulence of the Tian Tan strain vaccinia virus, and animal studies have shown that the toxicity of the rTV vaccine is 100–1000 times lower than that of the Tian Tan strain vaccinia virus. Therefore, the skin reaction induced by the rTV vaccine in humans may be milder than that of the Tian Tan strain smallpox vaccine. Additionally, due to technical issues, the vaccination site may only show mild needle trauma without the typical pustule formation, indicating vaccination failure.

For these reasons, rTV vaccination is considered unsuccessful if both of the following conditions are met:

1. The vaccination site shows no pustule formation as described above;
2. Serum vaccinia antibodies do not seroconvert (for individuals initially negative for vaccinia antibodies) or show no increase in antibody titer compared to pre-vaccination levels (for individuals initially positive for vaccinia antibodies) two weeks after vaccination.

In the Phase Ib trial, the data management center will unblind the clinical observers (while laboratory personnel remain blinded) on the third day after rTV vaccination to determine whether the rTV vaccination was successful. For individuals with unsuccessful rTV vaccination, a re-administration of the same dose will be performed on the contralateral upper arm four weeks after the first vaccination. Follow-up procedures after re-administration will follow the same protocol as after the initial rTV vaccination.

6.5 Evaluation of Vaccine Safety

After each immunization, participants will be observed for 24 hours, during which researchers will closely monitor local and systemic adverse reactions. On vaccination days or follow-up days, researchers will record participants' symptoms, physical signs, and laboratory test results in the raw data forms and transcribe them to the Case Report Forms (CRFs). Participants must complete daily report cards for 2 weeks after DNA vaccination and for 4 weeks after rTV vaccination, and record any abnormalities occurring between follow-up visits on the daily report cards, which will be submitted to researchers at the next follow-up visit (see Appendix 3). During the 2 weeks after DNA vaccination and the 4 weeks after rTV vaccination, participants must also complete a temperature chart for the respective periods. All adverse events occurring between follow-up visits will be recorded in the raw data forms, transcribed to the CRFs, and entered into the database. In cases of discrepancies between the

CRF and the daily report card, the CRF record will take precedence.

6.5.1 Evaluation of Local Adverse Events

Local adverse events will be evaluated according to the criteria outlined in Appendix 2, with the following explanations:

1. Pain at the vaccination site: This will be recorded by the participants themselves.
2. Skin erythema, swelling, papules, or induration at the injection site: These will be graded based on the maximum diameter of the reaction and its duration.
3. Blisters or ulcers: These will be graded based on their size, depth, healing time, nature of the blister (e.g., whether it is bloody), duration, and whether necrosis occurs.
4. Pustules: These will be graded based on their size, duration, and whether necrosis occurs.
5. Generalized rash: This will be graded based on the number of papules, presence of itching, exudation, and other characteristics.

6.5.2 Evaluation of Systemic Adverse Events

Systemic adverse events will be evaluated according to the criteria outlined in Appendix 2. The evaluation indicators include vital signs such as body temperature, heart rate/rhythm, blood pressure, and respiratory rate, as well as symptoms such as rash, headache, chills/shivering, fatigue/weakness, muscle/joint pain, nausea, vomiting, and diarrhea.

6.5.3 Evaluation of Ocular Adverse Events

During the skin reaction period following rTV vaccination, if participants touch the vaccination site or its covering with their hands and then come into contact with their own or others' eyes without proper disinfection, there is a risk of developing vaccinia-related eye diseases (e.g., blepharitis, conjunctivitis, keratitis). If participants or their close contacts experience eye symptoms, they must immediately seek medical attention at the clinical research site. The diagnosis will be recorded in the raw data forms and transcribed to the Case Report Forms (CRFs).

6.5.4 Evaluation of Other Clinical Abnormalities

For other adverse events, participants should promptly record the start and end dates of the event, as well as any treatment measures taken, on the daily report card. These events will be graded according to the criteria in Appendix 2. For clinical abnormalities not covered in Appendix 2, the severity will be assessed using the grading standards provided in Table 8.

Table 8. Criteria on intensity grading of adverse effects

**Grade 1**	Mild	A discomfort shorter than 48 hours, not requiring medical treatment
**Grade 2**	Moderate	Mild to moderate limitation of daily activities, not requiring or requiring only a little medical intervention
**Grade 3**	Severe	Significantly restrict daily activities, requiring the care of daily living, requiring medical treatment, and may need to be hospitalized

**Grade 4**	Life-threatening	Extremely restrict daily activities, significantly requiring the care of daily living, requiring medical treatment and hospitalization

6.5.5 Evaluation of Laboratory Test Results

The following tests will be conducted in the laboratory of Peking Union Medical College Hospital, which has passed national quality certification, following standard operating procedures. Abnormal results will be graded according to the criteria in Appendix 2.

1. Hematology tests: Hemoglobin, white blood cell count, and platelet count.

2. Blood biochemistry: ALT, AST, total bilirubin, blood urea nitrogen, creatinine, cardiac enzymes, etc.

3. Urinalysis: Urine protein, urine glucose, and blood cells.

Vaccinia viremia testing will be performed at the NCAIDS Virology and Immunology Laboratory using viral culture methods.

6.5.6 Evaluation of the Relationship between Adverse Events and the Investigational Vaccine

Adverse Events (AEs) refer to any unfavorable or unintended experiences that occur in participants during the clinical trial. Serious Adverse Events (SAEs) are those that result in any of the following outcomes:

1. Death;

2. Life-threatening conditions;

3. Hospitalization or prolongation of existing hospitalization (excluding hospitalization due to pre-existing conditions);

4. Persistent or significant disability or incapacity;

5. Congenital anomalies or birth defects (in cases where the participant is pregnant);

6. Other significant medical events (e.g., allergic reactions requiring emergency treatment, seizures not requiring hospitalization or medication, etc.).

Based on the criteria in Appendix 2, participants' systemic and local adverse events, as well as laboratory test abnormalities, will be graded. Social harm should also be classified as an adverse event.

According to their relationship to the investigational product, adverse events can be categorized into one of the following:

1. Definitely unrelated to vaccination;

2. Possibly unrelated to vaccination;

3. Possibly related to vaccination;

4. Definitely related to vaccination;

5. Unable to determine the relationship.

The latter three categories are considered adverse events potentially caused by vaccination and are classified as vaccination-related adverse events. Specific criteria for determining the relationship between adverse events and the investigational vaccine are provided in Table 9.

Table 9. Relationship between adverse events and the investigational vaccine

**Definitely Unrelated**	The symptoms differ from the expected post-vaccination reactions or are not temporally related to the vaccination. The symptoms may be caused by other diseases or treatments, and they resolve or disappear after the disease improves or the treatment is stopped.
**Possibly Unrelated**	The symptoms differ from the expected post-vaccination reactions or are not temporally related to the vaccination. The symptoms may be caused by other diseases or treatments.
**Possibly Related**	The symptoms are similar to the expected post-vaccination reactions and/or align with the expected timing of post-vaccination reactions. The symptoms resolve or improve after vaccination is stopped and cannot be explained by other causes.
**Definitely Related**	The symptoms and their timing are consistent with the expected post-vaccination reactions. The symptoms resolve or improve after vaccination is stopped and reappear upon re-vaccination.
**Unable to Determine**	The symptoms may be caused by factors outside the trial but are similar to post-vaccination reactions and/or their timing may be related to vaccination. It cannot be ruled out that the symptoms are adverse events caused by the vaccine.

The grading of adverse events will be determined after review by the head of the clinical trial unit. The relationship between adverse events and vaccination will be preliminarily assessed by the researchers at the clinical trial unit, but the final determination will be made through discussion between the sponsor and the clinical trial unit and confirmed in writing.

6.5.7 Report of Adverse Events

All adverse events must be recorded in the Case Report Forms (CRFs) and entered into the database. The records should include a general description of the symptoms and signs, the dates of onset and resolution, severity, relationship to the investigational vaccine or other medications, and measures taken to alleviate the symptoms.

If a participant experiences a serious adverse event (SAE) during the clinical trial, the investigator must immediately provide appropriate treatment to the participant. Simultaneously, the investigator must complete a Serious Adverse Event Report Form (see Appendix 8) and report the event to the Safety Supervision Department of the National Medical Products Administration (NMPA) within 24 hours, with copies sent to the Registration Department, the sponsor, the ethics committee, and the higher-level health administration authorities. The report must include the rationale for diagnosing the event as an SAE and be signed and dated by the investigator. Grade 3 adverse events must be reported to the clinical trial sponsor within 2 working days after the decision is made.

6.5.8 Treatment of Adverse Events

When a participant experiences an adverse event during the clinical trial, the investigator at the clinical trial site must closely monitor the participant and provide appropriate treatment,

following these principles:

1. Immediate treatment for serious adverse events: Provide emergency care for serious adverse events (SAEs) without delay.

2. Discontinuation of immunization under the following circumstances:

- If the participant exhibits the following symptoms within 2 hours after vaccination:

- Respiratory symptoms: including chest tightness, wheezing, palpitations, or difficulty breathing;

- Circulatory failure symptoms: including pallor or cyanosis, bradycardia, weak or absent pulse, hypotension, hypothermia, or cold extremities;

- Central nervous system symptoms: such as confusion, convulsions, or coma.

- If the participant experiences a grade 3 or higher systemic or local adverse event or laboratory abnormality related to vaccination.

3. Management of other adverse events: While conducting clinical observation and providing appropriate treatment, the timing of the participant's next vaccination should be postponed until the abnormal changes return to normal.

4. Handling adverse events after rTV vaccination: The assessment and management of adverse events following rTV vaccination should follow the principles for adverse reactions and management after smallpox vaccination (see Appendix 6).

In the event of a serious adverse event requiring emergency unblinding, the principal investigator of the clinical trial and the sponsor must jointly perform the unblinding.

6.6 Evaluation of Vaccine Immunogenicity

6.6.1 Humoral Immune Response

The following experiments will be conducted according to standard operating procedures:

1. Detection of HIV total antibodies and Env/Gag antibodies:

- Performed using the ELISA method.

- Confirmation of experimental results will be conducted by analyzing the following plate readings:

  - Specimens from all participants at the same time point.

  - Randomly selected specimens from the same participant at different time points.

2. Detection of HIV neutralizing antibodies:

- Performed using the TZM-bl method.

3. Detection of vaccinia virus binding antibodies:

- Performed using the ELISA method.

4. Detection of anti-DNA and anti-nuclear antibodies:

- Conducted at Peking Union Medical College Hospital.

6.6.2 Cellular Immune Response

ELISPOT Assay:

- Performed using freshly isolated PBMCs from blood samples.
- Stimulation is conducted using peptide pools of Env, Gag, Pol, and Nef.
- Experimental results are measured using an automated plate reader.

Intracellular Staining:

- Performed using freshly isolated PBMCs from blood samples.
- Two detection methods are employed:

Method 1:

- Positive control: PMA + Ionomycin.
- Stimulation is conducted using peptide pools of Env, Gag, Pol, and Nef.
- Experimental results are measured using flow cytometry.

Method 2:

- Positive control: SEB.
- Stimulation is conducted using peptide pools of Env and Gag.
- Experimental results are measured using flow cytometry.

6.6.3 The storage of HLA testing specimens.

To facilitate further evaluation of cellular immune responses, peripheral blood samples from participants should be stored at the time of enrollment for HLA typing.

6.7 Early Study Termination

6.7.1 Subjects Early Withdrawal from the Study or Terminate the Vaccination

Subjects can withdraw from the study for any reason at any time. The study of the subjects should be terminated early in case of the following circumstances during the study. The study staff will need record the time and reasons of withdrawal in the original data table and transcribe them to CRF.

1. Non-vaccination-related HIV antibody positivity:

- Stop subsequent vaccinations but continue follow-up.

2. Pregnancy:

- For pregnancies occurring after all vaccinations are completed, follow-up should continue with the participant's consent to ensure their safety.

3. Adverse events following vaccination:

- If the participant exhibits the following symptoms within 2 hours after vaccination:

- Respiratory symptoms: including chest tightness, palpitations, or difficulty breathing;

- Circulatory failure symptoms: including pallor or cyanosis, bradycardia, weak or absent pulse, hypotension, or cold extremities;

- Central nervous system symptoms: such as confusion, convulsions, or coma.

- If the participant experiences a grade 3 or higher systemic or local adverse event or laboratory abnormality related to vaccination.

4. Inability to participate in follow-up or specimen collection:

- If a participant's repeated inability to attend follow-ups or provide specimens significantly impacts data collection, the sponsor and clinical trial unit may decide to terminate their participation early.

For participants who withdraw due to adverse events, the reason and date of termination must be recorded on the adverse event report form and reported within 24 hours to the Safety Supervision Department of the National Medical Products Administration (NMPA), the sponsor, and the Institutional Review Board (IRB). Whenever possible, follow-up should continue until the expected end of the trial, but at least until the adverse event stabilizes or resolves.

6.7.2 Early Termination of the Clinical Study

In the low-dose combined immunization group, if three or more vaccine-related adverse events of grade ≥3 (excluding fever above 39℃ for no more than 3 days) occur after each vaccination, the sponsor and the clinical trial unit should terminate subsequent vaccinations for all groups. However, follow-up for the affected participants should continue at least until the adverse events are resolved.

6.8 HIV-positive Problems and Treatment in the Study

6.8.1 Positive HIV Antibody Caused by Vaccination

Participants may test positive for HIV antibodies after vaccination. Upon the participant's request, the National Center for AIDS/STD Control and Prevention (NCAIDS) will conduct additional nucleic acid testing to distinguish between HIV infection and HIV antibody positivity caused by vaccination. For participants who test positive on ELISA due to vaccination, NCAIDS will invite them annually for HIV antibody testing until the antibodies turn negative.

To mitigate potential social discrimination in areas such as education, employment, healthcare, and international travel, NCAIDS will issue a participation certificate card to participants, confirming their involvement in the AIDS vaccine clinical trial. The card will include the contact information of the relevant responsible person from the sponsor and bear the official seal of the National Center for AIDS/STD Control and Prevention, China CDC,

for use by participants as needed.

6.8.2 HIV Infections during the Study

If a participant tests positive for HIV antibodies or nucleic acids during the trial due to reasons unrelated to vaccination, the following steps will be taken:

1. Discontinue the participant's immunization:

- Stop all further vaccinations for the participant.

2. Refer the participant to a medical institution:

- Based on the participant's wish, recommend them to seek medical care at a healthcare facility.

3. Provide counseling by clinical trial unit researchers:

- Offer guidance on the following issues:

- Psychological and social aspects of HIV infection;

- Partner-related concerns;

- Measures to avoid infecting others.

4. Conduct immunological follow-up for participants who acquire HIV during the trial:

- The content and frequency of follow-up will be determined by the clinical researchers and the sponsor, based on the number of vaccinations received and the progression of the disease (including viral load and CD4 count).

6.9 Data Management

The data management for this study is the responsibility of the Health Statistics Office of the Chinese Center for Disease Control and Prevention (CDC).

6.9.1 Data Management System

The data management system used in this study is DataFax 3.7, a specialized system for managing clinical trial data.

The DataFax system is a computerized data management system that includes data transmission, database creation, data entry, data quality control, and data reporting. Its primary working principle involves transmitting scanned images of survey forms from the research site to the data center via fax or the internet. The DataFax system at the data center uses Intelligent Character Recognition (ICR) to convert these images into data, which is then entered into the study project database. All faxed images are stored on disks.

The DataFax system audits the data through pre-set quality control procedures and generates quality reports highlighting any issues. These reports are sent back to the clinical research site via fax or the internet. Clinical researchers can then immediately verify, correct, and resolve the identified issues, providing timely feedback to the data center.

Below is a schematic diagram of the DataFax system:

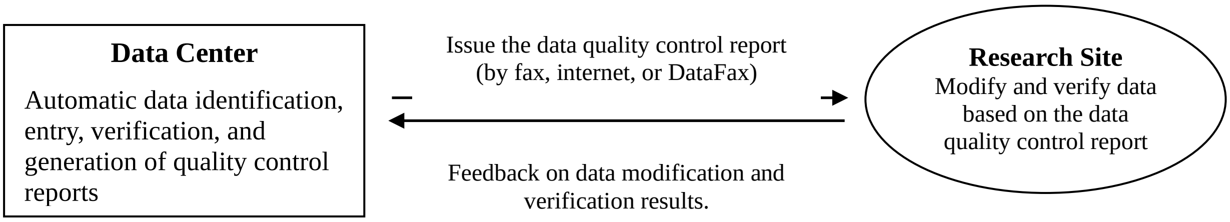

Figure 2. Schematic Diagram of the DataFax System.

6.9.2 Completion and Modification of CRF Forms

Clinical researchers complete the Case Report Forms (CRFs) for each enrolled participant. After review by data quality control personnel, the CRFs are transmitted to the DataFax system at the data center via fax or the internet.

If there are any questions or discrepancies in the CRFs, the data manager will generate a Data Resolution Query (DRQ) to inquire with the clinical researchers. The researchers should promptly address the queries, make necessary corrections on the original report forms, and then fax the updated forms back to the data center.

6.9.3 Lock of Database

After completing the collection of clinical and laboratory data and ensuring that the database meets the locking criteria, the clinical research unit, statistical analysts, and the sponsor will jointly lock the data. Once locked, the data files will no longer be modified. Any data errors identified after the data lock will be confirmed and corrected within the statistical analysis program.

6.9.4 Unblinding

In the Phase Ib trial, participants are enrolled and vaccinated with either the investigational vaccine or placebo under a blinded protocol. On days 3–4 after each rTV vaccination, once all relevant research data have been entered into the database, the blinding will be lifted jointly by the statistician, the principal investigator of the clinical trial, and the sponsor. In the event of a serious adverse event, the principal investigator of the clinical trial will perform an emergency unblinding using the emergency code-break envelope.

6.10 Statistical Analysis

6.10.1 Study Indicators

1) Safety Indicators

The incidence rates of various adverse events; abnormal change rates in laboratory test results such as vital signs, physical examinations, complete blood counts, blood biochemistry, and electrocardiograms.

2) Immunogenicity Indicators

The positive rates of vaccine-related humoral and cellular immune responses observed in

subjects.

6.10.2 Sample Size

In accordance with the clinical trial approval requirements of the China Food and Drug Administration, this Phase Ib study will enroll 36 healthy volunteers to receive the DNA-rTV vaccine.

6.10.3 Randomization

To avoid subjective selection bias by researchers and severe imbalance in sample allocation, a block randomization method will be employed in this trial. The random number table for randomization will be generated by statisticians from the Health Statistics Office of the Chinese Center for Disease Control and Prevention using SAS 9.1.2 software.

The 36 participants will be divided into two phases based on the order of enrollment, with 18 participants in each phase. In the first phase, participants will be randomized in a 2:1 ratio to either the low-dose combined immunization group or the placebo group. In the second phase, participants will be randomized in a 2:1 ratio to either the high-dose combined immunization group or the DNA-only group.

6.10.4 Blinding Design

Given that this trial includes a combined immunization group, a DNA-only group, and a placebo group, a blinded design will be implemented to prevent subjective bias among researchers during vaccination, clinical observation, and laboratory testing. However, since the reactions of participants before and after receiving the rTV vaccine and placebo are noticeably different, the blinding will no longer be effective for participants and clinical observers (but will remain effective for laboratory personnel) 3 days after the rTV vaccination.

In this Phase Ib trial, participants will be vaccinated with either the investigational vaccine or placebo under a blinded protocol. On days 3–4 after the last vaccination in each group, the blinding will be lifted jointly by the statistician, the principal investigator of the clinical trial, and the sponsor.

The blinding process will be carried out by personnel unrelated to the study, with assistance from the statistician, based on the random number table. This includes the preparation of blinded trial materials and emergency code-break envelopes. Each participant will be assigned a unique sequential number, with a corresponding blinded randomization code. After packaging the trial materials, the blinding codes will be stored in duplicate at both the clinical trial site and the sponsor's office.

6.10.5 Statistical Analysis Content and Methods

(1) Subject Enrollment and Trial Completion Status.

(2) Analysis of Baseline Data of Enrolled Subjects:

For the description of baseline characteristics, continuous variables will be calculated for mean, median, standard deviation, maximum, minimum, and interquartile range; categorical data will be calculated for frequency, percentage, cumulative frequency, and cumulative

percentage.

A comparative analysis of baseline data will be conducted for the combined immunization group, DNA-alone group, and placebo group in the Phase Ib study. For measurement indicators that meet parametric analysis conditions, methods such as analysis of variance (ANOVA), t-tests, and analysis of covariance (ANCOVA) will be primarily used. If parametric methods are not applicable, non-parametric statistical methods such as rank-sum tests will be employed; for categorical data, the chi-square (x^2) test will be used. Given the small sample size of this study, Fisher's exact test will be applied when the chi-square test is not suitable.

(3) Safety and Tolerability Evaluation:

Descriptive analysis will be used for safety evaluation, including vital signs (blood pressure, pulse, temperature, respiration), physical examinations, complete blood counts, blood biochemistry, and other laboratory tests at each follow-up period. Repeated measures ANOVA will be used for statistical comparison of measurement indicators across different follow-up periods. Electrocardiogram (ECG) results will list the status at each follow-up period and changes before and after the trial, along with a list of subjects with abnormal results.

Descriptive analysis will be performed for adverse events and adverse reactions (definitely related, possibly related, or indeterminable to vaccination) during the trial. For adverse events and reactions with higher incidence rates, the chi-square test will be used for statistical comparison; for those with very low incidence rates, statistical testing based on the Poisson distribution will be conducted.

Subjects included in the safety analysis must meet the following criteria:

- Meet all inclusion and exclusion criteria.
- Receive at least one dose of the vaccine.

(4) Immunogenicity Evaluation:

Immunogenicity evaluation will primarily be descriptive, reporting the positive rates of humoral and cellular immune responses.

For the Phase Ib study, differences in immunogenicity among different vaccination doses (combined immunization vs. DNA-alone), smallpox vaccination history, and study groups (vaccine vs. placebo) will be compared using logistic regression. The outcome variable (Y) will be the presence or absence of humoral/cellular immunity, with independent variables including vaccination dose (high vs. low), immunization strategy (combined vs. DNA-alone), smallpox vaccination history (vaccinated vs. unvaccinated), and study group (vaccine vs. placebo). For immune response intensity indicators such as antibody titers and Elispot counts, repeated measures ANOVA will be used. If the data do not meet the conditions for repeated measures ANOVA, appropriate data transformations will be applied.

(5) Exploratory Analysis:

Certain subject characteristics, such as gender and ethnicity, may influence study outcomes.

Therefore, identifying factors affecting vaccine safety and immunogenicity is an important aspect of exploratory analysis.

6.10.6 Hypothesis Testing and Significance

All statistical tests will be conducted at a 5% significance level. P-values will be provided for all hypothesis tests.

6.10.7 Treatment of Missing Values

In statistical analysis, missing values will not be imputed. Unless otherwise specified, all results are based on data with available analytical values.

6.10.8 Statistical Analysis Software

In this report, unless otherwise specified, all analysis results were generated using the licensed SAS 9.1.3 software. The software SITE number is: 47174001.

7 Management of Clinical Trial

7.1 Data Management

7.1.1 Data Management of the Clinical Study Site

The clinical trial unit is responsible for meticulously documenting the subjects' raw data sheets, preserving all test reports within these sheets, and ensuring they include the subject's identification number, specimen collection date, and the examiner's signature. In instances where test results are abnormal, it is imperative to note whether any measures have been taken, the date the report was reviewed, and the signature of the reviewing physician. Any alterations to the test results must be signed off by the research personnel and the date of the change recorded.

Researchers are required to accurately complete the Case Report Forms (CRFs) and transmit them to the clinical data management center. Upon receiving the data quality control report from the data center, researchers should promptly address any queries and make necessary amendments on the original report forms before faxing them back to the data center.

In compliance with the "Good Clinical Practice" guidelines issued by the National Medical Products Administration, the clinical trial unit must securely store all research materials from the trial phase, including original data sheets of subjects, laboratory result reports, original signed informed consent forms, CRFs, detailed treatment records, and other hospital original records, under dual-lock security by designated personnel until five years after the termination of the clinical trial. A list of materials that the clinical trial unit is required to retain can be found in Appendix 7.

7.1.2 Sponsor’s Data Management

In accordance with the "Good Clinical Practice for Drugs" regulations issued by the National Medical Products Administration, the NCAIDS Clinical Trial Management Core will designate specific personnel to securely store all documentation related to the application, approval, execution, and completion phases of clinical trials (detailed contents are listed in

Appendix 7) under dual-lock security. These documents will be retained until five years after the product in question is marketed or the product development is officially terminated.

7.1.3 Ownership of Study Data

All documentation pertaining to this clinical study is the property of the NCAIDS and the Beijing Institute of Biological Products. Except as required by the National Medical Products Administration, clinical researchers are prohibited from providing any form of this information to third parties without the written consent of the sponsor.

7.2 Confidentiality and Ethics

The clinical trial process will strictly adhere to the principles of medical confidentiality and will be conducted in accordance with the guidelines of Good Clinical Practice (GCP) and the provisions of the Helsinki Declaration (2000 edition). The clinical research protocol, information sheets provided to subjects, and informed consent forms must all be approved by the NCAIDS and the Clinical Pharmacology Ethics Review Committee of the Union Hospital. To protect the subjects, interviews with volunteers must be conducted in private settings, and information regarding the participation of volunteers in this trial must not be disclosed to individuals not involved in the research. All laboratory specimens, CRF forms, physical examination and test reports, and other records will be distinguished by numerical codes and stored in a secure location under the custody of designated personnel. No personal information of the subjects may be publicly disclosed without their consent.

7.3 Quality Assurance and Quality Control of the Clinical Trial

Both the sponsor and the clinical trial investigators are required to fulfill their respective responsibilities and strictly adhere to the clinical trial protocol, employing standard operating procedures to ensure the implementation of quality control and quality assurance systems throughout the trial. All observations and findings in the clinical trial must be verified, and quality control must be conducted at every stage of data processing to ensure the data's integrity, accuracy, authenticity, and reliability.

7.3.1 Data and Safety Monitoring Board

A Data Safety Monitoring Board (DSMB), composed of renowned domestic statisticians, clinical experts, vaccinologists, and ethicists independent of the study sponsor and clinical trial investigators, will be established. The DSMB is tasked with reviewing the study protocol, overseeing the execution of the clinical protocol, regularly reviewing data safety reports, and auditing the phases and final summary of the clinical trial. This is to safeguard the interests of the subjects and ensure the scientific integrity and completion of the clinical research. The DSMB will convene every two months to discuss research data reports submitted by the data center, guide the sponsor and clinical trial units in determining the relationship between serious adverse events and the study vaccine, and provide recommendations to the sponsor for modifying the clinical protocol or terminating the study. Following each meeting, the DSMB will submit written opinions and recommendations to the sponsor.

7.3.2 Quality Assurance of Clinical Trial

Before the commencement of the clinical trial, an operations manual or various standard

operating procedures (SOPs) must be established. These should encompass clinical trial management, subject recruitment, screening, enrollment, and follow-up, adverse event recording and reporting, specimen collection and management, biosafety and laboratory operations in clinical and immunology laboratories, transportation, usage, and storage of the trial vaccine, as well as data management and oversight. Participating units in the clinical trial must strictly adhere to the requirements of this clinical protocol, the operations manual, or the various SOPs in conducting the trial, and must appoint dedicated quality assurance personnel to oversee and control the quality of activities at each stage of the clinical trial, such as informed consent forms, CRF forms, adverse event report forms, and other trial records and reports. Quality assurance personnel are required to promptly complete trial logs, which should include the content of all trial activities for the day, the completeness and accuracy of documentation, responses to the data center's quality control reports, and whether there are any outstanding issues.

7.3.3 Monitoring of the Clinical Trial

The NCAIDS will be responsible for establishing a quality control and quality assurance system for the clinical trial, appointing monitors who have received appropriate training and are acceptable to the investigators to oversee the quality of the clinical trial, and reporting the progress of the trial and verifying data to the sponsor's responsible person. The content of clinical trial monitoring includes:

(1) Confirming before the trial that the trial unit has appropriate conditions, including staffing and training, well-equipped and functioning laboratory facilities, all necessary examination conditions related to the trial, an estimated sufficient number of subjects, and that the participating researchers are familiar with the requirements of the trial protocol;

(2) Monitoring the investigators' adherence to the research protocol during the trial, confirming that informed consent has been obtained from all subjects prior to the trial, understanding the enrollment rate and progress of the trial, and confirming that the enrolled subjects are eligible;

(3) Confirming that all trial data is recorded and reported correctly and completely, that all case report forms are filled out correctly and are consistent with the original data. All errors or omissions have been corrected, signed by the investigator, and dated. The number of vaccinations, dosage, follow-up and specimen collection after vaccination, adverse reactions after vaccination, omissions in follow-up and examination, loss to follow-up, and withdrawal from the group for each subject must be confirmed and recorded;

(4) Confirming that all adverse events are recorded, and that serious adverse reactions are reported and recorded within the stipulated time;

(5) Verifying the usage records of the trial vaccine, including quantity, shipment, receipt, storage, and the recovery and destruction of unused vaccine after application; verifying that the trial vaccine is used only for the subjects of this clinical trial and that the dosage and administration are in accordance with the requirements of the clinical trial protocol;

(6) Urging and assisting the clinical trial site personnel in reporting the clinical trial data and research results to the sponsor;

(7) The monitor must submit a written report to the sponsor after each monitoring visit, which should include the date and time of the monitoring, the name of the monitor, and the findings of the monitoring.

7.3.4 Audit and Inspection of the Clinical Trial

Depending on the progress of the trial, the sponsor or drug regulatory authorities will appoint auditors to conduct systematic inspections of clinical trial-related activities and documents to assess whether the trial is being conducted in accordance with the trial protocol, standard operating procedures, and relevant regulatory requirements, and whether the trial data is recorded in a timely, truthful, accurate, and complete manner. Audits should be performed by individuals not directly involved in the clinical trial. Both the sponsor and the clinical trial unit will also be subject to inspections by the drug regulatory authorities during the implementation of the trial, including inspections of the trial unit's and laboratory's relevant materials and documents, to evaluate the performance and execution of their respective tasks.

Annex 1. Informed Consent to Participate in a Phase Ib Clinical Study of AIDS vaccine (Nucleic Acid in Combination with Recombinant vaccina Tiantan)

I. Information about the Phase IIa Clinical Study of AIDS vaccine (Nucleic Acid in Combination with Recombinant vaccina Tiantan)

Study title: A phase IIa clinical study to evaluate the safety and immunogenicity of HIV DNA vaccine -combined recombinant vaccinia virus vaccine in healthy volunteers

Project leader：

Principal investigator：

Taisheng Li, Peking Union Medical College Hospital, Chinese Academy of Medical Sciences

Team leader of vaccine providing company：

Shao Yiming, National Center for AIDS/STD Control and Prevention, Chinese Center for Disease Control and Prevention;

Xu Jing, Beijing Institute of Biological Products Co., Ltd.

What is informed consent?

You are being invited to participate in a research study. Before deciding whether to participate, you first need to understand the details of this study and the possible benefits and risks from participation in this study. This process is called informed consent. This consent form will provide information about this study and its possible impact on your health and life for you to decide whether to participate in the study.

There may be some words or information that you do not understand in this document, and study staff familiar with this study will be explain to you what you do not know clearly. Please ask any questions that you may have to make sure you have fully understood what is involved in this study and why you are invited to participate in this study.

Why is the AIDS vaccine study being conducted, and what is the purpose of this study?

Since 1981 when the first case of HIV/AIDS was discovered, AIDS has been spreading at an alarming rate in the world, and becomes the world's most serious viral disease endangering human health. By the end of 2009, there are around 33.4 million people living with HIV. There were about 2.7 million persons newly infected with HIV only in 2008, and about 2 million people died of AIDS. AIDS epidemiology in China includes sporadic occurrence period, local epidemic period, and rapid increase period in late 1990s. By the end of 2009, it is estimated that there have been about 740,000 people infected with HIV and suffering from AIDS existing in China, including more than 100,000 AIDS patients. Only in 2009, there were about 48,000 new cases infected with HIV and about 26,000 people died

of AIDS in China.

Due to the severe global AIDS epidemic trends, there is an urgent need to develop a safe, effective and cheap AIDS vaccine. The purpose of AIDS vaccine study is to prevent HIV infection or delay the onset of AIDS/to reduce HIV viral load in patients.

What is a clinical trial?

A clinical trial is also known as a research study that means a systematic study in human (healthy volunteer or patient) on any new drug or new vaccine before being commercially available to find out if this new drug or new vaccine has side effects, how serious the side effects are, and if the expected prevention or treatment purposes can be achieved. Any vaccine must be tested in scientifically designed clinical trials to investigate its safety, immunogenicity and protection.

Need the clinical study of vaccine be approved by related department?

Clinical trials cannot be performed for all vaccines. The vaccine must be shown possibly safe and effective in human body by numerous pre-clinical studies. Vaccines must have received the quality test and safety evaluation in animals at designated units, and a certificate of conformity. All pre-clinical studies and evaluated data are technically reviewed by the State Food and Drug Administration (SFDA), and a clinical trial cannot be conducted unless the approval is obtained to conduct a clinical study.

In order to protect the safety and rights study participants (subjects), the clinical study of vaccine cannot be conducted until the protocol has been reviewed and approved by the ethics committee of the study site. Members of the Ethics Review Committee include medical professionals, non-medical professionals, legal experts and personnel from other units in order to ensure the fairness of the approval.

What is the current condition of the international study of AIDS vaccine?

The first clinical trial of AIDS vaccine was conducted in 1987 in USA. As of January 2006, international community has undertaken 205 clinical trials of AIDS vaccine, most of which are Phase I ones (including dozens of subjects), and a few of which are larger Phase II ones (including 200 ~ 300 subjects), and only three clinical trials are large-scale phase III ones (including several thousand subjects). The preliminary results of the current clinical trials show that AIDS vaccine tested is safe in human body. There are some achievements in studies of AIDS vaccines, but these vaccines are not effective in preventing HIV infection. Scientists are still working to develop new vaccines.

How about the product and vaccination method in this study?

The product for this Phase I clinical trial of an AIDS vaccine is a combination of a DNA vaccine and a recombinant Tian Tan vaccinia vaccine, which includes two vaccine components—the HIV-1 DNA vaccine and the recombinant vaccinia virus (rTV) AIDS vaccine. This vaccine received approval from the National Medical Products Administration in November 2006 to proceed with Phase I clinical trials. The trial is divided into two stages, Ia and Ib, requiring a total of 48 subjects, with an overall study duration of approximately 58 weeks. We are now inviting you to participate in the Ib phase of the trial.

The Ib phase requires 36 subjects, including those who have or have not been vaccinated

against smallpox, with a study duration of 38 weeks. During this phase, subjects will receive a vaccine once a month over a four-month period. The first three vaccinations will be with either a low or high dose of the HIV-1 DNA vaccine or a placebo, and the fourth vaccination will be with the rTV vaccine or a placebo. You may be randomly assigned to any dose group and could receive either four vaccines, three vaccines plus one placebo, or four placebos.

HIV-1DNA vaccine consists of two components called "plasmid", which use the same backbone (vector), but different HIV gene fragments. HIV gene fragments are artificially synthesized, and their sequence is the same as the most epidemic Chinese HIV strain, but has pathogenicity. A number of international clinical trials have been conducted for DNA vaccine of AIDS and other diseases, and all of them have shown that such vaccines are safe and well tolerated by human body. Recent reports show that higher doses of DNA vaccine are more likely to produce an immune response against the virus. Because it is impossible to infinitely concentrate DNA vaccine from the technical process, we need increase the dose of vaccine by increasing the injection volume. Therefore, you need receive an injection of 1ml vaccine at each side of the upper arm muscle (deltoid) every time.

The rTV AIDS vaccine uses Tiantan strain of vaccinia virus as a vector carrying HIV gene fragments. Tiantan strain vaccinia virus as a smallpox vaccine has been universally vaccinated in Chinese population, and it has played a good role in the prevention of smallpox, and successfully eradicates smallpox disease in China. This type of vaccine will be vaccinated by bifurcated needle skin prick method or intradermal injection. Among them, bifurcated needle skin prick method uses a bifurcated needle recommended by the World Health Organization to puncture several times on the lateral upper arm.

What are reactions after vaccination?

(1) Reactions after DNA vaccination

After DNA vaccination, you may develop the symptoms such as pain, mild swelling and low fever at the injection site, which can usually be tolerated, not requiring treatment.

(2) Reactions after rTV vaccination

The vector of this vaccine is Tiantan strain of vaccinia virus, therefore, the reactions caused by this virus used for smallpox vaccination in the past years can be used as a reference. However, the insertion of HIV genes has significantly reduced the virulence of Tiantan strain of vaccinia virus. The experiments of animals have shown that the toxicity of rTV vaccine in mice is 100-1000 times lower than that of Tiantan vaccinia virus, therefore, the adverse reactions caused by AIDS vaccine should be much lower than those caused by Tiantan strain smallpox vaccine.

Reactions after vaccination with Tiantan strain smallpox include general reactions and abnormal reactions. The general reactions are normal reactions to the successful vaccination of this vaccine. Pain may occur at the vaccination site, followed by papules, blisters, pustules and crusting, leaving permanent scars (if you have been vaccinated with smallpox vaccine, your upper arm will have such scars). These skin reactions will generally persist for 2 to 3 weeks. In addition, you may also develop fever, myalgia, chills, and symptoms such as rashes, nausea, headache, fatigue, axillary lymph node enlargement that are not at the vaccination

site. However, these symptoms will generally improve in several days, without the need to take medications.

In rare cases, there may some seriously abnormal reactions after vaccination of Tiantan strains of smallpox at a chance of approximately 5.6 per million. However, more than 80% of these abnormal reactions are seen in infants, but also in the elderly and physically weak persons as well as those with a history of eczema or immunocompromised persons. These abnormal reactions include transplanted pox, systemic pox systemic, progressive pox, eczema pox, allergic polymorphous rash and encephalopathy after vaccination, etc., and generally, they can be cured after treatment. In addition, if you have close contact with infants, pregnant women, or patients with immunocompromised diseases (such as AIDS, tumor, etc.), you will be likely to make them infected with vaccinia virus if not properly treated.

In the Phase Ia trial of this clinical study, a total of 18 doses of the rTV vaccine were administered to 12 subjects. During the follow-up period after vaccination, all subjects exhibited expected local reactions at the injection site, such as papules, vesicles, pustules, scabs, and scab detachment. There were no severe abnormal reactions as described above. Systemic discomfort related to the vaccination included mild swelling of the axillary lymph nodes (on the side of vaccination, which returned to normal within 1-4 weeks) and low-grade fever (one subject had a body temperature of 37.8℃, which improved within three days). Laboratory test changes included transient increases in blood creatine phosphokinase and mild decreases in peripheral white blood cell counts (observed in three and one subjects, respectively, during the trial phase, with no discomfort symptoms, no treatment required, and spontaneous return to normal). Since there are many causes for these laboratory changes, their relationship to the vaccination cannot be determined.

During volunteer screening, researchers will inquire about your personal circumstances and conduct physical examinations and laboratory tests to determine your suitability for the rTV vaccination. They will also guide you on appropriate personal protection measures to prevent the occurrence of severe adverse reactions and to avoid vaccinia virus infection in your contacts.

(3) Other post-vaccination reactions

Same as vaccination with other vaccines, in extremely occasional cases, due to the fasting, fatigue, nervousness and fear you may develop post-vaccination syncope (fainting); When you have an allergy to a component of the vaccine, you may develop symptoms such as chest tightness, shortness of breath, palpitations, difficulty breathing, pale or cyanosis, slow heart rate, blood pressure decrease, cold limbs, convulsions, coma and etc. These symptoms usually occur shortly after vaccination and will improve after symptomatic treatment.

(4) HIV testing issue after vaccination

The administration of the AIDS vaccine itself will not result in HIV infection. If you test positive for HIV, there are two possibilities: a positive result due to the vaccination or an HIV infection resulting from high-risk behavior. In this regard, the National Center for STD and AIDS Control and Prevention at the Chinese Center for Disease Control and Prevention will

conduct laboratory tests to distinguish between a true HIV infection and a post-vaccination reaction.

Positive HIV antibody caused by vaccination： Since the AIDS vaccine stimulates an immune response in the body, routine HIV tests may yield positive results after vaccination. If the HIV antibody positivity is due to the vaccination, researchers will invite you annually for HIV antibody testing until the antibodies turn negative. To mitigate potential social discrimination in education, employment, healthcare, and other areas, we will provide you with a proof card for participating in the AIDS vaccine clinical trial. This card will include the contact information of the responsible personnel at the research institution and will be stamped with the official seal of the National Center for STD and AIDS Control and Prevention at the Chinese Center for Disease Control and Prevention for your use when needed. It is recommended that you refrain from donating blood until your routine HIV test results turn negative.

HIV Infection: If you are found to be infected with HIV during the trial, researchers will discontinue your immunization and arrange a referral according to your wishes. Additionally, experts from Peking Union Medical College Hospital will provide you with counseling on psychological, social, and partner-related issues concerning HIV infection, as well as advice on how to avoid transmitting the risk to others.

Can I take part in the study?

This Phase Ib trial requires 36 volunteers, and both Beijing residents and long-term residents from other regions are eligible to participate. Researchers will determine your suitability for this study based on your personal circumstances and examination results. The conditions under which you may participate in the clinical trial are as follows:

**You are eligible to participate in the trial if you meet the following conditions:**	**You are NOT eligible to participate in the trial if you have any of the following conditions:**
1. Aged between 18 and 55 years, in good health;<br>2. Able to attend follow-up visits during the clinical trial period;<br>3. Capable of understanding and agreeing to the content of the informed consent form;<br>4. Currently in a low-risk state for HIV infection and willing to maintain this low-risk status during the study period:<br>• No history of intravenous drug use;<br>• No history of gonorrhea or syphilis in the past year;<br>• No current or past high-risk sexual partners (e.g., intravenous drug users, HIV-positive partners);	1. Pregnant or breastfeeding, planning to become pregnant during the trial, or having close contacts who are pregnant or breastfeeding within one month of vaccinia vaccination;<br>2. Having the following diseases or medical history:<br>• Congenital or acquired immunodeficiency diseases such as AIDS, or having close contacts with such patients within one month of vaccinia vaccination;<br>• Requiring treatments that affect immune responses, such as intravenous or intramuscular corticosteroids or oral prednisone for more than two weeks, or using immunosuppressants like alkylating agents, antimetabolites, etc., or receiving radiation therapy; or having close contacts who are receiving such treatments within one month of vaccinia

• No unprotected sexual intercourse with non-regular partners in the past year.<br>5. Agree to undergo approximately 14 venous blood draws and store blood samples;<br>6. Willing to undergo blood tests for HIV and syphilis;<br>7. From two weeks before the first vaccination until six months after the last vaccination, you and your sexual partner must use effective contraception (including combined oral contraceptives, injectable contraceptives, intrauterine devices, or consistent condom use); if you are a female volunteer, you must be willing to undergo urine pregnancy tests before vaccination and during follow-up visits.	vaccination;<br>• Suffering from immunosuppressive diseases such as malignant tumors, organ or stem cell transplantation, agammaglobulinemia, etc.; or having close contacts with such patients within one month of vaccinia vaccination;<br>• Past or current history of eczema or atopic dermatitis; currently suffering from skin-breaching diseases such as burns, scalds, chickenpox, impetigo, shingles, psoriasis, etc.; or having close contacts with such patients within one month of vaccinia vaccination;<br>• Past or current history of hypertension, heart disease, diabetes, thyroid disease, asthma, angioneurotic edema, asplenia, mental illness, psychological disorders, epilepsy, etc.;<br>• Suffering from diseases requiring repeated injections or blood draws;<br>• History of fainting or allergic reactions after vaccination;<br>• Currently suffering from acute infectious diseases or febrile illnesses.<br>3. Having the following situations:<br>• Received live attenuated vaccines within the last two months or other vaccines within the last two weeks;<br>• Received immunoglobulin or other blood products within the last four months;<br>• Participated in other medical product trials within the last month;<br>• Drug abuse, drug dependence, alcoholism, or heavy smoking.<br>4. Having the following laboratory test abnormalities:<br>• HIV antibody positive or suspicious;<br>• Hepatitis B surface antigen or hepatitis C antibody positive, or serological evidence of active syphilis;<br>• Anti-DNA antibody or antinuclear antibody (ANA) positive;<br>• Abnormal liver and kidney function, myocardial enzymes, blood glucose, etc.;

	• Abnormal humoral or cellular immune function;<br>• Other significant abnormalities in physical examination or laboratory tests that the doctor considers unsuitable for participation in the trial.<br>5. Inability to comply with the study protocol or obtain informed consent due to medical, psychological, social, occupational, or other reasons.

Will I have to take part in the study?

Please carefully read this information before deciding whether to participate in the trial. If you decide to participate, please sign this informed consent form. During the trial, you may withdraw from the study at any time without any reason. Your decision not to participate or to withdraw midway will not affect your treatment process at Union Hospital.

If I decide to participate in this trial, what do I need to do?

First, you will need to come to the hospital for a check-up. All your questions will be answered at that time. The researchers will determine if you are suitable to participate in this trial, and they will also ask you some personal questions related to HIV infection, and provide you with a free physical examination and blood tests, including an HIV test. If you meet the criteria for participants and agree to join the trial, the researchers will randomly assign you to a test group and begin the vaccination process.

After each vaccination, you will be kept under observation for 24 hours. The researchers will closely monitor your local and systemic reactions. Subsequently, the researchers will provide you with a daily report card and explain how to fill it out to help you record your reactions post-vaccination. You will fill out the daily report card every day for 2 weeks after the DNA vaccination and for 4 weeks after the rTV vaccination, and submit it to the researchers at each follow-up visit. Any discomfort experienced outside the hospital must be recorded on the daily report card, and if you experience any symptoms of grade three or higher as listed on the report card, you need to contact the medical staff as soon as possible. You will return to Union Hospital for follow-up visits at 1, 2, and 4 weeks after the DNA vaccination, and at 3 days, 1 week, 2 weeks, 4 weeks, 8 weeks, 12 weeks, 16 weeks, 20 weeks, and 24 weeks after the rTV vaccination. Therefore, you will need to visit Peking Union Medical College Hospital at least 20 times for trial-required visits, 14 of which will require venous blood draws. The maximum amount of blood drawn each time will not exceed 50ml. Your blood will be used directly for testing, but a small portion will be used for future analysis, for example, your blood will be used for leukocyte antigen typing to correctly assess the immune response caused by the vaccine.

To participate in this trial, you will also need to do the following:

• You need to continue to maintain a low-risk status for HIV infection.

• You and your spouse should avoid pregnancy from 2 weeks before the trial starts until 6 months after the last vaccination.

• You must not come into contact with the following individuals within one month after receiving the rTV vaccine: pregnant women, individuals with immunocompromising diseases (such as AIDS, leukemia and other cancers, organ transplants, lupus, etc.), individuals with eczema, and those with skin lesions, and infants under 1 year old.

• Due to individual differences, the expected skin reaction may not occur at the rTV vaccination site, and there may be no significant change in serum vaccinia virus antibodies before and after vaccination, indicating that the vaccination was not successful. In this case, the researchers will provide a supplementary vaccination on the opposite upper arm 4 weeks after the first vaccination, with the same dose as your first vaccination.

• After receiving the rTV vaccine and until the scab falls off, do not touch or scratch the vaccination site. If you accidentally touch the vaccination site, please wash your hands with soap immediately and avoid rubbing your eyes. Please cooperate with the medical staff to cover the vaccination site with breathable gauze or bandage until the scab falls off. You should also wear loose-fitting long-sleeved clothing before the scab falls off.

• Before the scab falls off, please try not to change the dressing by yourself. To prevent the dressing from falling off due to bathing, sweating, etc., during the follow-up visits from the rTV vaccination until the scab falls off, the researchers will provide you with dressings for replacement and waterproof dressings for use during bathing. At the same time, the researchers will provide you with a sealed plastic bag. If the dressing becomes loose during the follow-up period, you need to put the removed dressing into the sealed plastic bag and avoid contact with others. At the next follow-up visit, you need to bring the plastic bag to Union Hospital and hand it over to the medical staff for unified disposal.

• Your ability to attend follow-up visits on schedule is very important for the judgment of this trial's results. Please attend follow-up visits at the hospital on time as arranged by the researchers. If you are unable to attend the follow-up visit on time for any reason, please contact the researchers and inform them of the time when you can accept the follow-up visit.

• Since this vaccine is being tested for the first time, we will pay attention to your health status for a long time. We will contact you proactively every year. If your contact information changes, we hope you can keep in touch with us.

As compensation for your travel expenses and the inconvenience caused by frequent testing, we will pay you 9000 yuan at the last follow-up visit.

What should I do if I encounter problems during the trial?

If adverse reactions caused by vaccination occur during the clinical trial, the vaccine development unit will bear the cost of your diagnosis and treatment, and provide appropriate financial compensation to those who experience serious adverse reactions. For diseases that occur during the trial and are unrelated to vaccination or medical procedures, the researchers will assist in arranging your diagnosis and treatment, but you will need to cover the costs yourself. If you do not have accidental injury insurance, the vaccine development unit will

also provide this insurance for you during the trial period. If you experience psychological or social issues during the trial, you can consult with the researchers. If you have any concerns about our staff, please contact the project leader directly.

Is my participation confidential?

Yes. You will only need to provide your name and relevant personal information during the initial volunteer registration and when filling out the informed consent form at the beginning of the trial. This information will be securely stored by designated researchers and will not be disclosed to individuals not involved in the research. During the volunteer screening physical examination and laboratory tests, the researchers will assign you a participant number. All subsequent trial-related records will be distinguished using this numerical identifier. Interviews conducted by the researchers will take place in private settings. Without your consent, none of your personal information will be made public.

How will the trial results be handled?

The trial results will be summarized in the form of a scientific paper and submitted to medical journals or presented at scientific conferences, but your personal information will not be disclosed in any way.

**The potential benefits of participating in the trial for you:**<br>• You will undergo a full body check-up before entering the clinical trial and after the trial ends, providing an opportunity to assess your health status.<br>• You will receive information and preventive knowledge about sexually transmitted diseases, including AIDS.<br>• The information obtained from the trial will help scientists develop an effective AIDS vaccine, which could benefit hundreds of millions of people around the world who are under the threat of AIDS.
**The potential inconveniences and risks of participating in the trial for you:**<br>• You will need to visit the hospital multiple times for follow-ups, which may cause inconvenience to your work and life.<br>• Vaccination may cause you physical discomfort. Although the incidence is very low, there is still a risk of more serious adverse reactions after vaccination.<br>• For 1 month after receiving the vaccinia virus vaccine, your activities and contact with people will be somewhat restricted.<br>• If your blood tests positive for HIV antibodies due to vaccination, you may face some social discrimination.<br>• You might mistakenly believe that after vaccination, you are resistant to HIV infection and therefore can engage in high-risk behaviors. This is a misconception, as it cannot be guaranteed that the vaccine has a protective effect before the clinical trial is completed.

• After participating in this trial, you will not be able to participate in any clinical trials for AIDS vaccines.

If you have any questions or you want to get more information, please contact:

National Center for AIDS/STD Control and Prevention, Chinese Center for Disease Control and Prevention

Contactor ____Yiming Shao____________________

Tel ____010－58900981______________

Peking Union Medical College Hospital, Department of Infectious Diseases

Contactor ____Taishneg Li____________________

Tel ____010－65295086________________

Peking Union Medical College Hospital, Clinical Pharmacology Trial Ethics Committee

Contactor ____Chi Zhang_______________________

Tel ____010－88068354_______________

Please keep a copy of this information sheet. If you volunteer to participate in this research study, please sign on this information sheet, and read and sign the informed consent form to participate in a screening.

Volunteer name: ___________Signature:__________Date: _______________

Name of witness: __________Signature:__________Date: _______________

Name of investigator:______Signature:__________Date: _______________

II. Informed Consent Form

Part I: The information and consent form for subjects to agree to participate in a screening of phase Ib clinical study of AIDS vaccine

Screening Number: Date of Birth: Year Month Day

The subject must personally complete all the following questions.
Please select your answer.

Have you received the information sheet for the Phase I clinical trial of the AIDS vaccine?
Yes No

Do you understand the purpose of the AIDS vaccine clinical trial?
Yes No

Do you understand the information about the product and the trial plan of this vaccine clinical trial?
Yes No

Have you had the opportunity to ask questions or participate in discussions regarding this trial?
Yes No

Have all the questions you raised been satisfactorily answered?
Yes No

Are you fully aware that you need to undergo HIV testing before and during the trial in order to participate in this study?
Yes No

Do you understand that you and your spouse need to use contraception from 2 weeks before the first immunization until 6 months after the last immunization in this trial?
Yes No

Please fill in the name of the researcher who discussed the trial details with you:

Do you understand that you can withdraw from this trial at any time without any reason, and it will not affect your future medical commitments?
Yes No

Do you agree to participate in the screening as a candidate for this trial?
Yes No

Volunteer Name:_________ Signature:_____________ Date:____________

Witness Name:_________ Signature:_____________ Date:____________

Researcher Name:_________ Signature:_____________ Date:____________

Part II: The information and consent form to agree to participate in phase Ib clinical study of AIDS vaccine

Screening Number: Date of Birth: Year Month Day

The subject must personally complete all the following questions.

Please select your answer.

Have you received sufficient information about this study?

Yes No

Do you understand that it is uncertain whether the vaccine being tested can prevent HIV infection?

Yes No

Do you agree to retain your specimens for future immunological experiments of HIV vaccines?

Yes No

Are you aware of the serious adverse reactions that may occur after vaccination?

Yes No

Do you anticipate having close contacts within 1 month after receiving the recombinant vaccinia virus vaccine who are immunocompromised patients (such as those with AIDS, leukemia, cancer, organ transplants, lupus, immunodeficiency diseases), or patients undergoing treatments that may reduce immune function (such as hormones, chemotherapy, radiotherapy, immunosuppressants)?

Yes No

Do you anticipate having close contacts within 1 month after receiving the recombinant vaccinia virus vaccine who are infants under 1 year old, pregnant women, or patients with eczema or other skin diseases?

Yes No

Do you agree to participate in this trial?

Yes No

Are you willing to accept long-term follow-up and regular contact?

Yes No

Do you agree to be visited by medical staff every other day for 2-3 weeks after receiving the rTV vaccine?

Yes No

Are you aware that you need to sign two copies of the informed consent form, one for you to keep and the other to be kept by the research unit?

Yes No

Volunteer Name:_________ Signature:_____________ Date:____________

Witness Name:_________ Signature:_____________ Date:____________

Researcher Name:_________Signature:_____________ Date:____________

Annex 2. Grading of Clinical and Laboratory Adverse Reactions

Parameter	Mild (Grade 1)	Moderate (Grade 2)	Severe (Grade 3)	Potentially Life-Threatening (Grade 4)
**Laboratory Tests**				
**Hematology**				
Hemoglobin (g/L)	95–105	80–94	65–79	<65
Leukocytosis (10^9/L)	13.1–15	15.1–20	20.1–30	>30
Leukopenia (10^9/L)	2.5–3.5	1.5–2.49	1.0–1.49	<1.0
Thrombocytopenia (10^9/L)	75–99	50–74	25–49	<25
**Blood Chemistry**				
Liver Function – ALT, AST	1.25–2.5×ULN*	2.6–5×ULN	5.1–10×ULN	>10×ULN
Creatinine	1.1–1.5×ULN	1.6–3.0×ULN	3.1–6×ULN	>6×ULN
BUN	1.25–2.5×ULN	2.6–5×ULN	5.1–10×ULN	>10×ULN
Bilirubin: Elevated due to factors with normal liver function	1.1～1.5×ULN	1.6～2.0×ULN	2.0～3.0×ULN	＞3.0×ULN
Bilirubin: Elevated due to factors with abnormal liver function	1.1～1.25×ULN	1.26～1.5×ULN	1.51～1.75×ULN	＞1.75×ULN
CK and Isoenzymes	1.25～1.5×ULN	1.6～3.0×ULN	3.1～10×ULN	＞10×ULN
Troponin	1.25～1.5×ULN	1.6～3.0×ULN	3.1～10×ULN	＞10×ULN
**Urine**				
Glucose (mmol/L)	≤15	15.1～20	20.1～30	＞30
Protein (g/L)	0.16～0.3	0.31～0.75	0.76～1.0	＞1.0
Red Blood Cells (cells/μL)	25～80	81～200	＞200	Gross hematuria
**Local Reactions and Skin Manifestations**				
Injection Site Pain	Mild pain, no treatment or occasional non-prescription medication required	Moderate pain, regular non-prescription medication or occasional prescription medication required	Severe pain, repeated prescription medication required	Hospitalization required

**DNA Vaccine**	Injection Site Erythema	Diameter <1.5 cm	Diameter 1.5–3 cm	Diameter >3 cm	Gangrene or exfoliative dermatitis
	Injection Site Blisters and Ulcers	Blisters or ulcers at injection site, diameter <1 cm	Blisters or ulcers at injection site, diameter 1–2 cm, heals within 2 weeks	Blisters or ulcers at injection site, does not heal for over 2 weeks	Necrosis
	Injection Site Induration	Diameter <1.5 cm	Diameter 1.5–3 cm	Diameter >3 cm	Gangrene or exfoliative dermatitis
	Injection Site Papules	Diameter <1.5 cm	Diameter 1.5–3 cm	Diameter >3 cm	
	Injection Site Pustules	Diameter <0.5 cm	Diameter 0.5–1 cm	Diameter >1 cm, no tendency to heal for 2 weeks	Necrosi
	Injection Site Swelling	Diameter <1.5 cm, no impact on activity	Diameter 1.5–3 cm or impacts activity	Diameter >3 cm or restricts daily activities	Gangrene
	Generalized Rash	Scattered maculopapular rash (<5 areas), no itching or other symptoms	Scattered macules or papules (>5 areas), itching or other symptoms, no treatment required	Diffuse macules or papules, or exudative rash, requires medication	Rash involving mucous membranes, exfoliative dermatitis, or erythema multiforme, or Stevens-Johnson syndrome
**rTV vaccine**	Injection Site Papules	Diameter 1.1–1.5 cm	Diameter 1.6–3 cm	Diameter >3 cm	
	Injection Site Swelling or Redness	Diameter 3–5 cm	Diameter 5.1–10 cm, improves within 5 days	Diameter 5.1–10 cm, persists for over 5 days, or diameter >10 cm	
	Injection Site Induration	Diameter 1.1–1.5 cm	Diameter 1.6–3 cm	Diameter >3 cm	
	Injection Site Blisters	Diameter 1.1–1.5 cm	Diameter 1.6–3 cm	Diameter >3 cm	
	Injection Site Pustules	Diameter 1.1–1.5 cm	Diameter 1.6–3 cm	Diameter >3 cm	Pustules continue to expand without scabbing after 2 weeks; gangrene or exfoliative dermatitis

	Injection Site Erythema	Diameter <1.5 cm	Diameter 1.5–3 cm	Diameter >3 cm	Gangrene or exfoliative dermatitis
**Systemic Symptoms**					
Headache		Does not affect activity, no treatment required	Transient, affects daily activities, requires treatment (non-narcotic analgesics)	Severely affects daily activities, occasional narcotic treatment required	Persistent, requires repeated narcotic treatment
Chills		Occasional OTC non-steroidal antipyretics required	Limits daily activities >6 hours, or requires repeated non-steroidal antipyretics	Severely affects daily activities, requires prescription medication	Hospitalization required
Fatigue		Reduced normal activity <48 hours	Reduced normal activity 20%–50% >48 hours	Severely affects daily activities, reduces normal activity >50%, unable to work	Unable to care for self, emergency or hospitalization
Myalgia		Does not affect daily activities	Tenderness in non-injection site muscles, slightly limits daily activities	Severe muscle tenderness, severely affects daily activities	Severe symptoms, muscle necrosis, emergency or hospitalization
Arthralgia		Mild pain, does not limit daily activities	Moderate pain, slightly limits daily activities	Severe pain, severely affects daily activities	Hospitalization required
Nausea		Mild or transient, does not affect normal eating	Affects normal eating	Unable to eat, requires outpatient IV fluids	Hospitalization required
Vomiting		1 episode in 24 hours	2–5 episodes in 24 hours	>6 episodes in 24 hours or IV fluids required	Requires hospitalization or alternative nutrition
Diarrhea		Mild or transient, 2–3 episodes/day, lasts <1 week	Moderate or persistent, 4–5 episodes/day, or diarrhea >1 week	>6 episodes/day, or bloody stools, orthostatic hypotension, electrolyte imbalance, requires >2L IV fluids in 24 hours	Hypotensive shock, requires hospitalization
Cough		Transient, no treatment required	Paroxysmal cough, treatment effective	Persistent cough, treatment ineffective	Emergency or hospitalization
Allergic Reaction		Itching without rash	Localized urticaria	Generalized urticaria, angioedema	Severe allergic reaction, or post-vaccination

				encephalitis
Lymphadenopathy	Newly appeared ipsilateral axillary lymphadenopathy, multiple nodes possible, largest single node diameter ≤2 cm; may be tender but does not affect daily activities	Newly appeared ipsilateral axillary lymphadenopathy, plus one of the following: ① New lymphadenopathy in 1–2 other lymph node regions; ② Largest single node diameter 2–3 cm; ③ Slightly limits daily activities	Newly appeared ipsilateral axillary lymphadenopathy, plus one of the following: ① New lymphadenopathy in ≥3 other lymph node regions; ② Largest single node diameter >3 cm; ③ Severe tenderness, severely affects daily activities	—
Other Discomfort	Does not affect daily activities	Slightly affects daily activities, no medication required	Severely affects daily activities, requires medication	Requires hospitalization
**Vital Signs***				
Fever, Axillary Temperature	37.3–37.9°C	38.0–39.0°C	>39.0°C	
Tachycardia (beats/min)	101–115	116–130	>130, or other rapid arrhythmias	Emergency or hospitalization due to arrhythmia
Bradycardia (beats/min)	50–54	45–49	<45	Emergency or hospitalization due to arrhythmia
Hypertension (Systolic BP, mmHg)**	141–150	151–155	>155	Emergency or hospitalization due to severe hypertension
Hypertension (Diastolic BP, mmHg)**	91–95	96–100	>100	Emergency or hospitalization due to severe hypertension
Hypotension (Systolic BP, mmHg)**	85–89	80–84	<80	Emergency or hospitalization due to hypotensive shock
Hypertension (Diastolic BP, mmHg)**	91–95	96–100	>100	Emergency or hospitalization due to severe hypertension

Note： ULN: Upper Limit of Normal

* Cited from the “Chinese Vaccination Manual”, subjects should be tested in a resting state.

** The determination of abnormal blood pressure should be compared with the baseline blood pressure before vaccine administration for specific analysis.

Annex 3. Subject Diary Card

Notes for completing this card:

1) Every day, complete the diary card within 2 weeks after DNA vaccination and 4 weeks after rTV vaccination, and record any discomforts occurring at other times on the dairy card.
2) Please record the non-prescription drugs or other treatments to alleviate local reactions;
3) If you have any symptoms that cannot go away by themselves, please contact the study staff;
4) After you have completed the diary card on the day, please give the completed dairy to the study staff at your next visit.
5) For safety, after rTV vaccination, subjects themselves may not remove the gauze covering the vaccination site without special circumstances

Diary Card

Screening number □□□□□□ Subject initials □□□□
Recording date 20 Year Month Day Day after vaccination (day 1-28)

Axillary temperature:

A.M. (6:00- 8:00) ℃ P.M. (8:00- 10:00) ℃

Have you participated in sports or heavy physical labor? Yes No

Type of sports or heavy physical labor:

Duration: hours

Please record the physical discomfort after vaccination:

Name of symptom	Duration of symptom	Time to relieve

Please record the medications after vaccination:

Drug name	Reason for medication	Time of starting medication	Dosage of medication	Route of administration	Time of ending medication
				1. Oral; 2. intramuscular injection; 3. Intravenous; 4. Topical	

				1. Oral; 2. intramuscular injection; 3. Intravenous; 4. Topical	

Subject's signature: Date：□□□□Year□□Month□□Day

Report form of treatment of systemic and local reactions (including non-prescription drugs)

NO.	Drug name	Dosage	Times per day	Duration of use (from Month Day to Month Day)	Reason for using the drug and the effect
1					
2					
3					
4					
5					
**If other treatments are used, please explain them specifically**					

Annex 4. Summary of Test Results for HIV-1 DNA Vaccine

Test Item	Test Method	Quality Standard	pGP140 (20041123)	PGPNEF (20041124)
**Appearance**	Visual inspection	Clear, colorless liquid, free of foreign matter	Pass	Pass
**pH Value**	*Chinese Pharmacopoeia*	6.7–7.7	Pass	Pass
**Fill Volume**	*Chinese Pharmacopoeia*	Average fill volume not less than the labeled volume (1.0 ml/vial), each vial not less than 93% of the labeled volume	Pass	Pass
**Sterility Test**	*Chinese Pharmacopoeia*	No microbial growth	Pass	Pass
**Identification Test (Restriction Enzyme Mapping)**	Restriction enzyme mapping	Consistent with expected results	Pass	Pass
**Residual E. coli Genomic DNA**	Solid-phase slot blot hybridization	≤2 μg/mg plasmid	Pass	Pass
**Residual Host Cell Protein**	*Chinese Biological Products Regulation*	≤1 μg/mg plasmid	Pass	Pass
**Endotoxin Content**	*Chinese Pharmacopoeia*	≤5 EU/mg plasmid	Pass	Pass
**Residual RNA (Agarose Gel Electrophoresis)**	Agarose gel electrophoresis	No other bands or diffuse nucleic acid bands except plasmid band	Pass	Pass
**Purity Test (UV Absorption)**	UV absorption	A260/A280 ≥ 1.75	Pass	Pass
**Concentration Test (UV Absorption)**	UV absorption	1.8–2.2 mg/ml	Pass	Pass
**Plasmid Conformation Test (Agarose Gel Electrophoresis)**	Agarose gel electrophoresis	Circular plasmid ≥ 90%	Pass	Pass
**Residual LiCl**	ICP-MS	≤1 μg/mg plasmid	Pass	Pass
**In Vitro Gene Expression Test (Western Blot)**	Western Blot	pGP140: Specific band at 130 kDa; pGPNEF: Specific band at 160 kDa	Pass	Pass
**Abnormal Toxicity Test**	*Chinese Biological Products Regulation*	Guinea pig test: Guinea pigs survive and gain weight; Mouse test: Mice survive and gain weight	Pass	Pass

**Humoral Immunogenicity Test (ELISA)**	ED50 ELISA	ED50 ≤ 100 μg (pGP140); ED50 ≤ 80 μg (pGPNEF)	Pass	Pass
**Cellular Immunogenicity Test (ELISPOT)**	ELISPOT	Positive cellular immune response rate in mice vaccinated with DNA vaccine ≥ 60%	Pass	Pass

Annex 5. Summary of Test Results of Recombinant Tiantan Vaccinia (rTV) AIDS Vaccine

Test Item	Test Method	Quality Standard	Results
**Appearance**	Visual inspection	Light brown viscous liquid	Pass
**pH Value**	*Chinese Pharmacopoeia*	7.2 ± 0.5	7.02
**Fill Volume**	*Chinese Pharmacopoeia*	Average fill volume not less than the labeled volume, each vial not less than 93% of the labeled volume	Pass
**Sterility Test**	*Chinese Pharmacopoeia*	Negative	Pass
**Viral Titer**	Hemadsorption method	$5.0 \times 10^6 – 3.0 \times 10^7$ PFU/ml	Pass
**Abnormal Toxicity Test**	*Chinese Biological Products Regulation*	Animals survive for 5 days with no local ulcers	Pass
**Rabbit Virulence Test**	*Chinese Biological Products Regulation* (1979 edition)	Necrosis diameter at 10^{-1} and 10^{-2} dilutions should not exceed 10 mm in total; no necrosis at other dilutions. Local redness or swelling at all 5 dilutions should be less than 70 mm in total.	Pass
**Bacterial Endotoxin**	*Chinese Biological Products Regulation* (2000 edition)	Endotoxin content < 500 EU/ml, < 10 EU per dose	Pass
**Rabbit Virulence Test**	*Chinese Biological Products Regulation* (1979 edition)	Necrosis diameter at 10^{-1} and 10^{-2} dilutions should not exceed 10 mm in total; no necrosis at other dilutions. Local redness or swelling at all 5 dilutions should be less than 70 mm in total.	Pass
**Bacterial Endotoxin**	*Chinese Biological Products Regulation* (2000 edition)	Endotoxin content < 500 EU/ml, < 10 EU per dose	Pass
**HIV Target Gene**	1. PCR<br>2. Western Blotting	1. gagpolΔ fragment should be around 2.9 kb; gp140TM should be around 2.1 kb. gp140TM digested with StuI yields 1170 bp and 975 bp fragments; digested with NdeI yields 176 bp and 1969 bp fragments. gagpolΔ digested with PstI yields 617 bp and 2271 bp fragments; digested with SpeI yields 709 bp and 2079 bp fragments.<br>2. gagpolΔ gene-specific band at 55 kDa, sometimes	Pass

		also 24 kDa and 41 kDa bands. gp140TM gene-specific band at 140 kDa, sometimes degraded bands below 140 kDa.	
**Humoral Immunogenicity Test (ELISA)**	ED50 ELISA	ED50 < 8.0 × 10^6 PFU	Pass
**Cellular Immunogenicity Test**	Intracellular IFN-γ secretion by flow cytometry	Positive if ≥ 50% higher than the mean of the negative control group; vaccination group positive rate ≥ 60%	Pass

Annex 6. Reactions after Smallpox Vaccination and Principles of Treatment

As this is the first clinical study of rTV vaccine with Tiantan strain of vaccinia virus as a vector, the vaccination reactions caused by the vaccine can be determined only after completion of this clinical trial, and the reactions caused by Tiantan strain of vaccinia virus for smallpox vaccination can be regarded as a reference for expected adverse reactions. However, when constructing the rTV vaccine, the insertion of HIV genes has significantly reduced the virulence of Tiantan strain of vaccinia virus. The experiments of animals have also shown that the toxicity of rTV vaccine in mice is 100-1000 times lower than that of Tiantan vaccinia virus, therefore, the adverse reactions caused by AIDS vaccine should be much lower than those caused by Tiantan strain smallpox vaccine.

Reactions in human body after smallpox vaccination include general reaction and abnormal reactions. The general reactions are typical local reactions of smallpox and possibly some systemic symptoms after vaccination. An abnormal reaction refers to a rarely serious adverse reaction after vaccination. Tiantan strain vaccinia virus as a smallpox vaccine has been universally vaccinated in Chinese population, and it has played a good role in the prevention of smallpox epidemic, and successfully eradicates smallpox disease in China. Different from the crude smallpox vaccine from tissues such as cow skin used in other countries, Tiantan strain smallpox vaccine is a refined vaccine obtained by cell culture, therefore, it has lower rate of abnormal reactions after vaccination than other smallpox vaccines, with the incidence of approximately 5.6/million for post-vaccination encephalitis, progressive pox, chicken pox rash, generalized pox and pleomorphic smallpox, and 80% of these reactions occur in infants under 1 year of age and immunocompromised persons. There is no report of deaths after vaccination of cell-cultured Tiantan strain smallpox vaccine manufactured by Beijing Institute of Biological Products Co., Ltd. The general reactions and abnormal reactions developed after smallpox vaccination as well as treatment principles are listed by referring to Compilation of Data from Experience Exchange Meeting on Vaccinia in 1974 [35] in China and the data from U.S. CDC on the New York strain smallpox vaccination [36].

I. General reactions after vaccination

(I) Typical local reactions after vaccination

After the successful smallpox vaccination, there are generally papules, blisters, pustules, crusting and scab falling off at the injection site, and also small lesions papules and blisters (called sub-pox) can be seen around the damaged skin, accompanied by localized itching and pain.

Typical local reactions after smallpox vaccination occur at the following time:

Time After accination	Typical Vaccination Reaction
**Day 3–4**	A papule appears at the vaccination site.
**Day 5–6**	The papule develops into a vesicle (blister) with a surrounding red halo, and the top of the vesicle becomes umbilicated (indented).
**Day 8–9**	The vesicle turns into a pustule, reaching its maximum size between days 8 and 10.
**Day 12+**	The pustule gradually dries and scabs begin to form.
**Day 17–21**	The scab falls off, leaving a permanent scar.

(II) Common systemic signs and symptoms after vaccination

Systemic symptoms after smallpox vaccination and their occurrence rates are: fever (30%), headache (40%), myalgia (20%), nausea (20%), fatigue (50%) and regional lymph node enlargement (50 %). These signs and symptoms usually go away within 2 weeks after vaccination by themselves or by only symptomatic treatment.

II. Abnormal reactions after vaccination and principles of treatment

(I) Concurrent bacterial infection at vaccination site

1. Clinical manifestations

The abnormal reactions are common in immunocompromised persons. They often occur within 2 to 3 weeks after vaccination, and include induration, swelling, increased skin temperature and pain at the vaccination site, as well as swollen local lymph nodes and systemic infection symptoms.

1. Treatment

Streptococcus or Staphylococcus aureus is the most important pathogen, but these reactions may also be caused by other bacterial infections. The culture should be done for tissues or blood, and the drug susceptibility testing should be conducted. The infection will be managed by use of antibiotics.

(II) Transplanted pox

1. Clinical manifestations

It is seen in preschool and school-age children. This is caused by scratching the vaccination site using hands of those who were vaccinated, leading to virus heterotopic transplant, or by recently close contact with a person vaccinated with smallpox, leading to accidental infection. Transplanted pox can occur at near the vaccination or at other parts of body including eyes, mouth, lips, tongue, nose, back, anus and genitalia, etc.

The symptoms are more severe in persons with poxes transplanted into the eyes, and are not so serious in persons with poxes transplanted to other parts of body. Generally, the diseases of eye pox virus infection occur 5 to 12 days after vaccination. The symptoms are most common in eyelids: obviously swelling of eyelids, poxes of different sizes in eyelids or ruptured poxes to form shallow ulcers. Eye fissure is often sealed by a lot of secretions, and the eyes cannot be opened. It is often accompanied by significant conjunctival hyperemia, and can cause keratitis, iritis and the like. If not treated early, it may lead to sequela, such as loss of eyelashes, eyelid scarring, corneal scarring and etc., and can also affect vision, in severe cases, can cause blindness.

2. Prevention and treatment

The main measures for vaccinated persons or those in close contact with the vaccinated to avoid transplant poxes are: ① Avoid touching or scratching the vaccination site; ② Do not rub your eyes after contact with the vaccinated person, and often wash your hands with warm soapy water or 60% alcohol sanitizer; ③ Cover the vaccination site with breathable gauze or a bandage until the scab falls off, and the cover should be changed often to prevent leakage of

exudate. The vaccinated person should wear long-sleeved clothes as a second layer for isolation; ④ Place the contaminated cover in a sealed plastic bag before disposal, and your clothing having contacted with the vaccination site should be cleaned using hot water and liquid detergent.

As for general transported pox, no special treatment is needed, but preventing the skin infections. Transplanted poxes at eyes should be treated as soon as possible, with principles as follows: ① If the vaccine accidentally splashes into the eyes, you must not rub with your hands, and should immediately flush several times with saline (or water), and then use idoxuridine eye drops, several times a day; ② Avoid scratching using your hands, and administer routine antibiotics to prevent bacterial infections. ③ Gamma-globulin eye-drops containing high titers of vaccinia virus antibodies can be simultaneously used, but should not be used in patients with keratitis.

(III) Progressive pox (gangrene pox)

1. Clinical manifestations

It is seen in patients with defects in immune function, particularly in cellular immunity. It is characterized by progressive painless gangrene at injection site. Poxes have no scab formation two weeks after vaccination, continue to increase in size, and are not healed after a long term. Ulcers are expanding and deepening with significant central necrosis, forming a thick black eschar. Local inflammatory reactions are mild, and the similar pock lesions can also in other parts of the body. It is also accompanied by symptoms such as fever and systemic failure. If not treated, it will lead to a mortality rate.

2. Treatment

Early diagnosis and early treatment are very important. ① Keep dry at the lesion site, use antibiotics to control or prevent infections, and perform surgical treatment for burn wounds. ②Immunoglobulin containing high titers of vaccinia antibodies should be given in a timely manner. ③ Use of thiosemicarbazone and topical application of idoxuridine (IDU); ④ Systemic supportive therapy.

(IV) Systemic pox

1. Clinical manifestations

It is found in persons with low immunity or slow response, or those with poor resistance after an illness. At 6 to 14 days after vaccination, a number of poxes appear on skin all over the body, and they develop faster than the primary ones through four stages, i.e., papules, blisters, pustules and scabs. Poxes have a shape like umbilical concave with hard base, accompanied by systemic symptoms. It occurs only once in most of patients, and more than once in a small number of patients. After the scabs fall off, they form shallow scars, which can go away later.

2. Treatment

① Immunoglobulin containing high titers of vaccinia antibodies should be given in a timely manner; the treatment should be given by use of thiosemicarbazone and topical

application of idoxuridine (IDU); ② Prevent secondary infection; ③ Symptomatic treatment.

(V) Eczema pox

1. Clinical manifestations

It is seen in patients with eczema, and occurs after vaccination or after close contact with vaccinated persons. It is characterized by multiple poxes with different sizes and often fused into a patch at the site of eczema or on normal skin, and it may have systemic symptoms such as high fever and listlessness. At this time point, the original eczema exacerbates, and it is often prone to secondary infection locally. The eczema pox is more severe in those with this condition caused by the contact with than in those who with this condition caused by vaccination.

2.Treatment

① Immunoglobulin containing high titers of vaccinia antibodies should be given in a timely manner; ② Keep local region dry and clean to prevent secondary infection, and give antimicrobial therapy in a timely manner in case of any infections; ③ Systemic supportive therapy, maintaining the balance of water and electrolyte; ④ Symptomatic treatment.

(VI) Allergic polymorphous rash

1. Clinical manifestations

It is caused by the allergies to the ingredients of vaccine. It often occurs in 2 to 5 days (also up to 2 weeks) after vaccination. The skin rashes have various shapes, including erythema, papules, maculopapulaes and urticaria, and one of them is the main in most cases. It can be accompanied by itching. Skin rashes often start from the face and then to all over the body, and can also involve oral mucosa. They can go away by themselves in 1 to 4 days. n severe cases, there may be blisters. The skin rashes are often accompanied by systemic symptoms such as fever. The blisters can be fused and have irregular shapes and different sizes. The surface is flat without depression, and its base presents the burn-like red erosion after rupture. A thin scab will be formed, and there will be no scar after the scab falls off.

Allergic polymorphous rash can also progress to Stevens-Johnson syndrome (SJS). It is mainly manifested as severe erythema multiforme, and can involve skin and mucous membranes.

2. Treatment

No special treatment is required for the mild condition. The severe cases can be treated by anti-allergy treatment such as chlorpheniramine, prednisone, etc., and the infection can be managed by use of antibiotics. Severe blister polymorphous rashes can be treated by supportive therapy using immunoglobulin containing high titers of vaccinia antibodies.

(VII) Purpura after vaccination

1. Clinical manifestations

It is the toxicity or allergic reaction of vaccinia. At 7 to 10 days after vaccination, the

bleeding points with the size of needle tip are present on skin. They will gradually expand in different shapes and sizes, and can be fused into a patch. They are not extruding the skin, and are dark and purple in color. It can occur once or more. In severe cases, there may be hematuria, blood in the stool, and internal bleeding. The platelets are normal or decreased, and the blood clotting time was normal or slightly longer. It is often accompanied by systemic symptoms.

2. Treatment

(1) Anti-allergy treatment; (2) Use of hemostatic agents, and blood transfusion if necessary; (3) Trial use of immunoglobulin containing high titers of vaccinia antibodies.

(VIII) Encephalitis after vaccination

1. Clinical manifestations

It is seen in infants or those who are old or physically weak. It is manifested as symptoms of central nervous system damage such as high fever, headache, convulsions, vomiting, unconsciousness and increased intracranial pressure at 9 to 15 days after vaccination. Meningeal irritation sign and pathological reflex are positive. Cerebrospinal fluid pressure may increase, and the number of cells and proteins can be slightly increased. Generally, it will be gradually restored after about one week, and it can cause death in severe cases. Encephalitis caused by other infections or poisoning should be excluded.

2. Treatment

(1) Symptomatic and supportive treatments such as antispasmodic administration, cooling, and maintaining the balance of water and electrolyte; dehydrating agent will be given to those with significantly increased intracranial pressure and cerebral edema. (2) Drugs to promote the restoration of nerve cells and to improve the metabolism of nerve tissues should be given, such as VB12 and so on. (3) Use of immunoglobulin containing high titers of vaccinia antibodies is not recommended.

(IX) Encephalomyelitis after vaccination

1. Clinical manifestations

It is seen in infants or those who are old or physically weak. The latent period is 8 to 15 days. It is manifested as headache, vomiting and systemic discomfort, and it can develop into amnesia, delirium, disorientation, agitation, confusion, drowsiness, seizures and coma, accompanied by incontinence or urinary retention, obstinate constipation; Patients with this disease often have mutism, dysphagia and unintentional acts, and the spinal cord can be involved.

2. Treatment

Intensive care, supportive therapy and symptomatic treatment.

Annex 7. Documents Stored for the Clinical Trial

I. Preparation stage of clinical trial

Document		Investigator	Sponsor
1	Investigator's Brochure	Retain	Retain
2	Signed Protocol and Amendments	Retain original	Retain
3	Case Report Form (Sample)	Retain	Retain
4	Informed Consent Form	Retain original	Retain
5	Financial Agreement	Retain	Retain
6	Signed Multi-Party Agreement (Investigator, Sponsor, CRO)	Retain	Retain
7	Ethics Committee Approval	Retain original	Retain
8	Ethics Committee Membership List	Retain original	Retain
9	Clinical Trial Application		Retain original
10	Preclinical Laboratory Data		Retain original
11	National Medical Products Administration (NMPA) Approval	Retain	Retain original
12	Investigator's Curriculum Vitae and Related Documents	Retain	Retain original
13	Normal Range for Laboratory Tests Related to the Clinical Trial	Retain	Retain
14	Quality Control Certificates for Medical or Laboratory Procedures	Retain original	Retain
15	Labels for Investigational Product		Retain original
16	Shipping Records for Investigational Product and Related Materials	Retain	Retain
17	Certificate of Analysis for Investigational Product		Retain original
18	Unblinding Procedure for Blinded Trials		Retain original
19	Master Randomization List		Retain original
20	Monitoring Reports		Retain original

II. Conducting stage of clinical trial

Document		Investigator	Sponsor
21	Updated Investigator's Brochure	Retain	Retain
22	Updates to Other Documents (Protocol, Case Report Form, Informed Consent Form, Written Notifications)	Retain	Retain
23	Curriculum Vitae of New Investigators	Retain	Retain original
24	Updates to Normal Ranges for Medical and Laboratory Tests/Procedures	Retain	Retain

25	Shipping Records for Investigational Product and Related Materials	Retain	Retain
26	Certificate of Analysis for New Batches of Investigational Product		Retain original
27	Monitor's Visit Reports		Retain original
28	Signed Informed Consent Forms	Retain original	
29	Original Medical Records	Retain original	
30	Completed, Signed, and Dated Case Report Forms	Retain original	Retain copy
31	Investigator's Serious Adverse Event Reports to the Sponsor	Retain original	Retain
32	Sponsor's Unexpected Serious Adverse Drug Reaction Reports to Regulatory Authorities and Ethics Committees	Retain	Retain original
33	Interim or Annual Reports	Retain	Retain
34	Subject Identification Code List	Retain original	
35	Subject Screening and Enrollment Logs	Retain	Retain
36	Site Investigational Product Accountability Records	Retain	Retain
37	Investigator's Signature Samples	Retain	Retain

III. After completion of the clinical trial

Document		Investigator	Sponsor
38	Certificate of Destruction of Investigational Product	Retain	Retain
39	Subject Identification Code List for Completed Trials	Retain	Retain
40	Audit Certificate		Retain original
41	Final Monitoring Report		Retain original
42	Treatment Allocation and Unblinding Documentation		Retain original
43	Trial Completion Report (to Ethics Committee and National Medical Products Administration)		Retain original
44	Final Study Report	Retain	Retain original

Annex 8. Serious Adverse Events (SAEs) Report Form

Approval No. of Phase Ib clinical study of AIDS vaccine: 2008L04306 Original number: 11050199

<table>
<tr><td colspan="2">Type of report</td><td colspan="3">□ Initial report □ Follow up report □ Summary report</td><td colspan="2">Reporting time:
Year Month Day</td></tr>
<tr><td colspan="2">Name of medical institution and specialties</td><td colspan="3">Peking Union Medical College Hospital, Clinical Infectious Diseases Specialty.</td><td colspan="2">Tel：123450678</td></tr>
<tr><td colspan="2">Name of sponsor(s)</td><td colspan="3">National Center for AIDS/STD Control and Prevention, Chinese Center for Disease Control and Prevention, Beijing Institute of Biological Products Co., Ltd.</td><td colspan="2">Tel：630307608</td></tr>
<tr><td colspan="2" rowspan="4">investigational vaccine</td><td rowspan="2">Phase Ia</td><td colspan="4">Name in Chinese: 重组天坛株痘苗病毒疫苗</td></tr>
<tr><td colspan="4">Name in English: Recombinant Vaccine Tiantan</td></tr>
<tr><td rowspan="2">Phase Ib,</td><td colspan="4">Name in Chinese: 艾滋病疫苗（核酸与重组天坛痘苗联合使用）</td></tr>
<tr><td colspan="4">Name in English: AIDS Vaccinia (Combined use of DNA Vaccine and Recombinant Vaccine Tiantan)</td></tr>
<tr><td colspan="2">Vaccine registration classification and dosage form</td><td colspan="5">Registration classification: prophylactic biological product Dosage form: Injection</td></tr>
<tr><td colspan="2">Clinical study classification</td><td colspan="3">☐ Phase Ia clinical observation
☐ Phase Ib clinical observation</td><td colspan="2">Clinical indications: Low- and high-risk populations of HIV infection</td></tr>
<tr><td rowspan="2">Basic information about subject</td><td>Initials:</td><td>Date of birth:</td><td>Gender:
□ Male □ Female</td><td>Height (cm):</td><td colspan="2">Weight (Kg):</td></tr>
<tr><td colspan="6">Concomitant diseases and treatment: □ Yes □ No
1. Disease: Treatment medication: Dosage and administration:
2. Disease: Treatment medication: Dosage and administration:
3. Disease: Treatment medication: Dosage and administration:</td></tr>
<tr><td colspan="2">SAE's medical terminology (diagnosis)</td><td colspan="5"></td></tr>
<tr><td colspan="2">SAE situation</td><td colspan="5">□ Death Year Month Day
□ Leading to hospitalization
□ Leading to prolonged hospitalization</td></tr>
</table>

<table>
<tr><td></td><td colspan="2">□ Disability □ Dysfunction
□ Leading to congenital malformation
□ Life-threatening □ Other</td></tr>
<tr><td colspan="2">Date of SAE onset: Year Month Day</td><td>Date of informing investigator of SAE:
Year Month Day</td></tr>
<tr><td>Actions taken for investigational vaccine</td><td colspan="2">□ Continuing with vaccination
□ Reducing the dosage
□ Suspending and then resuming
□ Stopping vaccination</td></tr>
<tr><td>SAE outcome</td><td colspan="2">□ Symptom goes way (Sequelae □ Yes □ No)
□ Symptom persists</td></tr>
<tr><td>Relationship between SAE and investigational vaccine</td><td colspan="2">□ Definitely related □ Possibly related
□ Possibly unrelated □ Definitely unrelated
□ Undeterminable</td></tr>
<tr><td>SAE reporting</td><td colspan="2">China: □ Yes □ No □ Unknown;
Outside China: □ Yes □ No □ Unknown</td></tr>
<tr><td colspan="3">Details of SAE occurrence and treatment:</td></tr>
</table>

Name of reporting institution:

Position/title of reporter: Signature of reporter:

References

1. 2007 AIDS epidemic update. http://data.unaids.org/pub/EPISlides/2007/2007_epiupdate_en.pdf
2. 《中国艾滋病防治联合评估报告》 2007 年 12 月 p7
3. The impact of HIV & AIDS on Africa. http://www.avert.org/aidsimpact.htm
4. Ruprecht R M, Baba T W, Liska V. Attenuated HIV vaccine: caveats. Science, 1996;271:1790-2.
5. Ruprecht R M. Live attenuated AIDS viruses as vaccines: promise or peril? Immunol Rev, 1999;170:135-49.
6. Baba T W, Liska V, Khimani A H, et al. Live attenuated, multiply deleted simIan immunodeficiency virus causes AIDS in infant and adult macaques. Nat Med, 1999;5:194-203.
7. Keefer M. San Francisco C A Infectious Diseases Society of America Annual Meeting. 1995.
8. Rubinstein A, Goldstein H, Pettoello-Mantovani M, et al. Safety and immunogenicity of a V3 loop synthetic peptide conjugated to purified protein derivative in HIV-seronegative volunteers. Aids, 1995;9:243-51.
9. Naylor P H, Sztein M B, Wada S, et al. Preclinical and clinical studies on immunogenicity and safety of the HIV-1 p17-based synthetic peptide AIDS vaccine--HGP-30-KLH. Int J Immunopharmacol, 1991;13: S117-27.
10. Cohen J. Clinical research. A setback and an advance on the AIDS vaccine front. Science, 2003;300:28-9.
11. Donnelly J J, Ulmer J B, Shiver J W, et al. DNA vaccines. Annu Rev Immunol, 1997; 15:617-48.
12. W.Jaoko,G.Omosa K B. Safety and immunogenicty of DNA and MVA HIVA Vaccines in phase I HIV-1 vaccine trIals in Nairobi,Kenya. AIDS Vaccine04 Conference Lausanne Switzerland. WWW.AIDSVaccine04.org.
13. Pal R, Venzon D, Letvin N L, et al.ALVAC-SIV-gag-pol-env-based vaccination and macaque major histocompatibility complex class I (A*01) delay simIan immunodeficiency virus SIVmac-induced immunodeficiency. J Virol, 2002; 76:292-302.
14. AIDS Vaccine Evaluation Group 022 Protocol Team. Cellular and humoral immune responses to a canarypox vaccine containing human immunodeficiency virus type 1 Env, Gag, and Pro in combination with rgp120. J Infect Dis, 2001;183:563-70.
15. Bures R, Gaitan A, Zhu T, et al. Immunization with recombinant canarypox vectors expressing membrane-anchored glycoprotein 120 followed by glycoprotein 160 boosting fails to generate antibodies that neutralize R5 primary isolates of human immunodeficiency virus type 1. AIDS Res Hum Retroviruses, 2000;16:2019-35.
16. Check E, Army HIV vaccine to undergo clinical trIal as rival is halted. Nature, 2002;416:6.
17. Benson J, Chougnet C, Robert-Guroff M, et al. Recombinant vaccine-induced protection against the highly pathogenic simIan immunodeficiency virus SIV (mac251): dependence on route of challenge exposure. J Virol, 1998; 72:4170-82.
18. Results from EV01 HIV Vaccine trIal, London and Lausanne, June 7th, 2004.http://www.eurovac.net/EV01Results.pdf.
19. IAVI report. http://www.Iavireport.org/specIals/OngoingTrIalsofPreventiveHIVVaccines.pdf
20. Shiver J W, Davies M E, Perry H C, et al. Humoral and cellular immunities elicited by HIV-1 vaccination. J Pharm Sci, 1996;85:1317-24.
21. Amara R R, Villinger F, Altman J D, et al. Control of a mucosal challenge and prevention of AIDS by a multiprotein DNA/MVA vaccine. Vaccine, 2002; 20:1949-55.
22. Robinson H L, DNA vaccines for immunodeficiency viruses. Aids, 1997; 11: S109-19.
23. Boyer J D, Ugen K E, Wang B, et al. Protection of chimpanzees from high-dose heterologous HIV-1 challenge by DNA vaccination. Nat Med, 1997;3:526-32.
24. Boyer J D, Wang B, Ugen K E, et al. In vivo protective anti-HIV immune responses in non-human primates through DNA immunization. J Med Primatol, 1996; 25:242-50.

25. Boyer J D, Cohen A D, Vogt S, et al. Vaccination of seronegative volunteers with a human immunodeficiency virus type 1 env/rev DNA vaccine induces antigen-specific proliferation and lymphocyte production of beta-chemokines. J Infect Dis, 2000; 181:476-83.
26. Amara R R, Villinger F, Staprans S I, et al. Different patterns of immune responses but similar control of a simIan-human immunodeficiency virus 89.6P mucosal challenge by modified vaccinIa virus Ankara (MVA) and DNA/MVA vaccines. J Virol, 2002;76:7625-31.
27. Chernos V I, ChelIapov N V, Antonova T P, et al. Verification of the safety, inoculability, reactogenicity and antigenic properties of a live recombinant smallpox-hepatitis B vaccine in an experiment in volunteers. Vopr Virusol, 1990;35:132-5.
28. Gu S Y, Huang T M, Ruan L, et al. First EBV vaccine trIal in humans using recombinant vaccinIa virus expressing the major membrane antigen. Dev Biol Stand, 1995;84:171-7.
29. Baldwin P J, van der Burg S H, Boswell C M, et al. VaccinIa-expressed human papillomavirus 16 and 18 e6 and e7 as a therapeutic vaccination for vulval and vaginal intraepithelIal neoplasIa. Clin Cancer Res, 2003;9:5205-13.
30. Polacino P, Stallard V, Klaniecki J E, et al. Limited breadth of the protective immunity elicited by simIan immunodeficiency virus SIVmne gp160 vaccines in a combination immunization regimen. J Virol, 1999;73:618-30.
31. Cooney E L, Collier A C, Greenberg P D, et al. Safety of and immunological response to a recombinant vaccinIa virus vaccine expressing HIV envelope glycoprotein. Lancet, 1991;337:567-72.
32． The achievement of global eradication of smallpox: final report of the global commission for the certification of smallpox eradication. Geneva 1979 Dec: 48
33． Frey SE, Couch RB, Tacket CO，et al. Clinical responses to undiluted and diluted smallpox vaccine. N Engl J Med. 2002; 346: 1265-74.
34. Talbot TR, Stapleton JT, Brady RC, et al. Vaccination success rate and reaction profile with diluted and undiluted smallpox vaccine: a randomized controlled trIal. JAMA. 2004; 292: 1205-12.
35． 《1974 年痘苗经验交流会资料汇编》
36. Smallpox Vaccination and Adverse Reactions Public Health Guidance for Clinicians. A Teaching Set Reviewing Key Points from the February 21, 2003 MMWR Recommendations and Reports.www.bt.cdc.gov/agent/smallpox
37． 《药物临床试验质量管理规范》国家食品药品监督管理局（局令第 3 号）
