# Supplementary material for: Safety and immunogenicity of an HIV vaccine trial with DNA prime and replicating vaccinia boost
Source: Signal Transduct Target Ther. 2025 Jul 2;10:208. doi: 10.1038/s41392-025-02259-y (PMC12217030; doi:10.1038/s41392-025-02259-y)
Supplement: Supplementary file 5 — IRB approvals [file 41392_2025_2259_MOESM5_ESM.pdf]

附件 3

北京协和医院药物临床试验伦理委员会审核表

<table>
<tr><td>药物名称</td><td colspan="3">DNA-天坛痘苗复合型艾滋病疫苗</td><td>注册分类</td><td>预防用生物制品 1 类</td><td>申请事项</td><td>新药临床 I 期</td></tr>
<tr><td colspan="4">申请人：中国疾病预防控制中心性病艾滋病预防控制中心，北京生物制品研究所</td><td colspan="4">任务来源：SFDA</td></tr>
<tr><td>专业组</td><td colspan="3">感染科</td><td colspan="2">主要研究者</td><td colspan="2">李太生</td></tr>
<tr><td>会议地点</td><td colspan="3">北京协和医院老楼 11 号楼 1 层临床药理研究中心活动室</td><td colspan="2">日　　期</td><td colspan="2">2007/3/21</td></tr>
<tr><td>主任委员</td><td colspan="3">鲁重美</td><td colspan="2">副主任委员</td><td colspan="2">翟晓梅</td></tr>
<tr><td colspan="8">到会委员：鲁重美、蔡金生、曾文莉、江骥、叶铁虎、郑凯、李大魁、单渊东</td></tr>
<tr><td colspan="2">药审批件及编号</td><td colspan="6">2006L04 .76</td></tr>
<tr><td colspan="2">药检报告及批号</td><td colspan="6">20041123,2004112:4,20050318</td></tr>
<tr><td rowspan="2">临床前及临床资料</td><td>药　学</td><td>✓</td><td>药效学</td><td>✓</td><td>药理学</td><td colspan="2">✓</td></tr>
<tr><td>毒理学</td><td>✓</td><td>特殊毒理学</td><td>✓</td><td>临　床</td><td colspan="2">✓</td></tr>
<tr><td colspan="8">主要研究者资格评价：
具较丰富的感染科临床经验，长期从事艾滋病的基础及临床研究，曾获华西医科大学临床药理培训班结业证，参加过药品临床试验。</td></tr>
<tr><td colspan="8">研究方案名称：
艾滋病疫苗（DNA 疫苗与重组天坛痘苗联合使用）I 期临床试验方案</td></tr>
<tr><td colspan="8">设计方案评价：（版本 1.0，2007 年 2 月 26 日）
先进行 Ia 期，方案设计基本合理、可行，建议严格志愿者的入选，包括 HIV 抗体及核酸检测，精神评定，排除艾滋病者感染窗口期人群，排除吸毒、药物依赖、吸烟、酗酒者。</td></tr>
<tr><td colspan="8">知情同意书评价：（版本 1.0，2007 年 2 月 26 日）；受试者招募广告及宣传单
基本符合要求。</td></tr>
<tr><td colspan="8">受试者补偿措施评价：
补充“承诺发生与试验有关严重不良反应，造成健康损害的补偿责任”。</td></tr>
</table>

<table>
<tr><td colspan="3">同意临床试验者签字</td><td>不同意临床试验者签字</td></tr>
<tr><td colspan="3">[illegible]</td><td></td></tr>
<tr><td rowspan="3">结论</td><td>1. 同意。</td><td></td><td>正主任委员：鲁重美 [illegible]</td></tr>
<tr><td>2. 作必要修改后同意。</td><td>✓</td><td>副主任委员：翟晓梅</td></tr>
<tr><td>3. 不同意。</td><td></td><td>记录人：单渊东 [illegible]</td></tr>
</table>

回　　执

已收到中国疾病预防控制中心艾滋病预防控制中心艾滋病疫苗 I 期临床试验研究方案修改版，同意目前修改方案。

北京协和医院药物临床试验伦理委员会

[illegible]
2007-11-26

北京协和医院药物临床试验医学伦理委员会

姓名	性别	出生年月	工作单位	职务	职称	电话
鲁重美	女	1949.8	北京协和医院	党委书记、副院长	教授	65296810
翟晓梅	女	1956.12	中国医学科学院/协和医科大学		副教授	65296484
蔡金生	男	1943.6	中华全国工商业联合会	副秘书长		65275337
曹文莉	女	1967.5	北京金德律师事务所		律师	65001188-3762
单渊东	男	1941.1	北京协和医院临床药理研究中心	顾问	教授	65296568
李舜伟	男	1936.11	北京协和医院神经科		教授	65296371
游　凯	男	1933.3	北京协和医院心内科		教授	65295061
李大魁	男	1944.8	北京协和医院药剂科	主任	教授	65296510
江　骥	男	1954.3	北京协和医院	“中心”实验室主任	教授	65296573
叶铁虎	男	1946.8	北京协和医院临床药理研究中心	主任	教授	65295581
崔丽英	女	1956.12	北京协和医院神经科	副主任	教授	65296575

本伦理委员会成员名单自 2006 年 10 月 25 日起生效，有效期暂定 5 年。

伦理委员会主任委员：[illegible]

中国疾病预防控制中心

性病艾滋病预防控制中心伦理委员会

项目评审报告

项目编号：KX070420111

经中国疾病预防控制中心性病艾滋病预防控制中心伦理委员会专家评审后，认为下列项目符合我国伦理学方面的要求，项目可以进行实施。请特别注意落实保护受试者的各项措施。

项目名称：艾滋病疫苗（DNA 疫苗与重组天坛痘苗联合使用）I 期临床试验方案

项目负责人：李太生　教授

单　　位：　北京协和医院感染内科

评审日期：2007 年 4 月 27 日

批准日期：2007 年 4 月 27 日

主席：（签字）王若涛

王若涛

中国疾病预防控制中心

性病艾滋病预防控制中心伦理委员会

中国性病艾滋病预防控制中心伦理审查委员会名单

中国性病艾滋病预防控制中心已经获得美国联邦广泛登记号（FWA # 00002958和FWA # 00006661），中国性病艾滋病预防控制中心伦理审查委员会登记号分别为（IRB00002276）。中国性病艾滋病预防控制中心伦理审查委员会对整个研究进行监督。

中国性病艾滋病中心伦理审查委员会人员名单

姓名	工作单位	职称	学科
王若涛	中国疾控中心性艾中心	研究员	卫生法学
张孔来	中国协和医科大学	教授	社会医学家
彭瑞聪	北京大学	教授	公共卫生学家
翟小梅	中国医学科学院	教授	伦理学家
邱仁宗	社会科学院	教授	伦理学家
戴志澄	性病艾滋病协会	教授	NGO
徐莲芝	佑安医院	主任医师	社会医学家工作者
张福杰	中国疾控中心性艾中心	主任医师	艾滋病治疗
余东保	中国疾控中心性艾中心	研究员	艾滋病健康教育
孙江平	中国疾控中心性艾中心	研究员	艾滋病健康教育
汪宁	中国疾控中心性艾中心	教授	艾滋病流行病学
邬蕾	中国疾控中心性艾中心	秘书	

北京协和医院药物临床试验伦理委员会审核表

药物名称	艾滋病疫苗(核酸与重组天坛疫苗联合使用)	注册分类	预防用生物制品	申请事项	补充申请

申请人:<br>中国疾病预防控制中心性病艾滋病预防控制中心，北京生物制品研究所	任务来源:<br>SFDA

专业组	感染科	主要研究者	李太生
会议地点	北京协和医院临床药理研究中心I期临床试验研究室(协和明日交流中心七层会议室)	日期	2008/08/28
主任委员	鲁重美	副主任委员	翟晓梅

到会委员: 鲁重美、江骥、曹文莉、游凯、叶铁虎、李大魁、蔡金生、张抒扬、单渊东

药审批件及编号	2008L04306
药检报告及批号	20050318，20050318，20041123，20041124，20061101，20070927，食药监注便函〔2007〕470号

临床前及临床资料	药学	✓	药效学	✓	药理学	✓
	毒理学	✓	特殊毒理学	✓	临床	✓

主要研究者资格评价:

具较丰富的感染科临床经验，长期从事艾滋病的基础及临床研究，曾获华西医科大学临床药理培训班结业证，参加过药品临床试验。

研究方案名称:

艾滋病疫苗(核酸疫苗与重组天坛痘苗联合使用)Ib期临床试验方案

设计方案评价:

基本合理、可行。应密切监测安全性。

知情同意书评价:

基本符合要求。

受试者补偿措施评价:

基本符合要求。

同意临床试验者签字	不同意临床试验者签字

结论	1. 同意。		正主任委员: 鲁重美
	2. 作必要修改后同意。		副主任委员: 翟晓梅
	3. 不同意。		记录人: 单渊东

*经本伦理委员会审批的文件清单详见附件。

附件：

资料 1　Ib 期临床批件

资料 2　试验药品概述

资料 3　药品检验报告及效期延长批件

资料 4　研究者手册

资料 5　Ib 期临床试验方案

日期：2008 年 8 月 22 日　版本号：1.0

资料 6　CRF 样表

资料 7　招募广告和招募启事

资料 8　艾滋病疫苗（核酸疫苗与重组天坛痘苗联合使用）
Ia 期临床试验急性观察阶段总结报告

注：受试者需知和知情同意书见资料 5（Ib 期临床试验方案）的“附件 1”

北京协和医院药物临床试验伦理委员会

主任委员（签名）

日期：2008 年 8 月 28 日

北京协和医院药物临床试验伦理委员会

姓名	性别	出生年月	工作单位	职务	职称	电话
鲁重美	女	1949.8	北京协和医院	党委书记、副院长	教授	65296810
翟晓梅	女	1956.12	中国医学科学院/协和医科大学		副教授	65296484
蔡金生	男	1943.6	中华全国工商业联合会	副秘书长		65275337
曹文莉	女	1967.5	北京金德律师事务所		律师	65001188-3762
单渊东	男	1941.1	北京协和医院临床药理研究中心	顾问	教授	65296568
李舜伟	男	1936.11	北京协和医院神经科		教授	65296371
游　凯	男	1933.3	北京协和医院心内科		教授	65295061
李大魁	男	1944.8	北京协和医院药剂科	主任	教授	65296510
江　骥	男	1954.3	北京协和医院	“中心”实验室主任	教授	65296573
叶铁虎	男	1946.8	北京协和医院麻醉科		教授	65295581
崔丽英	女	1956.12	北京协和医院神经科	副主任	教授	65296575
张抒扬	女	1963.3	北京协和医院心内科<br>北京协和医院临床药理研究中心	副主任<br>主任	教授	65295069

伦理委员会主任委员：鲁重美 2008.5.7.

副主任委员：翟晓梅 2008.5.7.

中国疾病预防控制中心
性病艾滋病预防控制中心伦理委员会
项目评审报告

项目编号：**X0800901142**

经中国疾病预防控制中心性病艾滋病预防控制中心伦理委员会专家评审后，认为下列项目符合我国伦理学方面的要求，项目可以进行实施。

项目名称:艾滋病疫苗（核酸疫苗与重组天坛痘苗联合使用）Ib 期临床试验

项目负责人：邵一鸣 研究员

单　　位：　中国疾病预防控制中心 性病艾滋病预防控制中心

评审日期：2008 年 9 月 23 日

批准日期：2008 年 9 月 23 日

主席: (签字) 王若涛

王若涛

中国疾病预防控制中心

性病艾滋病预防控制中心伦理委员会
